# C5-Substituted 2-Selenouridines Ensure Efficient Base Pairing with Guanosine; Consequences for Reading the NNG-3′ Synonymous mRNA Codons

**DOI:** 10.3390/ijms21082882

**Published:** 2020-04-20

**Authors:** Grazyna Leszczynska, Marek Cypryk, Bartlomiej Gostynski, Klaudia Sadowska, Paulina Herman, Grzegorz Bujacz, Elzbieta Lodyga-Chruscinska, Elzbieta Sochacka, Barbara Nawrot

**Affiliations:** 1 Institute of Organic Chemistry, Faculty of Chemistry, Lodz University of Technology, Zeromskiego 116, 90-924 Lodz, Poland; grazyna.leszczynska@p.lodz.pl (G.L.); klaudia.sadowska@edu.p.lodz.pl (K.S.); 197394@edu.p.lodz.pl (P.H.); elzbieta.sochacka@p.lodz.pl (E.S.); 2 Centre of Molecular and Macromolecular Studies, Polish Academy of Sciences, Sienkiewicza 112, 90-363 Lodz; Poland; mcypryk@cbmm.lodz.pl (M.C.); bgostyns@cbmm.lodz.pl (B.G.); 3 Institute of Molecular and Industrial Biotechnology, Lodz University of Technology, 4/10 Stefanowskiego St., 90-924 Lodz, Poland; grzegorz.bujacz@p.lodz.pl; 4 Institute of General Food Chemistry, Lodz University of Technology, 4/10 Stefanowskiego St., 90-924 Lodz, Poland; elzbieta.lodyga-chruscinska@p.lodz.pl

**Keywords:** modified uridine, wobble modification, seleno-uridine, selenium, tRNA, anticodon, synonymous codons, translation regulation, decoding, new wobble base pair

## Abstract

5-Substituted 2-selenouridines (R5Se2U) are post-transcriptional modifications present in the first anticodon position of transfer RNA. Their functional role in the regulation of gene expression is elusive. Here, we present efficient syntheses of 5-methylaminomethyl-2-selenouridine (**1**, mnm5Se2U), 5-carboxymethylaminomethyl-2-selenouridine (**2**, cmnm5Se2U), and Se2U (**3**) alongside the crystal structure of the latter nucleoside. By using pH-dependent potentiometric titration, p*K*a values for the N3H groups of **1**–**3** were assessed to be significantly lower compared to their 2-thio- and 2-oxo-congeners. At physiological conditions (pH 7.4), Se2-uridines **1** and **2** preferentially adopted the zwitterionic form (**ZI**, ca. 90%), with the positive charge located at the amino alkyl side chain and the negative charge at the Se2-N3-O4 edge. As shown by density functional theory (DFT) calculations, this **ZI** form efficiently bound to guanine, forming the so-called “new wobble base pair”, which was accepted by the ribosome architecture. These data suggest that the tRNA anticodons with wobble R5Se2Us may preferentially read the 5′-NNG-3′ synonymous codons, unlike their 2-thio- and 2-oxo-precursors, which preferentially read the 5′-NNA-3′ codons. Thus, the interplay between the levels of U-, S2U- and Se2U-tRNA may have a dominant role in the epitranscriptomic regulation of gene expression via reading of the synonymous 3′-A- and 3′-G-ending codons.

## 1. Introduction

**Figure 1:**
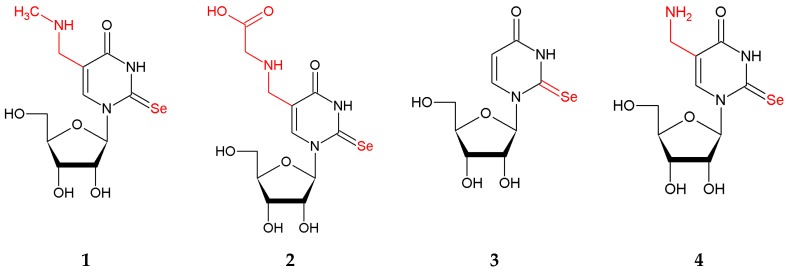
Structure of 2-selenouridine units naturally occurring in the wobble position of transfer RNA *iso*-acceptors specific for Lys, Glu, and Gln: 5-methylaminomethyl-2-selenouridine (mnm5Se2U, **1**), 5-carboxymethylaminomethyl-2-selenouridine (cmnm5Se2U, **2**), 2-selenouridine (Se2U, **3**), and 5-aminomethyl-2-selenouridine (nm5Se2U, **4**).

Presently known transfer RNAs contain more than 100 modified nucleosides, which constitute the vast majority of the 160 total modified units identified thus far in cellular RNAs. Approximately 50% of them are present at position 34 of tRNAs, also known as the wobble position of the anticodon [1,2]. They are involved in the fine-tuning of protein biosynthesis through modulation of codon–anticodon interactions and enhance the capability of tRNA to select the appropriate synonymous mRNA codon [3,4,5,6,7]. 5-Substituted uridines (R5Us), 2-thiouridines (R5S2Us) and 2-selenouridines (R5Se2Us) are the most widespread types of modified wobble nucleosides and are present in all three domains of life. To date, sulfur- and selenium-containing uridines have been found only in anticodons of tRNA *iso*-acceptors specific for glutamate, glutamine, and lysine [8]. Four selenium-containing uridines, namely, 5-methylaminomethyl-2-selenouridine (mnm5Se2U, **1**), 5-carboxymethylaminomethyl-2-selenouridine (cmnm5Se2U, **2**), 2-selenouridine (Se2U, **3**), and 5-aminomethyl-2-selenouridine (nm5Se2U, **4**), were identified in bacterial tRNAs (Figure 1), with mnm5Se2U (**1**) being the most abundant [1,9,10,11,12]. Se-containing tRNAs were also found in mammalian cells (mouse leukemia cells [13], bovine liver cells [14]), archaea (*Methanococcus vannielii*) [15,16], and plants (germinating barley [17], *Chlamydomonas*, wild carrot, tobacco, bamboo, and rice cells as well as mung bean and soybean seedlings [18]), although the exact locations of Se atoms have not been described thus far.

The biosynthesis pathway of 2-selenouridines in bacterial tRNAs was initially considered to be similar to that of the U→S2U conversion, i.e., the deselenation of L-selenocysteine (Sec) by the selenium-specific analogue of cysteine desulfurase (lscS) [19], followed by incorporation of selenium into uridine through the action of a 2-selenouridine-specific synthetase, which was assumed to be analogous to 2-thiouridine synthetase (MnmA) [20]. According to a later proposal, bacteria synthesize selenium-modified tRNAs using the selenophosphate anion [11] (as a donor of Se) and the corresponding 2-thio precursors in a reaction catalyzed by tRNA 2-selenouridine synthase (SelU or MnmH), which is the product of *ybbB* gene expression in *Escherichia coli* [11,21]. YbbB orthologues were also identified in mammals [14] and archaea (*Methanococcus maripaludis*) [22]. Interestingly, in numerous bacteria (*Escherichia coli*, *Enterobacter aerogenes*, *Pseudomonas aeruginosa,* and *Salmonella enterica* var. Typhimurium), along with the selenium-modified uridine, *S*-geranylated derivatives of 2-thiouridine (mnm5geS2U and cmnm5geS2U) were found, although in relatively low abundance (up to 7%) [21,23]. As noted recently, tRNA 2-selenouridine synthase SelU, i.e., the enzyme responsible for S→Se replacement, also catalyzes the *S*-geranylation of 2-thiouridine in tRNAs. Contrary to earlier suggestions, it was recently demonstrated that *S*-geranylated tRNA primarily acts as the intermediate in S2U-tRNA→Se2U-tRNA transformation [24,25] and does not seem to serve as an amino acid carrier at the ribosome or bind to bacterial cell membranes [21,23,26].

For many years, synthetic 2-selenouridine nucleosides were barely accessible. The first method to attempt this achievement was based on the glycosylation of silylated 2-selenouracil, by which 2-selenouridine (Se2U, **3**) [27] and 5-methylaminomethyl-2-selenouridine (mnm5Se2U, **1**) [10] were obtained in low coupling yield. The second method, which was slightly more effective, was based on 2-Se functionalization of *iso*-cytidine with highly toxic H_2_Se [28]. Currently, the most effective strategy of 2-selenium incorporation relies on *S*-alkylation (*S*-methylation or *S*-geranylation) of sugar-protected 2-thiouridine, followed by thioalkyl→SeH substitution with sodium hydrogen selenide (NaSeH) [24,29,30]. Consequently, effective synthesis of a phosphoramidite derivative of suitably protected Se2U was developed, and numerous synthetic Se2U-RNAs were obtained for structural, physicochemical and functional studies [25,29,30]. Huang and co-workers, who pioneered the chemical synthesis of various Se-derivatives of nucleic acids [31], also developed enzymatic synthesis of Se2U-RNAs utilizing 2-selenouridine triphosphate (Se2UTP) and RNA transcription [32].

In terms of structural properties, S2U and Se2U are significantly different from uridine because the S and Se atoms have much larger bonding atomic radii (1.05 Å and 1.20 Å, respectively) than oxygen (0.66 Å). Increasing the repulsive interactions between the chalcogen atom at the C2 of nucleobase and the 2′-hydroxyl group of the ribose ring is thought to lead to an increased abundance of the C3′-*endo* conformation of the sugar ring (53% in uridine, 71% in 2-thiouridine, and 80% in 2-selenouridine) [30,33,34,35,36]. The larger size of sulfur and selenium atoms also strengthens the stacking interactions between the given nucleoside and the neighboring nucleobases in RNA duplexes [29].

Regarding the specificity of hybridization, uridines in the RNA chain preferentially recognize A by Watson–Crick interactions and, with lower affinity, read G complements by wobble hydrogen bond patterns [37]. Introduction of sulfur or selenium atoms at position 2 of uridine enhances the thermodynamic stability of RNA duplexes with X2U–A base pairs and restricts the formation of X2U–G base pairs (X = S or Se); this effect is more pronounced for Se2U than for S2U models [29]. In contrast to the above results of thermodynamic measurements, biological experiments demonstrated that synonymous mRNA codons with 3′-A- and 3′-G-ending units are equally well recognized by tRNAs with anticodons containing wobble modified uridines, especially those containing sulfur at position 2 and bearing specific substituents at position 5 [20,38,39]. Recent data suggested that in a biological context (e.g., tRNA bound to mRNA at the ribosome), modified uridine 34 units (U*_34_) can recognize guanosine units by at least two different hydrogen bonding patterns, depending on the substituents at positions 2 and 5 of the uracil ring [40,41,42,43,44,45]. While U*_34_–G base-pairing data are available for 5-substituted uridines and 2-thiouridines, data for corresponding 5-substituted 2-selenouridines identified in tRNAs are limited [46,47].

The ultimate goal of the present work is to assess the binding affinity of R5Se2U to A and G complements and to understand why Nature introduced selenium atoms instead of sulfur atoms in the wobble uridines of specific tRNAs. Here, we demonstrate an efficient method of synthesizing native selenium-modified uridines 5-substituted with methylaminomethyl (mnm5Se2U, **1**) and carboxymethylaminomethyl (cmnm5Se2U, **2**) side chains and describe their physicochemical and structural properties. Additionally, the crystallographic structure of the Se2U (**3**) nucleoside is discussed in light of the crystallographic data of its parent congeners, U and S2U. The experiments are supported by computational investigations to analyze the base pairing of Se2-uridines with purine nucleosides. The obtained data are used to elucidate the influence of R5 and Se2 substituents on the structure of R5Se2U and its binding to A/G and to determine which function of R5S2Us in tRNA is disabled but fully ensured by R5Se2Us to provide the reading of synonymous 5′-NNG-3′ mRNA codons.

## 2. Results

### 2.1. Chemistry

**Scheme 1:**
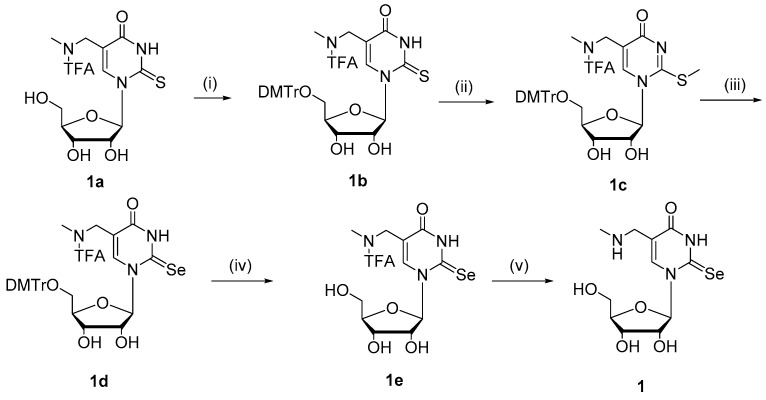
Synthesis of 5-methylaminomethyl-2-selenouridine (**1**). Reagents and conditions: (i) DMTr-Cl, pyridine, rt, overnight, 92%; (ii) MeI, Et_3_N, EtOH, rt, 2h, 90%; (iii) Se, NaBH_4_, EtOH, 2 h, 0 °C to generate NaSeH, then rt, 2.5 h, 60%; (iv) 50% aq. AcOH, 1.5 h, rt, 70%; (v) 30% aq. ammonia, 15 min, rt, 83%. TFA: trifluoroacetyl; DMTr: 4,4′-dimethoxytrityl.

**Scheme 2:**
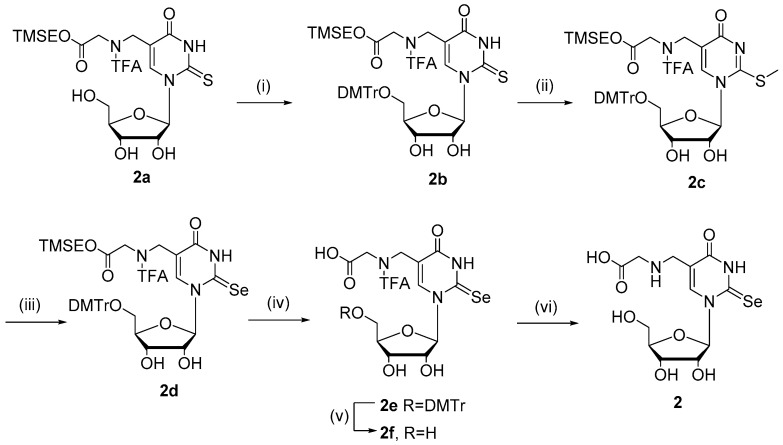
Synthesis of 5-carboxymethylaminomethyl-2-selenouridine (**2**). Reagents and conditions: (i) DMTr-Cl, pyridine, rt, overnight, 90%; (ii) MeI, Et_3_N, EtOH, 2 h, rt, 80%; (iii) Se, NaBH_4_, EtOH, 2 h, 0 °C to generate NaSeH, then rt, 1 h, 84%; (iv) 1 M TBAF in THF, 50 min, rt; (v) 50% aq. AcOH, 1 h, rt, 70%; (vi) 30% aq. ammonia, 1 h, rt, 82%. TFA: trifluoroacetyl; TMSE: 2-(trimethylsilyl)ethyl; DMTr: 4,4′-dimethoxytrityl.

Our syntheses of mnm5Se2U (**1**) (Scheme 1) and cmnm5Se2U (**2**) (Scheme 2) began with the 5-substituted 2-thiouridines **1a** and **2a**, respectively. 5-Methylaminomethyl-2-thiouridine (**1a**) with *N*-trifluoroacetyl (TFA) protection of the amine functional group at the 5-position and 5-carboxymethylaminomethyl-2-thiouridine (**2a**) with *N*-TFA protection and a 2-(trimethylsilyl)ethyl (TMSE) ester-type protecting group on the carboxylic function were obtained according to previously described procedures [48,49,50,51,52]. Before the *S*-alkylation step, substrates **1a** and **2a** were subjected to 5′-*O*-dimethoxytritylation and treated with methyl iodide in the presence of triethylamine in ethanol to obtain *S*-methylated 2-thiouridines **1c** and **2c** in yields of 90% and 80%, respectively. The *S*-methylated derivative of mnm5S2U (**1c**) was subsequently treated with NaSeH (10 equivalents (equiv.)) prepared via a sodium borohydride reduction of elemental selenium (15 equiv.) in EtOH according to the procedure originally elaborated by Klayman and Griffin [53] and utilized by Huang’s and Davis’s groups to prepare 2-selenated thymidine and uridine phosphoramidites [29,30,54]. Since seleno-compounds are sensitive to oxygen, all the reactions were conducted under an argon atmosphere. In our work, the selenation of *S*-methylated mnm5S2U **1c** was performed at room temperature for 2.5 h, yielding 60% 2-selenouridine **1d**. The use of a higher excess of NaSeH (12 equiv.) in the reaction with *S*-methylated cmnm5S2U **2c** resulted in complete selenation after 1 h (TLC control), and the seleno-compound **2d** was isolated in a yield of 84% after purification by flash column chromatography using argon overpressure.

In further steps involving the removal of protecting groups from 5′-*O*-DMTr-*N*-TFA-selenonucleoside **1d** (Scheme 1), 5′-*O*-DMTr was removed under aqueous acidic conditions (70% yield), then the TFA group was removed by deacylation in aqueous ammonia (83% yield) to give the desired selenonucleoside **1** after purification by silica gel column chromatography. The total yield of the synthesis of 2-selenouridine **1** in the four-step procedure starting from its 2-thiouridine precursor **1b** was 31%.

In the case of 5′-*O*-DMTr-*N*-TFA-TMSE-protected cmnm5Se2U **2d** (Scheme 2), the carboxylic group was released in the first step using a standard 1 M solution of tetrabutylammonium fluoride (TBAF) in THF. After 1 h, we observed full conversion of **2d** to carboxy-deprotected selenouridine **2e**, which exhibited significantly lower mobility on a TLC plate. To remove the excess TBAF, a sulfonic acid resin (DOWEX, form H^+^) and calcium carbonate were added, with methanol as a solvent [55]. After removal of the TBAF-derived material (NMR control), the crude product **2e** was detritylated with 50% aqueous (aq.) AcOH (1 h, room temperature (rt)) to obtain **2f** in 70% yield. The ammonolysis of *N*-TFA-protected selenonucleoside **2f** (30% aq. ammonium, 1 h, rt) completed the synthesis of cmnm5Se2U (**2**). The final cmnm5Se2U **2** was purified by reverse phase high performance liquid chromatography (RP-HPLC) on a preparative C18 column, yielding the product at 82%. The total yield of the synthesis of 2-selenouridine **2** in the four-step procedure starting from its 2-thiouridine precursor **2b** was 38%.

The structures of synthetic selenouridines **1** and **2** were fully confirmed by NMR and UV analyses (spectra presented in Supplementary Materials Figures S1–S25).

### 2.2. Physicochemical Analysis

#### 2.2.1. Effect of pH on the Structure of 2-Selenouridines **1**–**3** and Their Thio-Precursors **5**–**7**

**Figure 2:**
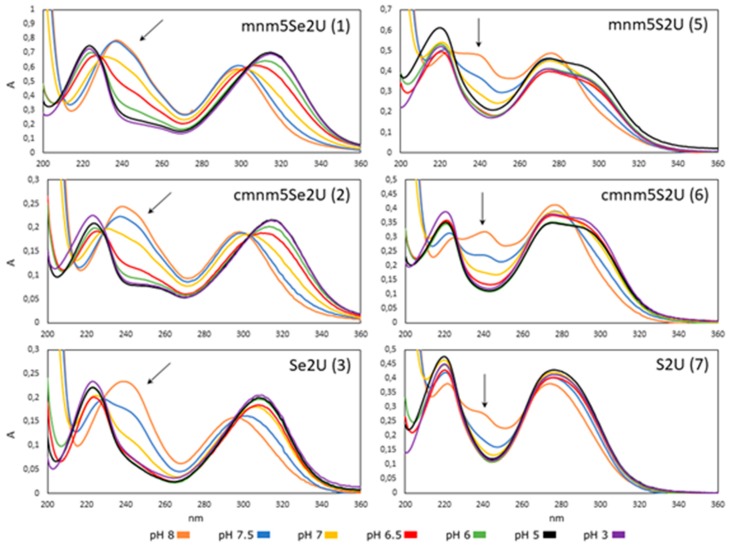
UV spectra of 2-selenouridines **1–3** and 2-thiouridines **5–7**, taken at increasing pH in 10 mM HCl at pH 3.0 and 67 mM Na_2_HPO_4_/KH_2_PO_4_ buffer at pH 5.0, 6.0, 6.5, 7.0, 7.5, and 8.0.

The effect of pH on the structure of 2-selenouridines **1**–**3** and their thio-precursors **5**–**7** was analyzed by UV spectroscopy. The UV absorption coefficients and absorbance maxima of 2-selenouridines (**1–3**) and 2-thiouridines (**5–7**) were assessed (Table 1). Both series of UV spectra for **1–3** and **5–7**, which were acquired at increasing pH (from pH 3 to pH 8), differed significantly, as shown in Figure 2. The UV spectra of 2-selenouridines showed characteristic shifts of the absorbance maxima towards longer wavelengths at acidic, neutral, and basic pH in comparison to their 2-thio analogues (Figure 2, Table 1). However, both Se2- and S2-uridines exhibited the same maximum absorbance at 240 nm when the pH was changed from acidic to basic values. In the case of 5-substituted 2-selenouridines compared with 2-thiouridines, this peak exhibited higher absorbance and appeared at slightly lower pH.

#### 2.2.2. p*K*a Determination and Analysis of the Content of the Ionized Fraction of **1**–**3**

The p*K*a values for 2-seleno-nucleosides **1–3** (Table 2) were calculated from the respective pH-potentiometric titration curves using an improved SUPERQUAD program [56]. For all screened compounds, the p*K*a values were obtained for their N3H groups as well as for the functional groups present in the 5-side chains of **1** and **2** (-NHCH_2_- and -COOH). The p*K*a value for N3H in 2-selenouridine **3** (7.30) was much lower than that for uridine (p*K*a 9.15) and 2-thiouridine (p*K*a 8.09). Furthermore, due to the presence of electron-withdrawing amino alkyl side chains, which are protonated (-NH_2_^+^CH_2_-, pKa > 9.0) at physiological pH, the ability of the N3H proton to depart was increased. Thus, the p*K*a values of the N3H groups of **1** and **2** decreased to 6.43 and 6.55, respectively. A slightly lower acidity of the N3H of selenium derivative **2** was observed due to the presence of the negatively charged carboxyl group, resulting in a decrease in the electron-withdrawing character of the 5-cmnm side chain. After determining the p*K*a values of the N3H groups of **1**, **2,** and **3**, the content of the ionized fractions of seleno-nucleosides **1**, **2,** and **3** was calculated based on data obtained from the pH-dependent potentiometric titrations, analogous to the previously used method (45). Thus, the values of ionized fractions of **1–3** at physiological pH (7.4) were calculated from the Henderson–Hasselbalch equation, p*K*a – pH = log[BH]/[B^-^], where BH and B^-^ are the neutral and ionized (deprotonated) forms, respectively [57]. The obtained results (Table 2, data given in square brackets) indicated that the 2-selenonucleoside units bearing substituents containing a positively charged protonated amino methyl group, as in **1** and **2**, exist predominantly (approximately 90%) in the N3-deprotonated (ionized) form. For the 5-unsubstituted 2-selenouridine **3,** the ionized fraction was assessed to be approximately 58%. Since the p*K*a values of the N3H groups in pyrimidine nucleotides (bearing a negatively charged phosphate group) were higher by approximately 0.4 units than those in the corresponding nucleosides [58], we recalculated the content of the ionized fraction of the corresponding nucleotides using the p*K*a values measured here, which increased by 0.4 units (Table 2, data given in brackets in italics). In this way, we obtained slightly lower numbers for the ionized form content, but this fraction was still predominant.

### 2.3. Structural Analysis

#### 2.3.1. Se2U Crystal Structure

**Figure 3:**
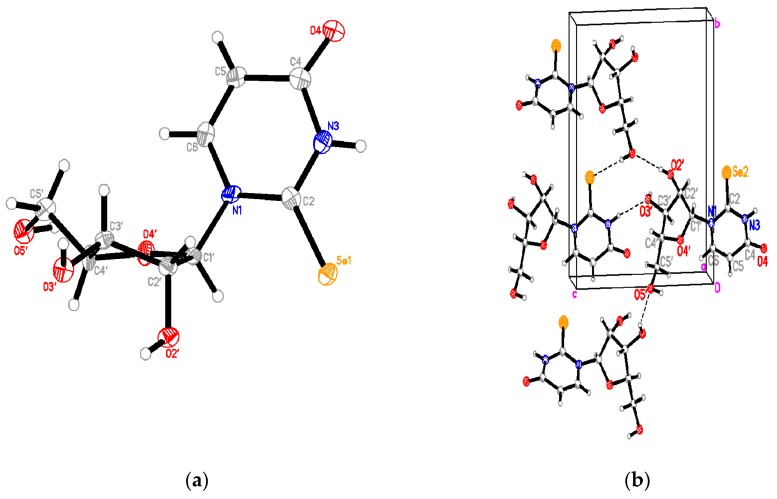
The ORTEP drawing of 2-selenouridine (Se2U, **3**) (**A**) and the crystal packing of the nucleoside viewed along the *a* axis (**B**). Hydrogen bonds are indicated by dashed lines. Hydrogen atoms are represented by circles with an arbitrary radius. Displacement ellipsoids were drawn at the 50% probability level.

The molecular structure of 2-selenouridine (**3**) was determined by X-ray diffraction analysis (Figure 3). The Oak Ridge Thermal Ellipsoid Plot (ORTEP) drawing of Se2U is presented in Figure 3A. Selected crystallographic data for Se2U in comparison with S2U [59] and U [60] are given in the Supplementary Materials (Tables S1–S6). The Se2U nucleoside crystallized in one of the typical crystallographic space groups for chiral compounds, namely P2_1_, with one molecule in the asymmetric unit (Table S1). The crystal packing, reflected in the unit cell dimensions with a relatively short a axis, was stabilized by four intermolecular hydrogen bonds (Figure 3B, Table S2). The geometry of the 2-selenouracil heterobase moiety of Se2U was slightly changed in comparison to those of the 2-thiouracil or uracil residues in the S2U [59] and U structures [60]. The Se atom was displaced out of the heterobase ring plane by 0.234 Å, while the S atom in S2U and the O atom in U exhibited planar alignments (Table S2). The length of the C2-Se bond (1.851(8) Å) in the Se2U molecular structure corresponds to neither a pure C=Se bond (1.74 Å) nor a single C-Se bond (1.94 Å) [61]. The selenol tautomer (C2-SeH), however, was rejected by crystallographic refinement; when hydrogen was modeled in place of the higher residual peak in the vicinity of the selenium atom and removal of the N3 hydrogen, the refinement parameters increased significantly.

Conformational analysis of 2-selenouridine (**3**) was carried out based on general parameters defined by Altona and Sundaralingam [62]. The selected torsion angles for Se2U and 2-thiouridine [59] and two crystals of uridine (A and B) [60], which are crucial for nucleoside conformation assignments, are listed in Table S6, while the resulting conformational data are shown in Table 3. The sugar of the Se2U residue adopted a C3′-*endo* N-conformation (^3^E), with a phase angle of pseudorotation of P = 14.4° and a pseudorotation amplitude of 34.7°, which is often observed in the structure of RNA nucleosides and was also present in the crystal structures of the reference S2U and U nucleosides. The orientation of the heterocyclic base relative to the sugar moiety in Se2U molecules (χ = −146.5°) was in the typical *anti* range, similar to the S2U and U structures (Table 3). The conformation around the C4′-C5′ ribose bond of Se2U was in *trans* arrangement, which is observed less frequently than the gauche (+) structure in pyrimidine nucleosides which present an N-type sugar ring pucker [63]. The preference of Se2U to exist as the C4′-C5′ *trans* conformer is due to the hydrogen bonds involving the 5′-OH sugar group, similar to the hydrogen bond pattern displayed in the crystals of the S2U molecule [59].

#### 2.3.2. Structural Analysis of Sugar Residues of **1–3** in Solution

The conformational preference of the ribose rings in selenouridines **1–3** in solution was experimentally confirmed by ^1^H NMR spectroscopy and compared to data obtained for their 2-thio analogues **5–7 [35]** and their uridine precursors. The sulfur modification in 2-thiouridines was shown to generally stabilize the C3′-*endo* conformation of the sugar ring because of steric repulsion between the sulfur atom and the oxygen atom of the 2′-hydroxyl group [33,64,65]. Thus, the replacement of the sulfur atom with a larger selenium atom should increase the steric demand upon contact with the 2′-OH group and stabilize the C3′-*endo* sugar conformation even more. As shown in Table 4, only 2-selenouridine **3** exhibited an increased content of the C3′-*endo* form (80%) in comparison to 2-thiouridine **7** (71%). However, the remaining 2-selenouridines **1** and **2** adopted the C3′-*endo* conformation at a slightly lower rate than their 2-thio-congeners **5** and **6**.

### 2.4. Molecular Modeling

To understand the specific role of selenium in nucleic acid chemistry, we used theoretical DFT calculations to model the properties of 2-selenouracil (m1mnm5Se2Ura) and its complexes with guanine and to compare the results with those of previously modeled uracil (m1mnm5Ura) and 2-thiouracil (m1mnm5S2Ura). The ribose moiety in model compounds was replaced by a methyl group to reduce the computational cost. We focused on tautomerization energy, some structural features, such as bond distances and atomic charges, and the enthalpy of base-pair formation with guanine.

#### 2.4.1. Structural Analysis of m1Se2Ura and m1mnm5Se2Ura Tautomers

We identified the same series of tautomeric forms of Se2Ura nucleobases as for 2-oxo- and 2-thio-analogues, 2,4-diketo- (K), 2-enol,4-keto- (E2), 2-keto-4-enol (E4), and the zwitterionic (ZI) form [45] (see Figure S28). Structural analysis of Se2Ura tautomers in comparison with the Ura and S2Ura tautomers revealed no significant changes in bonding parameters, except for the C2-X bond length and the size of the heteroatom. The series of m1R5X2Ura derivatives (X = O, S, and Se and R = H, mnm) showed the same trends in variation of the C2-X bond lengths among tautomers, e.g., for ZI the length of the C2-Se bond was the longest (1.866 Å) while the C2-S and C2-O bonds were shorter (1.726 and 1.250 Å, respectively, see Figure S29 and Table S7).

#### 2.4.2. Gibbs Free Energies of the Tautomers of 1-Methyl-2-Selenouracil and 1-Methyl-5-Methylaminomethyl-2-Selenouracil

**Figure 4:**
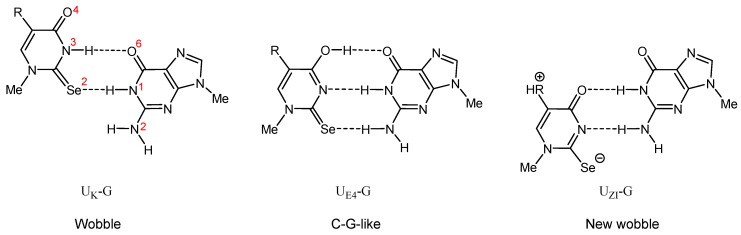
The structures of the complexes between m1R5Se2Ura and m9Gua. The wobble and C–G-like base pairs represent the complexes formed between the **K** and **E4** tautomers of the pyrimidine nucleobase models and m9Gua, respectively. The new wobble base pair represents the most stable complex of the zwitterionic form of m1mnm5Se2Ura with and m9Gua (with the protonated C5-mnm side chain).

The Gibbs free energies (G) of the diketo (**K**) and two keto-enol tautomeric forms (**E2** and **E4**), as well as the zwitterionic forms (**ZI**) (Figure S28, Figure 4), were calculated in the gas phase and in aqueous solution in comparison to the 5-substituted 1-methyl-2-thiouracils reported previously [45]. The relative Gibbs free energies (ΔG_rel_) of the m1R5Se2Ura tautomers in aqueous solution were compared with those for R5Ura and R5S2Ura and are given in Table S8. These data indicated no significant difference in relative free energies of tautomers and, consequently, in tautomer equilibria between 2-thio- and 2-seleno-uracils where R5 = H or CH_2_NHCH_3_. The zwitterionic forms of mnm5X2Ura were relatively stable in solution with ΔG_rel_ values of 6.3, 4.8, and 5.9 kcal/mol, respectively, for X = O, S, and Se, as the charge was stabilized by strong interactions with a polar solvent. The difference between experimental and theoretical results seems to come mainly from the fact that variation of pH is not regarded in calculations. Experimental measurements performed in various pH showed that the p*K*_a_ values of **1**–**3** and **5**–**9** are strongly pH-dependent. Since the theoretical model did not take into account the pH effect on tautomer equilibrium, it was rather expected that the diketo-tautomers of U/S2U/Se2U nucleobases were the most stable in the conditions assessed for calculations.

#### 2.4.3. Enthalpies of Base Complexation (Pairing)

In search of energetically favoured structures displaying base pairing between the R5Se2U nucleosides and guanosine, the enthalpies of hydrogen-bonded complex formation by the most stable **K**, **E4,** and **ZI** tautomers of 1-methyl 5-substituted 2-selenouracils (m1R5Se2Ura, where R5 = H or mnm) with 9-methyl guanine (m9Gua) (taken in the most stable 6-keto form [66,67]) were calculated (Figure 4). The interaction enthalpies at 25 °C (ΔH^298^) were reported relative to those of the fully optimized isolated bases in solution (Table 5) [68]. For a given base pair, ΔH^298^ was calculated according to the following equation: ΔH^298^ = H^298^(**U**−**G**) − (H^298^(**U**) + H^298^(**G**)) + BSSE, where H^298^(**U–G**) is the enthalpy of the optimized **U–G** base pair and H^298^(**U**) and H^298^(**G**) are the enthalpies of the isolated and optimized **U** and **G** bases used in these studies, that is, **U** = m1R5Ura/m1S2Ura and **G** = m9Gua, in their most stable (canonical) tautomeric forms. Thus, for U_E2_-G, U_E4_-G, and U_ZI_-G complexes, the given ΔH values include also the enthalpy of pre-structurization of the most stable corresponding **K** tautomer into the higher energy **E2**, **E4**, or **ZI** forms. This is the same procedure as that applied in our previous paper [45], allowing the direct comparison of the stabilities of various complexes of particular uracil derivatives with 9-methyl-guanine. The deformation enthalpy (i.e., the enthalpy required to adjust the isolated and relaxed bases to the geometry of the base pair) was ignored in these calculations. However, for most of the base pairs, the optimization led to structures that were fairly close to planar. Due to base stacking and steric reasons, the base pairs in the duplexes were probably forced to adopt a more planar arrangement, thereby resulting in additional reduction of the interaction energy [69].

Comparison of bonding enthalpy orders shows that in the case of 2-oxo- and 2-thio-models the U_K_–G complex was the preferred binding mode. For the Se2 model, the enthalpies of wobble and C–G-like binding modes were almost exactly the same as for S2 model, but the “new wobble” bonding was significantly stronger than in the S2 case. Even taking into account the inaccuracy of the computational method, this difference strongly suggested that this was the preferred mode of binding of zwitterionic mnm5Se2Ura.

#### 2.4.4. Atomic Charge Distribution (ESP, Merz–Kollman Scheme) in Water

Electrostatic potential-derived (ESP) atomic charges were fitted to the electrostatic potential at points selected according to the Merz–Singh–Kollman scheme [70]. ESP charges located on the C2 and X (X = O, S, Se) atoms are displayed in Table S7, while the calculated atomic charge distributions for all tautomers of m1Se2Ura and m1mnm5Se2Ura are shown in Figure S30. Table S7 shows that the trends in variations of atomic charges between tautomers were the same in all X2Ura nucleobases (X = O, S, Se). The largest C2-X bond polarization occurred obviously in the zwitterionic form, whereas the least polarization was observed in the C2-Se bond in the E2 tautomer. Moreover, E2 tautomers of R5Se2Ura were the only case where selenium bore a positive charge. The largest change in charge upon transformation of the **K** form to the **ZI** form occurred in nitrogen, but all nucleophilic heteroatoms increased their negative charges considerably.

**Figure 5:**
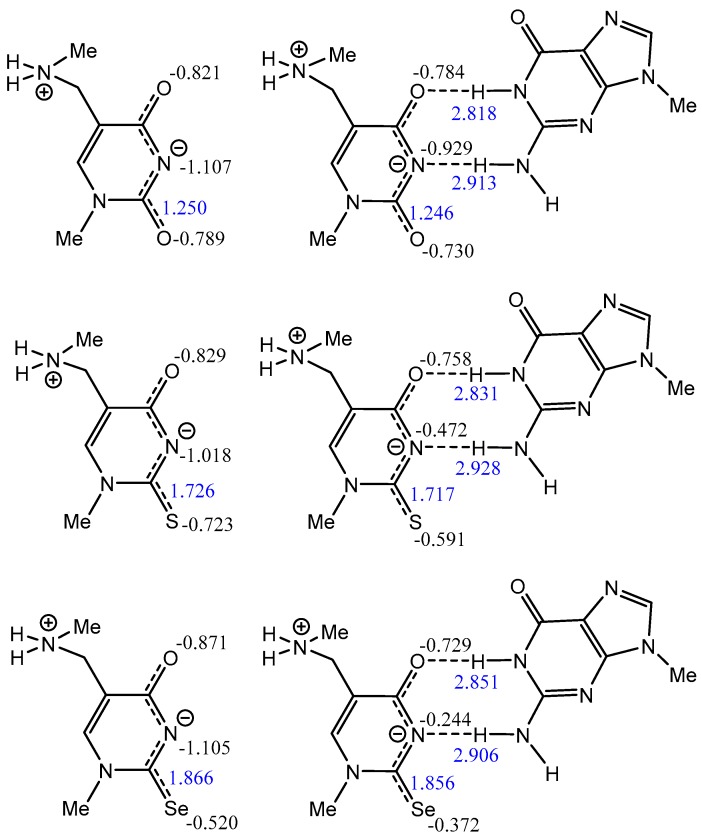
Comparison of selected electrostatic potential-derived (ESP) atomic charges and distances in zwitterionic m1mnm5Ura, m1mnm5S2Ura, and m1mnm5Se2Ura and in their complexes with 9-methyl guanine (m9Gua) in water; bond distances are marked in blue. The data were taken from Figure S30 and ref. [45].

**Figure 6:**
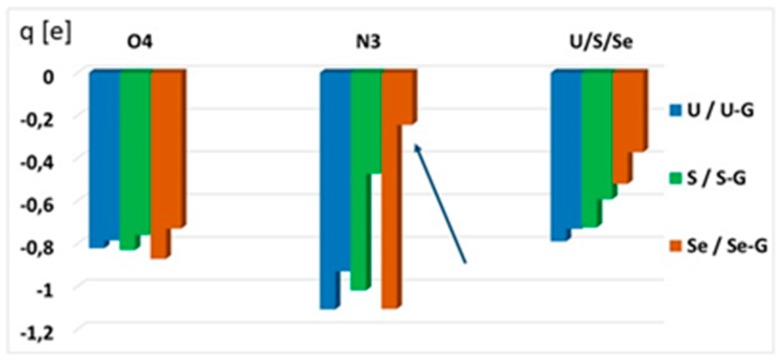
Comparison of the selected atomic charges on O4, N3, and X2 atoms in zwitterionic structures of m1mnm5Ura (blue graph), -S2Ura (green graph), and -Se2Ura (brown graph) and in their complexes with m9Gua in water (as shown in Figure 5). Each graph represents the charge value at the nucleobase in a free state (left bar) and upon its binding to m9Gua (right bar). The arrow indicates the most pronounced charge transfer observed at the N3 atom of m1mnm5Se2U upon binding to m9Gua; this feature distinguishes 2-Se uridines from their 2-oxo and 2-thio precursors.

To understand the differences between the selenium, sulfur, and oxygen models, the changes in ESP atomic charges on the O4, N3, and X2 atoms in the X2-C2-N3-C4-O4 bonding region of the zwitterionic m1mnm5X2Ura (X = O, S, Se) were analyzed before and upon formation of the base pair with m9Gua [45]. The corresponding charge distributions on the free and bound forms of 2-oxo-, 2-thio-, and 2-seleno-uracils, presented in Figure 5 (data withdrawn from Figure S30 and ref. [45]), were compared and are displayed in Figure 6. This graph shows the change in atomic charges at the O4, N3, and chalcogen atoms (in blue, green, and brown for m1mnm5Ura, m1mnm5S2Ura, and m1mnm5Se2Ura, respectively) in the form of two neighboring bars, where the left bar represents the charge before and the right bar represents the charge after the binding to the G partner. While the changes in negative charge on O4 and the chalcogen atom increased gradually upon binding, a dramatic charge transfer was observed at the nitrogen atom of the selenium-modified base (indicated by an arrow), much bigger (Δq = 0.861e) than those observed in the sulfur-modified base (Δq = 0.546e) and in the uracil base (Δq = 0.178e). Moreover, the length of the hydrogen bond N3…HN2 in the models of Se2U-G base pair was shorter than the same hydrogen bond in the corresponding S2U–G and U–G base pairs (Figure 5).

The X2U→G charge transfer in the base pair presents additional illustration of the differences in electron density. The charge transfer (Δq) from the donor (zwitterionic m1mnm5X2Ura) to the acceptor (m9Gua) was calculated as the difference between the sum of ESP atomic charges in a given molecular fragment in the complex and the charge of the corresponding isolated molecule of nucleobase (which is zero). The charge transfer values in X2U–G, where X = O, S, or Se, are given in Table 6. It was evident that the biggest charge transfer to m9Gua occurred upon binding of Se2U base.

Electrostatic potential energy maps illustrating the charge distributions in the mnm5Se2U base analyzed by quantum chemical calculations were drawn up for three the most stable tautomeric forms of the m1mnm5Se2Ura model, i.e., **K**, **E4**, and **E2**, protonated at the amino alkyl residue; these are shown in Figure S31. The data presented for tautomers of m1mnm5Se2Ura were similar to those presented for m1mnm5S2Ura and m1mnm5Ura [25], demonstrating that in the zwitterionic tautomeric structure, the electron-deficient region is located in the vicinity of the ammonium cation at the side chain, while the electron-rich region is dispersed over the Se2…N3…O4 edge. The electrostatic potential maps obtained for the three tautomeric forms of the 2-selenouracil model were consistent with those of the corresponding 2-oxo- and 2-thio-uracils [45].

## 3. Discussion

Modified nucleosides present in tRNA play a role in epitranscriptomic regulation of gene expression and allow for more accurate tuning of the translation process by restricting, expanding, or altering the decoding properties of the tRNAs. The levels of these molecules are not constant and changes in their abundance allow cells to adjust themselves in a highly dynamic manner to alterations in environmental factors, including different kinds of stress [20,71]. 5-Substituted uridines (R5Us), 2-thiouridines (R5S2Us), and 2-selenouridines (R5Se2Us) are exclusively present in a wobble position of anticodons (position 34) of tRNA specific for Glu, Gln, and Lys. Experimental and theoretical studies confirmed the regulatory function of wobble R5S2Us, which is exerted by their ability to read purine units (A and G) at the 3′-ends of the mRNA synonymous codons [20,45,72,73]. This situation is seen in all domains of life; in bacteria, the C_34_-sparing strategy (the lack of tRNA with C_34_) requires the presence of U_34_-modified tRNA^Glu^ and tRNA^Lys^ to be able to read the 3′-G-ending codon in addition to its own 3′-A-ending codon [3].

**Figure 7:**
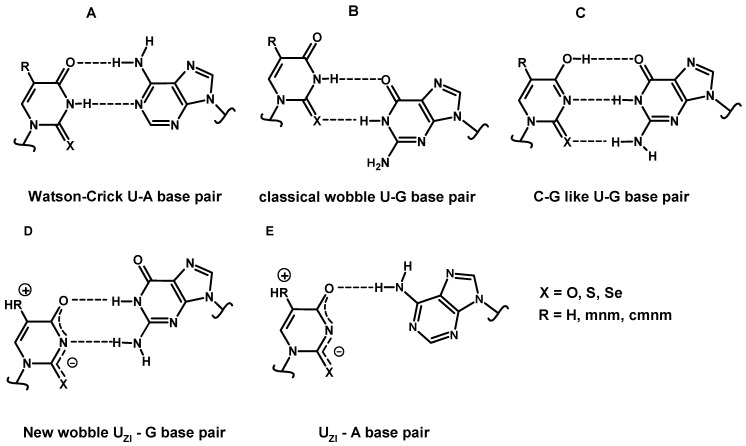
Possible base-pairing schemes for 5-substituted X2U complexed with A or G.

The combination of a sulfur atom in position 2 and an electron-withdrawing or electron-donating side chain in position 5 promotes various tautomeric forms of uracil, i.e., a 2,4-diketo form (**K** in Figure 7A,B), an **E4** tautomer (Figure 7C), or a zwitterionic **Z**I form (Figure 7D). This “chameleon” ability explains how R5S2U hybridizes with an A complement (the **K** form) and a G complement (**K**, **E4,** or **ZI** forms) [44]. The latter modes (**E4** and **ZI**) were previously demonstrated in the crystal structures of mcm5S2U_34_-tRNA (U_E4_-G) and mnm5S2U-tRNA (U_ZI_-G) bound to mRNA models accommodated at the ribosome [41,43], and were confirmed by physicochemical and theoretical studies [45]. In RNA duplexes, R5U/R5S2U can also recognize G units through the wobble mode (Crick hypothesis, Figure 7B) [74], although this spatial arrangement is not permitted by the ribosomal architecture [75].

Since R5S2Us offer at least four hybridization options, two questions arise: (1) Why did Nature develop a complex enzymatic system in bacteria (SelU, SelD, and the enzymes involved in synthesis of the geranyl component) which converts the corresponding thio-precursors **5** and **6** into 2-Se-modifications **1** and **2** in the *iso*-acceptor tRNAs decoding synonymous 5′-NNA-3′ and 5′-NNG-3′ codons? (2) What are the functions of R5S2Us, which are “safely” disabled due to this conversion that is apparently elicited ad hoc [25]? To have the tools necessary to answer these questions, we synthesized Se2-uridines **1–3** and compared their physicochemical and structural properties to the S2-uridine precursors **5–7**, as well to the uridine parent units **8–10**.

UV measurements performed for **1–3** revealed a shift of the bands at λ_max_ ca. 220 nm to 240 nm over a pH range of 6.5–7.0; this effect was slightly weaker for **3**. In the spectra recorded for **5** and **6,** the bands at 240 nm were remarkably less intense and were noted only at higher pH levels (7.5–8.0), whereas for **7,** only a weak shoulder was seen. We previously reported that bands around 240 nm may be attributed to a conjugated C2=N3-C4=O4 π-bond system present in 4-pyrimidinone-like scaffolds, e.g., in *S*-geranylated-2-thiouridine (λ_max_ ca. 240 nm) [76], 4-pyrimidinone riboside (λ_max_ ca. 247 nm for mnm5H2U) [77], and 5-methyl-4-pyrimidinone 2′-deoxyriboside (λ_max_ ca. 244 nm) [78]. Thus, the observed pH-dependent UV-shift suggested the existence of a pre-structurized tautomeric form of (c)mnm5–2-selenouridine after a transition from their classical 2,4-diketo- to the N3-deprotonated **ZI**-form (Figure 7).

**Figure 8:**
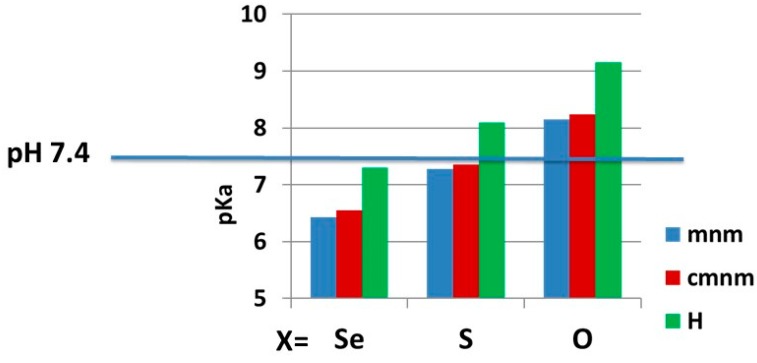
The p*K*a values of the N3H function in R5X2U wobble modification X = Se, S, or O. The units exhibiting the p*K*a values below 7.4 at physiological pH values preferentially adopt the **ZI** tautomeric form and form base pairs more efficiently with the guanosine complement, while those with p*K*a values above 7.4 preferentially adopt the **K** form and preferentially form base pairs with the A partner. Nucleosides exhibiting p*K*a values around 7.4 can adopt both tautomeric forms (**K** and **ZI**) and pair with both A and G partners.

This interpretation was supported by pH-dependent potentiometric measurements, which showed that for R5Se2U bearing an mnm, cmnm, or H substituent at C5, the p*K*a values for the processes of departure of protons from the N3-H systems (e.g., p*K*a 6.43 for the most abundant compound **1**, Table 2, Figure 8) were significantly lower compared to the corresponding S2-uridines (p*K*a 7.28 for **5**) and uridines (p*K*a 8.15 for **8**). This phenomenon originated from higher polarizability of the selenium atom compared to the sulfur or oxygen atoms [79,80], and a Δp*K*a of 3–4 units was noted earlier for selenolates (R-SeH) compared with thiolates (R-SH) [80,81,82]. The mnm and cmnm substituents significantly increased the acidity of the N3 hydrogen atoms in **1** and **2**, respectively compared to 5-unsubstituted **3** (p*K*a 6.43 or 6.55 versus 7.30, respectively), because at the physiological pH (7.4) the amino alkyl groups were substantially protonated and both substituents acquired a strong electron-withdrawing character. Consequently, at physiological pH the nucleosides **1** and **2** predominantly adopted the zwitterionic form (**ZI**) (ca. 90%, Table 2, data given in brackets; Figure 5 and Figure 7D) and, similarly to geS2U [83] and H2U units [77], their hybridization with A units might be disfavored (Figure 7E). The calculated **ZI** content for 5-nonsubstituted **3** was substantially lower (ca. 58%), yet similar to that for mnm5S2U (**5**, 57%), and much higher than for 2-thiouridine (**7**, 15%). Thus, at pH 7.4, mnm5Se2U and cmnm5Se2U are the most prone to **K**↔**ZI** pre-structurization, while U, mnm5U, cmnm5U and S2U exist mostly in the diketo-form. Remaining mnm5S2U, cmnm5S2U, and Se2U with pKa 7.0–7.5 may preferentially adopt **K** and **ZI** tautomeric forms, depending on even small changes of intracellular conditions.

We hypothesize that the prevailing content of **ZI** forms of **1** and **2** ensure the recognition of the 3′-G-ended mRNA codons and that these Se2U-G base pairings are stronger than the S2U-G pairings delivered by **5** and **6**; this was previously confirmed by functional studies [47]. Similarly, **5** and **6**, besides improved reading of A, can also read G units [45]. C5-Modified Us recognize 5′-NNA-3′ codons well, according to Watson–Crick base pairing. This “ranking” (order of appearance) is in accordance with our earlier observations, demonstrating that U_ZI_-G base pairing decreases in the order Se2U > S2U > U. However, the exceptions are wobble uridine τm5U and 2-thiouridine τm5S2U (containing a taurinomethyl C5-side chain), which exhibit p*K*a values of 7.51 and 7.10, respectively, i.e., much below the values for modified uridines **8** and **9**, but similar to those found for **5** and **6**. These taurine modifications are supposed to allow for the reading of both A and G units in synonymous mRNA codons, while their absence causes discriminate reading of G-ended codons and results in mitochondrial genetic disorders called MEERF and MELAS [71,73]. 

It is worth noting that in the biological context, the X2U34 modification (X=S, Se) has no any bias toward the 5′-NNA-3′ codon reading executed according to the classical Watson–Crick mode of interactions (Figure 7A) [3]. On the other side, the *in vitro* translation experiments carried out on globin mRNA have shown that selenium-modified tRNA^Glu^ and tRNA^Lys^ exert a stronger preference for reading the NNG over NNA codons, compared to their thio-precursors [47]. Thus, these aforementioned biological data are compatible with our p*K*a measurements, which indicate the preferred involvement of the zwitterionic R5Se2U in base pairing with guanosine (Figure 7D and Figure 8).The effects of substitution of the O2 oxygen atom with selenium in the uracil molecule were also investigated by DFT calculations performed on m1R5X2Ura-m9Gua models (R = H or mnm, X = O, S, or Se, Figures S27–S31, Tables S7–S9). Although the atomic radius of Se and the length of the C2-Se bond were larger than those for sulfur, the enthalpies of formation for the m1Se2Ura-m9Gua and m1S2Ura-m9Gua base pairs (R=H) were found to be only a little different from each other (ΔH^298^ of -7.9 vs -8.1 kcal/mol for “wobble” U_K_-G, -6.2 vs -6.6 kcal/mol for “C–G-like” U_E4_-G, respectively) (Figure 4, Table 5). Only in the “new wobble” base pairing, in which the selenium atom does not participate directly, the difference between m1mnmSe2U_ZI_-m9G and m1mnmS2U_ZI_-m9G was higher (ΔH^298^ -8.6 vs -7.3 kcal/mol, respectively), thereby providing energy-based evidence that the presence of selenium in the “wobble” nucleosides makes a difference. This difference in enthalpy was most likely related to the greater polarizability of the selenium atom compared to the sulfur atom [80].

Important data were obtained from calculations of changes in electron density at the atoms in the X-C2-N3-C4-O4 bonding region of the zwitterionic form of m1mnm5X2Ura (X = O, S, or Se) upon binding with m9Gua. The changes in the O4 oxygen and X2 atoms were small; the biggest effect was found for the Se congener. The most pronounced change was noted at the N3 atom of m1mnm5Se2Ura (Δq = 0.861e, indicated by an arrow at Figure 6), while those in the S2 and O2 congeners were ca. 60% and 20% of this value, respectively (Δq = 0.546e and 0.178e). As a consequence, the predicted length of the (Se2U)N3–H-N2(G) hydrogen bond was the shortest compared to the base pairs bearing the S2 or O2 congeners (Figure 5). The energy of hydrogen bonds results is determined by steric, electrostatic, covalent, and dispersion interactions [84,85]. The charge transfer in a complex utilizing hydrogen bonds is mainly associated with the covalent (i.e., orbital interaction) contribution to the H-bond and its increase on the X=O to X=Se switch is in accord with increasing bonding energy. In respect to the overall charge transfer from m1R5X2Ura to m9Gua, the biggest difference was noted for m1mnm5Se2Ura, followed by the S2 and O2 congeners (Table 6, Δq = 0.243, 0.170, and 0.093 e, respectively), suggesting that the greater polarizability of the selenium atom facilitated the transfer of electron density to guanine, resulting in stronger hydrogen bonding in the resultant base pair. The so called “new wobble” base pairing (U_ZI_-G) was remarkably stronger in the **ZI** form than the 2-oxo- and 2-thio-analogs (ΔΔH = −2.7 and −1.3 kcal/mol, respectively, Table 5).

The molecular structure of **3** was determined by X-ray diffraction (Figure 3) and compared to the structures of U and S2U (Table 3, Tables S1–S6). For all three nucleosides, the expected N-type sugar ring puckering was confirmed [63] and the lengths of the C2-X bonds were determined at 1.227 Å for uridine [60], 1.677 Å for S2U (structure A [59]), and 1.851(8) Å for Se2U. The last value was remarkably different from those reported for compounds bearing a single C-Se bond (1.94 Å) and double C-Se bond in selenones (1.74 Å) [61]. The latter length was also exhibited by the C2-Se bond in the Se2U-A base pair (present in an RNA duplex), indicating the presence of a diketo-tautomer [29]. Se2U most likely adopted a tautomeric structure of a hybrid of 2,4-diketo- and 4-keto-2-enol forms, analogous to that of 1-mesitylimidazole-2-selone possessing a tautomerizable HN-C-Se system, for which the length of the C-Se bond was assessed as 1.845(2) Å [61]. The C-Se distance found in the crystal structure and that obtained by our DFT calculations in H_2_O (Figure S27) differed by only 0.03 Å, enhancing our confidence in the presented calculations, especially with regard to the larger value found in the crystal, which may be attributed to the intermolecular forces present in the crystal lattice.

The literature data [35] and the results of ^1^H NMR measurements performed for **1–3** indicated that in solution, both S2 and Se2 modifications made the sugar C3′-*endo* conformation more profound compared to O2 congeners (Table 4), and this feature was usually explained by steric repulsion between a relatively large S/Se atom and an O2′ atom [86]. However, replacement of the sulfur atom with a larger selenium atom did not make the vicinity of the 2′-OH group in **1** and **2** more crowded (the differences were virtually negligible for **1**/**5** and **2**/**6**), indicating that the selenium atom operated in nucleosides conformationally similar to the S2 analogues. The population of the C3′-*endo* form (80%) was undoubtedly higher in 2-selenouridine **3** than in 2-thiouridine **7** (71%), which may be a reason for the increased stability of the RNA duplexes containing the Se2U-A base pair [29].

## 4. Materials and Methods

### 4.1. Chemistry

Detailed original procedures of the synthesis of 5-methylaminomethyl-2-selenouridine (mnm5Se2U, **1**) and 5-carboxymethylaminomethyl-2-selenouridine (cmnm5Se2U, **2**), as well as of 2-selenouridine (Se2U, **3**) and ^1^H and ^13^C spectra of **1**, **2**, **3** (for the latter also ^77^Se NMR spectrum) and their intermediates **1c**–**e**, **2c**–**e** and **3b**–**c** (Figures S1–S25), are given in Supplementary Materials. 2-Seleno-uridines **1**, **2,** and **3** were obtained from their respective 2-thiouridine precursors **1b**, **2b,** and **3a** in several-step procedures with 31%, 38%, and 57% total yields, respectively.

### 4.2. Physicochemical Studies

#### 4.2.1. The UV Measurements and Extinction Coefficients of **1**, **2,** and **3**

UV spectra were recorded on a Specord^®^ 50 plus spectrophotometer (Analytik, Jena, Germany). Samples were prepared by dilution of 4 μL of nucleoside stock solution (ca 1 mg of nucleoside in 1 mL water) in 996 μL of buffer solution (10 mM HCl at pH 3.0, 67 mM Na_2_HPO_4_/KH_2_PO_4_ buffer at pH 5.0, 6.0, 6.5, 7.0, 7.5, 8.0). To determine molar extinction coefficients (ε) of **1–3**, nucleosides of known weight were dissolved in 50 mL of water of pH 6.5, then 1 mL of solution was transferred to a 1 mL quartz cell and placed in a Specord^®^ 50 plus spectrophotometer. The UV spectra were collected for three independent samples and the extinction coefficients (a mean value) were calculated from the Beer–Lambert equation using absorbance values determined for λ_max_ and 260 nm.

#### 4.2.2. Potentiometric Measurements

The acidity constants of the nucleosides **1**–**3** (p*K*a) were determined by the pH-potentiometric titration as described previously [45]. pH-dependent p*K*a determination by UV measurements is shown in Figure S26.

### 4.3. Crystallographic Analysis

The crystals of 2-selenouridine **3** were obtained from ethanol solution in the form of thin rhombic plates, creating clusters. The plates were hard to separate and very easily cracked, therefore, the MicroMeshes loop from MiTeGen was used to separate, fish, and mount the crystal on the goniometer head. The diffraction data of 2-selenouridine crystal were collected at 100 K using an Oxford SuperNova diffractometer with micro-source Cu Kα radiation (λ = 1.54 Å) with a Titan detector. Diffraction data collection, cell refinement, and data reduction were performed using the *CrysAlis PRO* program (Oxford Diffraction). The structure was solved by direct SHELXS methods implemented into the *OLEX2* package [87] and refined using full-matrix least-squares difference Fourier techniques *SHELX97*. Crystallographic data for **3** was deposited into the Cambridge Structural Database under accession number CCDC 1850614. The asymmetric unit contained one molecule of 2-selenouridine. The carbon and nitrogen hydrogen atoms were set geometrically and refined as riding and the hydrogen atoms connected with oxygen were found on the difference Fourier map and refined with geometrical restrains. The anisotropic thermal parameters were applied for all nonhydrogen atoms and isotropic parameters for hydrogens, equal to 1.2 of the thermal parameters of their parental atoms for geometrically restrained ones. The absolute configuration of carbon atoms of the sugar ring was determined based on the known ribose ring and confirmed by the Flack parameter -0.025(48) (classical fit to all intensities) and -0.045(27) from 850 selected quotients (Parsons’ method).

### 4.4. Quantum Mechanical Calculations

DFT calculations of the relative stability of R5Se2U tautomers in water and of the binding enthalpy of R5Se2U base pairs with A and G (a model for wobble base pairing at the ribosome) were performed using Gaussian 16 suite of programs [88] using procedures analogous to those described in the previous paper concerning sulfur derivatives, R5S2U [45]. Geometries of the nucleic bases and base pair model systems were optimized using the hybrid B3LYP density function [89], which was corrected for dispersion interactions using the Grimme GD3 empirical term [90,91] and the 6–31+G(d) basis set in aqueous solution. All stationary points were identified as stable minima by frequency calculations. Thermochemical corrections were scaled by a factor of 0.98. More accurate electronic energies were obtained using the B3LYP functional, including the Grimme GD3 dispersion correction with the larger 6311++G(3df,2p) basis set. Calculations in solution were performed within the Conductor-like Polarizable Continuum Model (CPCM), assuming Universal Force Field (UFF) cavities [92]. Free energy of solvation was estimated using the CPCM procedure for consistency with the geometry calculations and to allow direct comparison with the previous study [45]. Atomic charges were calculated according to the Merz–Kollman scheme [70], using a covalent radius of 1.20 Å for selenium [93]. The basis set superposition errors (BSSE) for the complex formation were corrected using the counterpoise procedure (CP) of Boys and Bernardi [94] at the B3LYP/6-311++G(3df,2p) level of theory. The values of error were in the range of 0.28–0.43 kcal/mol for all the complexes studied.

## 5. Conclusions

5-Methylaminomethyl- and 5-carboxymethylaminomethyl-2-selenouridine, identified in position 34 (wobble) of tRNA iso-acceptors specific for lysine, glutamine, and glutamic acid, were synthesized and their structural and physicochemical properties were investigated. pH-dependent potentiometric measurements for the ionization of the N3H groups revealed p*K*a values lower than those for the sulfur-containing congeners, and this easier ionization most likely occurred because of higher polarizability of the selenium atom. Since at physiological pH 7.4 the amino alkyl side chains in both nucleosides were protonated (p*K*a ca. 9.0), the electron withdrawing effect promoted nucleosides’ zwitterionic tautomeric forms. The propensity of mnm5Se2U and cmnm5Se2U for tautomerization was supported by X-ray diffraction data collected for Se2U, where the length of the C-Se bond indicated a bond order of <2, as was found in a hybrid of 2,4-diketo- and 4-keto-2-enol forms. The tautomers **E4** and **ZI** effectively hybridized with guanosine to form the Se2U-G base pair according to “C–G-like” or “the new wobble mode”, and the resultant complexes were more stable than the S2U–G and U–G base pairs. This phenomenon received convincing theoretical support from DFT calculations on three m1R5X2Ura-m9Gua models. It was found that the Se2U–G pairing was characterized by a lower binding enthalpy as well as a bigger Se2U→G charge transfer compared to the S2U and U congeners. These data suggested that the tRNA anticodons with wobble R5Se2Us may preferentially read the 5′-NNG-3′ synonymous codons, unlike their 2-thio- and oxo-precursors, which preferentially read the 5′-NNA-3′ codons, and this was actually confirmed by biological experiments with seleno-U34-tRNA [47]. Thus, the interplay between the levels of U-, S2U-, and Se2U-tRNA may have a dominant role in protein expression regulation in a situation where one U*_34_-tRNA has to decode two 3′-purine-ending synonymous codons.

## Figures and Tables

**Table 1:** UV absorption coefficients and absorbance maxima of 2-selenouridines **1–3** and 2-thiouridines **5–7**.

Comp. Number	Abbreviation Name	λ_max_ (nm)			ε^1^ (dm^3^·mol^−1^·cm^−1^)
		pH 3	pH 7	pH 8	
**1**	mnm5Se2U	223, 314	231,302	240, 298	12669 (303 nm), 6901 (260 nm)
**2**	cmnm5Se2U	223, 314	231,302	240, 298	12695 (310 nm), 4951 (260 nm)
**3**	Se2U	223, 309	225,306	240, 295	13992 (308 nm), 2687 (260 nm)
**5**	mnm5S2U	219, 274	221,275	240, 276	10411 (276 nm), 7283 (260 nm)
**6**	cmnm5S2U	221, 275	221,275	223, 240, 277	12104 (276 nm), 7645 (260 nm)
**7**	S2U	217, 276	220,276	221, 240, 274	14410 (276 nm), 9160 (260 nm)

^1^ Samples were dissolved in distilled water at pH 6.5.

**Table 2:** The p*K*a values of selenonucleosides **1–3** determined by pH-dependent potentiometric titration. The p*K*a values (determined at 25 °C) were assessed for the dissociation of the N3H proton and for the protonation/deprotonation of the amino alkyl and carboxyl groups present in the C5 side chains (±SD = 0.01). The content (%) of fractions of nucleosides ionized at N3H at pH 7.4 was calculated according to the Henderson–Hasselbalch equation and is given in brackets. The p*K*a data for 2-thio and 2-oxo analogues of **1–3** are cited for comparison [45].

R (Abbreviated Name of the Substituent)	Donor/Acceptor	Nucleoside^1^		
		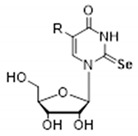	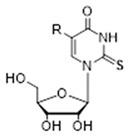	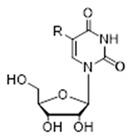
CH_3_NHCH_2_ (mnm)	N3H	6.43 (>90, *78*^2^)	7.28 (57, *34*^2^)	8.15 (15)
	NHCH_2_	9.36	9.51	10.02
HOOC-CH_2_NHCH_2_ (cmnm)	N3H	6.55 (89, *74*^2^)	7.36 (52, *30*^2^)	8.24 (13)
	NHCH_2_	8.89	9.10	10.13
	COOH	2.26	2.50	3.05
H	N3H	7.30^3^ (58, *34*^2^)	8.09 (17)	9.15 (2)

^1^ The p*K*a values and the contents of the ionization fractions (in brackets) for 5-substituted 2-thiouridines and uridines were taken from ref. [45]. ^2^ The content of ionized fractions of 5′-phosphates of 2-seleno- and 2-thio-nucleosides, recalculated based on the p*K*a value increased by an arbitrary 0.4 units, are given in italics. ^3^ p*K*a 7.29 for Se2U was determined by Sun et al. [29].

**Table 3:** Conformational crystallographic data for **Se2U** in comparison with 2-thiouridine (**S2U**) [59] and uridine (**U**) [60].

Conformational Parameters	Se2U (1)	S2U (7)	U (10)	
			A	B
P [^o^]	**14.4 (N)**	9.7 (N)	3.8 (N)	13.9 (N)
ψ_m_ [^o^]	**34.7**	36.5	39.6	41.6
Sugar moiety	***C3′-endo***	*C3′-endo*	*C3′-endo* *C2′-exo*	*C3′-endo*
C4′-C5′ bond	***trans***	*trans*	*gauche* *(+)*	*gauche* *(+)*
C1′-N1 bond	***anti***	*anti*	*anti*	*anti*

**Table 4:** The sugar ring conformation in the series of R5-substituted X2-uridines (where R = H, mnm, cmnm and X = O, S, Se), as determined by ^1^H NMR measurements. The C3′-*endo* content values (in %) were calculated according to the equation C3′-*endo* (%) = (10-J_H1′,H2′_)/10 × 100.

Compound Number	Abbreviation Name	C3′-*endo* (%)
**1**	mnm5Se2U	72
**2**	cmnm5Se2U	79
**3**	Se2U	80
**5**	mnm5S2U	76 ^1^
**6**	cmnm5S2U	82 ^1^
**7**	S2U	71 ^1^
**8**	mnm5U	57 ^1^
**9**	cmnm5U	58 ^1^
**10**	U	53 ^1^

^1^ These values were taken from ref. [45].

**Table 5:** Enthalpies of formation of the complexes of 9-methyl guanine (m9Gua) and modified uracils (m1R5X2Ura, where X = O, S, Se) in water calculated using the CPCM-B3LYP-GD3/6-311++G(3df,2p)//B3LYP/6-31+G(d) method (in kcal/mol). The corresponding structures of the base pairs are shown in Figure 4.

Base Pair Mode	ΔH^298^ of a Base Pair of m9Gua with m1R5X2Ura Component (kcal/mol)						
	R5	H			mnm		
	X	O^1^	S^1^	Se	O^1^	S^1^	Se
**U_K_-G** (wobble)		−10.0	−8.1	−7.9	−10.2	−8.4	−8.4
**U_E4_-G** (C–U-TG-like)		−7.8	−6.6	−6.2	−7.6	−6.4	−6.8
**U_ZI_-G** (new wobble)		-	-	-	−5.9	−7.3	−8.6

^1^ data taken from ref. [45].

**Table 6:** Charge transfer mnm5X2U(ZI)→G in the base pair and the difference in charge transfer (Δq) in particular base pairs.

X	O	S	Se
Charge transfer [e]	0.093	0.170	0.243
Δq = q_X_-q_O_ [e]	0	0.077	0.150 (q_Se_-q_O_) 0.073 (q_Se_-q_S_)

## Supplementary Materials

The following are available online at https://www.mdpi.com/1422-0067/21/8/2882/s1, Methods: Synthetic procedures of **1**-**3**, protocol for potentiometric measurements. Spectral analysis of Se2U derivatives: Figures S1–S8 - ^1^H and ^13^C spectra of mnm5Se2U derivatives, Figures S9–S16 - ^1^H and ^13^C spectra of cmnm5Se2U derivatives, Figures S17–S25 - ^1^H, ^13^C, ^77^Se spectra of Se2U derivatives; Figure S26 - pH-dependent pKa determination by UV measurements, Figures S27–S31 - Figures of DFT calculations, Tables S1–S6 – Data for crystal structure of Se2U, Tables S7–S9 - Data of DFT calculations.
