# Supplementary material for: C5-Substituted 2-Selenouridines Ensure Efficient Base Pairing with Guanosine; Consequences for Reading the NNG-3′ Synonymous mRNA Codons
Source: Int J Mol Sci. 2020 Apr 20;21(8):2882. doi: 10.3390/ijms21082882 (PMC7216251; doi:10.3390/ijms21082882)
Supplement: Supplementary file 1 [file ijms-21-02882-s001.pdf]

Supplementary Material

C5-Substituted 2-selenouridines ensure efficient base pairing with guanosine; consequences for reading the NNG-3’ synonymous mRNA codons

Grazyna Leszczynska[1], Marek Cypryk[2], Bartlomiej Gostynski[2], Klaudia Sadowska[1], Paulina Herman[1], Grzegorz Bujacz[3], Elzbieta Lodyga-Chruscinska[3], Elzbieta Sochacka[1] and Barbara Nawrot[2,*]

[1] Institute of Organic Chemistry, Faculty of Chemistry, Technical University of Lodz, Zeromskiego 116, 90-924 Lodz, Poland
[2] Centre of Molecular and Macromolecular Studies, Polish Academy of Sciences, Sienkiewicza 112, 90-363 Lodz; Poland
[3] Institute of Molecular and Industrial Biotechnology, Lodz University of Technology, 4/10 Stefanowskiego St., 90-924 Lodz, Poland

Table of contents:

1. Chemistry
    a. General remarks
    b. Synthesis of 5-methylaminomethyl-2-selenouridine (mnm5Se2U, **1**)
    c. Synthesis of 5-carboxymethylaminomethyl-2-selenouridine (cmnm5Se2U, **2**)
    d. Protocol for preparation of 2-selenouridine (Se2U, **3**)
    e. Potentiometric measurements
2. Spectral analysis of Se2U derivatives
    a. ^{1}H and ^{13}C spectra of mnm5Se2U derivatives (Figures S1-S8)
    b. ^{1}H and ^{13}C spectra of cmnm5Se2U derivatives (Figures S9-S16)
    c. ^{1}H, ^{13}C, ^{77}Se spectra of Se2U derivatives (Figures S17-S25)
    d. pH-dependent pKa determination by UV measurements (Figure S26)
3. Crystal structure of Se2U (Tables S1-S6)
4. Results of DFT calculations (Figures S27-S31, Tables S7-S9)

1. **Chemistry**

General Remarks

Thin layer chromatography was done on silica gel coated plates (60F254, Merck), and Merck silica gel 60 (mesh 230–400, Merck) was used for column chromatography. HPLC was performed with a Waters chromatograph equipped with a 996 spectral diode array detector preparative SUPELCO, Ascentis® column (C18, 25 cm x 21.2 mm, 10 µm). Separation was run at room temperature (rt) using water as an eluent. NMR spectra were recorded at a 700 MHz (for ^{1}H) instrument and at 176 MHz for ^{13}C. Chemical shifts (δ) are reported in ppm relative to TMS (an internal standard) for ^{1}H and ^{13}C. The signal multiplicities are described as s (singlet), d (doublet), dd (doublet of doublets), t (triplet), q (quartet), m (multiplet), and bs (broad singlet). High-resolution mass spectrometry (HRMS) measurements were performed using Synapt G2Si mass spectrometer (Waters) equipped with an ESI source and quadrupole-Time-of-flight mass analyser or using a Finnigan MAT 95 spectrometer (FAB ionization).

Synthesis of 5-methylaminomethyl-2-selenouridine (mnm5Se2U, 1)

*5'-O-(4,4'-Dimethoxytrityl)-5-(N-trifluoroacetyl)methylaminometyl-S-methyl-2-thiouridine (**1c**).* To the solution of 2-thiouridine **1b** (144 mg, 0.35 mmol) in anhydrous EtOH (3 ml), triethylamine (153 µl, 1.1 mmol) and methyl iodide (68 µl, 1.1 mmol) were added. The mixture was stirred for 2 h at room temperature. The reaction mixture was evaporated under reduced pressure and the solid residue was partitioned between dichloromethane (DCM, 6 ml) and water (3 ml). The water phase was extracted twice with DCM (2 x 6 ml). The organic layers were combined, dried over anhydrous $MgSO_4$ and evaporated under reduced pressure. The residue was co-evaporated with anhydrous toluene and purified on column of silica gel with 2% methanol in DCM. Pure **1c** was obtained in 90 % yield (134 mg). TLC ($CHCl_3$:MeOH, 95:5, v/v) R_f = 0.32; ^{1}H NMR (700 MHz, $CDCl_3$) δ (ppm): 2.56 (s, 3H, -SCH3), 3.20 (s, 3H, N-CH_3); 3.42 (dd, 1H, ^{3}J=3.5 Hz, ^{2}J=11.2 Hz, H5"), 3.54 (dd, 1H, ^{3}J=3.5 Hz, ^{2}J=11.2 Hz, H5'), 3.70 (d, 1H, ^{2}J=14.0 Hz, H-5,1), 3.77 (s, 6H, 2xOCH_3), 3.91 (d, 1H, ^{2}J=14.0 Hz, H-5,1), 4.17-4.19 (m, 1H, H4'), 4.31 (t, 1H, ^{3}J=4.9 Hz, H3'), 4.34 (t, 1H,^{3}J=4,9 Hz, H2'), 5.86 (d, 1H, ^{3}J=4.9 Hz, H1'), 6.82-6.83 (m, 4H, H_{Ar}), 7.20 -7.43 (m, 9H, H_{Ar}), 7.83 (s, 1H, H6); ^{13}C NMR (176 MHz, $CDCl_3$) δ (ppm): 15.20 (SCH_3), 37.08 (NCH_3), 47.86(CH_2N), 55.33 (2xOCH_3), 63.18 (C5'), 70.69 (C3'), 75.29 (C2'), 84.13 (C4'), 87.10 (CPh_3), 92.07 (C1'), 113.44 (C_{Ar}), 115.46 (C5), 116.20 (q, ^{1}J=288 Hz, CF_3), 127.17 (C_{Ar}), 128.13 (C_{Ar}), 128.36 (C_{Ar}), 130.36 (C_{Ar}), 135.62 (C_{Ar}), 135.66 (C_{Ar}), 140.00 (C6), 144.61 (C_{Ar}), 157.28 (q, ^{2}J=36 Hz, $COCF_3$), 158.81 (C_{Ar}), 163.64 (C2), 168.52 (C4). HRMS (FAB) calcd. for $C_{35}H_{37}F_3N_3O_8S$ $[M+H]^+$ 716.2253, found 716.2245. (Figures S1-S2).

*5'-O-(4,4'-Dimethoxytrityl)-5-(N-trifluoroacetyl)methylaminomethyl-2-selenouridine (**1d**).* Selenium (110 mg, 1.39 mmol, 10 eq.) was suspended in anhydrous ethanol (1 ml) at 0 °C and then sodium borohydride ($NaBH_4$, 80 mg, 2,08 mmol, 15 eq.) was added. The reaction was stirred for 2 h at 0 °C to get the clear solution of ethanolic NaSeH. Then *S*-methyl-2-thiouridine **1c** (100 mg, 0,14 mmol, 1 eq.) was added and the mixture was stirred for 2.5 h at room temperature. The reaction mixture was then evaporated under reduced pressure. The residue was dissolved in the mixture of ethyl acetate – water (6 ml, 1:1, v/v). The organic phase was separated and water was extracted several times with ethyl acetate (6 x 3 ml). Organic layers were combined, dried over anhydrous $MgSO_4$ and evaporated under reduced pressure. The residue was purified by flash column chromatography using argon overpressure. Pure selenouridine derivative **1d** was obtained in 60 % yield (63 mg) TLC ($CHCl_3$:MeOH, 95:5, v/v) *Rf* =0.42; ^{1}H NMR (700 MHz, $CDCl_3$) δ (ppm): 3.20 (s, 3H, NCH_3), 3.52 (dd, 1H, ^{2}J=11.2 Hz, ^{3}J=3.5 Hz, H5"), 3.54 (d, 1H, ^{2}J= 13.3 Hz, H-5,1), 3.61 (dd, 1H, ^{2}J=11.2 Hz, ^{3}J=1.4 Hz, H5'), 3.64 (d, 1H, ^{2}J= 13.3 Hz, H-5,1), 3.78 (s, 6H, 2xOCH_3), 4.23-4.25 (m, 1H, H4'), 4.27-4.29 (m, 1H, H3'), 4.41-4.46 (m, 1H, H2'), 6.45 (bs, 1H, H1'), 6.81-6.85 (m, 4H, H_{Ar}), 7.21-7.44 (m, 9H, H_{Ar}), 8.16 (s, 1H, H6); ^{13}C NMR (176 MHz, $CDCl_3$) δ (ppm): 36.21 (NCH_3); 46.46 (CH_2N), 54.36 (2xOCH_3), 61.28 (C5'), 68.75 (C3'), 75.33 (C2'), 83.60 (C4'), 86.05 (CPh_3), 95.89 (C1'), 112.47 (C_{Ar}), 113.82 (C5), 115.17 (q, ^{1}J= 287.76 Hz, CF_3), 126.17 (C_{Ar}), 127.15 (C_{Ar}), 127.37 (C_{Ar}), 127.48 (C_{Ar}), 129.44 (C_{Ar}), 134.49 (C_{Ar}), 134.1 8(C_{Ar}), 140.53 (C6), 143.77 (C_{Ar}), 156.21 (q, ^{2}J= 36.26 Hz, $COCF_3$), 157.84 (C_{Ar}), 158.43 (C4), 174.31 (C2). HRMS (ESI) calcd. for $C_{34}H_{33}F_3N_3O_8Se$ $[M-H]^+$ 748.1385, found 748.1371. (Figures S3-S4).

*5-(N-Trifluoroacetyl)methylaminomethyl-2-selenouridine (**1e**).* 5'-*O*-(4,4'-dimethoxytrityl)-5-(*N*-trifluoroacetyl)methylaminomethyl-2-selenouridine (**1d**) (73 mg, 0.1 mmol) was dissolved in 50% aq.

AcOH (4.4 ml). The solution was stirred for 1.5 h at room temperature and evaporated *in vacuo*. The residue was co-evaporated twice with anhydrous toluene and purified by flash column chromatography (8 % methanol in DCM) using argon overpressure. Pure detritylated selenouridine **1e** was obtained in 70 % yield (31 mg) as a mixture of rotamers about –NC(O)CF_3 amide bond in a 0.8 : 0.2 ratio according to ^{1}H NMR. Consequently, two chemical shifts are observed for some of the ^{1}H and ^{13}C NMR resonances (the secondary shifts in ^{13}C NMR spectra are given in parentheses). TLC ($CHCl_3$/MeOH, 95/5) R_f =0,18; TLC ($CHCl_3$/MeOH, 90/10) R_f =0.62; TLC (iPrOH/NH_3/H_2O, 7:2:1, v/v/v) R_f = 0.76; ^{1}H NMR (700 MHz, CD_3OD) δ (ppm): 3.04 (s, 0.6H, NCH_3), 3.29 (s, 2.4H, NCH_3), 3.82 (dd, 0.2H, ^2J=12.6 Hz, ^3J=2.1 Hz, H5''), 3.89 (dd, 0.8H, ^2J=12.6 Hz, ^3J=2.8 Hz, H5''), 3.96 (dd, 0.2H, ^2J=12.6 Hz, ^3J=2.1 Hz, H5'), 4.01 (dd, 0.8H, ^2J=12.6 Hz, ^3J=2.8 Hz, H5'), 4.13-4.15 (m, 1H, H4'), 4.29-4.45 (m, 4H, H2', H3', CH_2-5,1), 6.78 (d, 0.8H, ^3J=2.8 Hz, H1'), 6.79 (m, 0.2H, H1'), 8.43 (s, 0.2H, H6), 8.50 (s, 0.8H, H6); ^{13}C NMR (176 MHz, CD_3OD) δ (ppm): 36.47 (35.14)(NCH_3), 47.54 (47.16) (CH_2N), 61.34 (60.72) (C5'), 70,81 (69.93) (C3'), 77.03 (77.24) (C2'), 86.32 (C4'), 97.74 (C1'), 115.41 (115.70) (C5), 117.79 (q, ^1J=277,02 Hz, CF_3), 142.32 (139,67) (C6), 158.48 (q, ^2J=35,72Hz, $COCF_3$); 161.20 (C4), 177.66 (177.33) (C2). HRMS (ESI) calcd. for $C_{13}H_{15}F_3N_3O_6Se$ $[M-H]^+$ 446.0078, found 446.0083.(Figures S5-S6).

*5-Methylaminomethyl-2-selenouridine (**1**).* 5-(N-trifluoroacetyl)methylaminomethyl-2-selenouridine (**1e**) (20 mg, 0.05 mmol) was dissolved in 30% aq. ammonia (1.4 ml). After stirring at room temperature for 15 min the mixture was evaporated under reduced pressure. The residue was purified by flash column chromatography (20 % methanol in DCM) using argon overpressure. Compound **1** was obtained in 83 % yield (13 mg). TLC ($CHCl_3$:MeOH, 90:10, v/v) R_f =0.31; TLC (iPrOH:NH_3:H_2O, 7:2:1, v/v/v) R_f = 0.40; ^{1}H NMR (700 MHz, D_2O) δ (ppm): 2.75 (s, 3H, NCH_3), 3.92 (dd, 1H, ^2J=13.3 Hz, ^3J=3.5 Hz, H5''), 3.97 (s, 2H, CH_2-5,1), 4.07 (dd, 1H, ^2J=13.3 Hz, ^3J=2.8 Hz, H5'), 4.21-4.26 (m, 2H, H3', H4'), 4.43 (dd, 1H, ^3J=2.8 Hz, ^3J=4.9 Hz, H2'), 6.86 (d, 1H, ^3J=2.8 Hz, H1'), 8.34 (s, 1H, H6). ^{13}C NMR (176 MHz, D_2O) δ (ppm): 32.28 (NCH_3), 46.68 (CH_2N), 59.66 (C5'), 68.18 (C3'), 75.22 (C2'), 83.78 (C4'), 95.92 (C1'), 109.83 (C5), 141.67 (C6), 167.52 (C4), 175.96 (C2). HRMS (ESI); calcd. for $C_{11}H_{18}N_3O_5Se$ $[M+H]^+$ 352.0412, found 352.0398. (Figures S7-S8).

Synthesis of 5-carboxymethylaminomethyl-2-selenouridine (2)

*5'-O-(4,4'-Dimethoxytrityl)-N-[(1-β-D-ribofuranosyl-1H-2-methylthiopyrimidin-5-yl)methyl]-N-trifluoroacetylglycine 2-(trimethylsilyl)ethyl ester (**2c**).* To the solution of 2-thiouridine **2b** (200 mg, 0.23 mmol, 1 eq.) in anhydrous EtOH (2.5 ml) triethylamine (96 µl, 0.69 mmol, 3 eq.) and methyl iodide (43 µl, 0.69 mmol, 3 eq.) were added. The mixture was stirred for 2 h at room temperature. The reaction mixture was evaporated under reduced pressure and the solid residue was partitioned between DCM (8 ml) and water (4 ml). The water phase was extracted twice with DCM (2 x 8 ml). The organic layers were combined, dried over anhydrous $MgSO_4$ and evaporated under reduced pressure. The residue was co-evaporated with anhydrous toluene and purified on column of silica gel with 2% methanol in chloroform. Pure compound **2c** was obtained in 80 % yield (158 mg) as a mixture of rotamers along amide bond NC(O)CF_3 in a 0.88: 0.12 ratio according to ^{1}H NMR. Consequently, two chemical shifts are observed for some of the ^{1}H and ^{13}C NMR resonances (the secondary shifts in ^{13}C NMR spectra are given in parentheses). TLC ($CHCl_3$:MeOH, 95:5, v/v) R_f = 0.41; ^{1}H NMR (700 MHz, $CDCl_3$) δ (ppm): 0.01 (s, 1.08H, $SiMe_3$), 0.03 (s, 7.92H, $SiMe_3$), 0.95-1.0 (m, 2H, $SiCH_2$), 2.58 (s, 3H, -SCH3), 3.42-3.55 (m, 2H, H5', H5"), 3.79 (s, 6H, 2xOCH_3), 3.84 (d, 1H, ^2J=14.0 Hz, CH-5,1), 3.95 (d, 1H, ^2J=14.0 Hz, CH-5,1), 4.15-4.18 (m, 1H, H4'), 4.21-4.24 (m, 2H, CH_2OC(O)), 4.27-4.32 (m, 2H, H2', H3'), 4.54 (d, 1H, ^2J=18.9 Hz, CHC(O)O), 4.64 (d, 1H, ^2J=18.9 Hz, CHC(O)O), 5.84 (d, 0.12H, ^3J=4.9 Hz, H1'), 5.86 (d, 0.88H, ^3J=3,5 Hz, H1'), 6.81-6.86 (m, 4H, H_{Ar}), 7.19 -7.46 (m, 9H, H_{Ar}), 7.57 (s, 0.12H, H6), 7.88 (s, 0.88H, H6); ^{13}C NMR (176 MHz, $CDCl_3$) δ (ppm): -1.49 (1.16) ($SiMe_3$), 15.16 (14.25) (SCH_3), 17.46 ($SiCH_2$), 48.09 (CH_2-5.1), 51.28 (CH_2C(O)), 55.32 (2xOCH_3), 63.15 (C5'), 64.27 (CH_2OC(O)), 70.62 (71.15) (C3'), 75.46 (74.80) (C2'), 83.78 (C4'), 87.18 (CPh_3), 92.07 (C1'), 113.48 (C_{Ar}); 115.71 (C5); 115.91 (q, ^1J=281 Hz, CF_3), 127.19 (C_{Ar}); 128.16 (C_{Ar}), 128.33 (C_{Ar}), 130.34 (130.17) (C_{Ar}), 135.59 (C_{Ar}), 135.72 (C_{Ar}); 139.93 (C6); 144.59 (C_{Ar}), 157.56 (q, ^2J=36 Hz, $COCF_3$), 158.83 (C_{Ar}), 163.32 (C2), 168.79 (COOR), 168.87 (C4). HRMS (ESI) calcd. for $C_{41}H_{49}F_3N_3O_{10}SSi$ $[M+H]^+$ 860.2860, found 860.2860. (Figures S9-S10).

*5'-O-(4,4'-Dimethoxytrityl)-N-[(1-β-D-ribofuranosyl-1H-2-selenopyrimidin-5-yl)methyl]-N-trifluoroacetylglycine 2-(trimethylsilyl)ethyl ester (**2d**).* To generate NaSeH, selenium (82 mg, 1.04 mmol, 12 eq.) was suspended in anhydrous ethanol (0.8 ml) at 0 °C and then $NaBH_4$ (50 mg, 1.32 mmol, 15 eq.) was added. After 2 h, *S*-methyl-2-thiouridine **2c** (80 mg, 0.09 mmol, 1 eq.) was added and the mixture was stirred for 1 h at room temperature. The reaction mixture was then evaporated under reduced pressure. The residue was dissolved in the mixture of ethyl acetate – water (6 ml, 1:1 by vol). The organic phase was separated and water was extracted several times with ethyl acetate (6 x 3 ml). Organic layers were combined, dried over anhydrous $MgSO_4$ and evaporated under reduced pressure. The residue was purified by flash column chromatography (2 % methanol in DCM) using argon overpressure. Pure selenouridine **2d** was obtained in 84 % yield (69 mg). TLC ($CHCl_3$:MeOH, 95:5, v/v) *Rf* =0.51; ^{1}H NMR (700 MHz, $CDCl_3$) δ (ppm): 0.05 (s, 9H, $SiMe_3$), 0.97-1.0 (m, 2H, $SiCH_2$), 3.55-3.59 (m, 2H, H5', H5"), 3.66 (d, 1H, ^2J=14.0 Hz, CH-5,1), 3.69 (d, 1H, ^2J=14.0 Hz, CH-5,1), 3.79 (s, 6H, $2xOCH_3$), 4.21-4.24 (m, 2H, $CH_2OC(O)$), 4.25-4.28 (m, 2H, H3', H4'), 4.39 (d, 1H, ^2J=18.9 Hz, CHC(O)O), 4.45-4.47 (m, 1H, H2'), 4.50 (d, 1H, ^2J=18.9 Hz, CHC(O)O), 6.41 (d, 1H, ^3J=1.4 Hz, H1'), 6.83-6.86 (m, 4H, H_{Ar}), 7.24 -7.46 (m, 9H, H_{Ar}), 8.14 (s, 1H, H6); ^{13}C NMR (176 MHz, $CDCl_3$) δ (ppm): -1.47 ($SiMe_3$), 17.51 ($SiCH_2$), 47.06 (CH_2-5.1), 51.08 ($CH_2C(O)$), 55.27 ($2xOCH_3$), 62.53 (C5'), 64.36 ($CH_2OC(O)$), 69.95 (C3'), 76.29 (C2'), 84.71 (C4'), 87.03 (CPh_3), 96.96 (C1'), 113.49 (C_{Ar}), 114.85 (C5), 115.95 (q, ^1J=287.76 Hz, CF_3), 127.16 (C_{Ar}), 128.16 (C_{Ar}), 128.38 (C_{Ar}), 130.37 (C_{Ar}), 130.44 (C_{Ar}), 135.50 (C_{Ar}), 135.82 (C_{Ar}), 141.64 (C6), 144.69 (C_{Ar}), 157.46 (q, ^2J=36.43 Hz, $COCF_3$), 158.82 (C_{Ar}), 158.83 (C4), 168.57 (COOR), 175.26 (C2); HRMS (ESI) calcd. for $C_{40}H_{45}F_3N_3O_{10}SeSi$ $[M-H]^+$ 892.1992, found 892.1986. (Figures S11-S12).

*5-(N-Trifluoroacetyl)carboxymethylaminomethyl-2-selenouridine (**2f**).* Protected selenouridine **2d** (48 mg, 0.05 mmol, 1 eq.) was dissolved in 1 M solution of TBAF in THF (421 µl, 0.423 mmol, 8 eq.). The mixture was stirred for 50 min at room temperature. After conversion (TLC analysis), $CaCO_3$ (88 mg), dry DOWEX 50WX8 H form (263 mg) and anhydrous methanol (0.6 ml) were added. The mixture was stirred for 1 h at room temperature and then filtered and washed with MeOH. The filtrate was evaporated under reduced pressure. The crude 5'-DMTr-*N*-TFA-2-selenouridine **2e** was treated with 50% aq. AcOH (2 ml). The reaction mixture was stirred for 1 h at room temperature and evaporated under reduced pressure. The residue was partitioned between chloroform (2 ml) and water (5 ml). The water phase was washed with chloroform (2 ml). The water layers were combined, and concentrated under reduced pressure. The solution was passed through the column with Dowex 50WX8 (pyridinium form) and eluted with mixture of water-pyridine (1:1, v/v). Fraction containing compound **2f** (TLC control) was concentrated under reduced pressure, lyophilized and purified by flash column chromatography (50 % methanol in chloroform) using argon overpressure. Compound **2f** was obtained in 70 % yield (18 mg, yield refers to **2d**) as a mixture of rotamers along the $NC(O)CF_3$ amide bond in a 0.75: 0.25 ratio according to ^{1}H NMR. Consequently, two chemical shifts are observed for some of the ^{1}H and ^{13}C NMR resonances (the secondary shifts in ^{13}C NMR spectra are given in parentheses). TLC (BuOH/H_2O, 85/15, v/v) R_f=0.32; ^{1}H NMR (700 MHz, D_2O) δ (ppm): 3.84-4.09 (m, 3H, H5', H5'', CH-5,1), 4.19-4.25 (m, 2H, H3', H4', CH-5,1), 4.37-4.59 (m, 3H, H2', $CH_2C(O)$), 6.78 (d, 0.75H, ^3J=2.8 Hz, H1'), 6.79 (d, 0.25H, ^3J=1.4 Hz, H1'), 8.23 (s, 0.25H, H6), 8.30 (s, 0.75H, H6); ^{13}C NMR (176 MHz, D_2O) δ (ppm): 45.83 (45.51) (CH_2-5.1), 51.74 (50.74) ($CH_2C(O)$), 59.88 (58.92) (C5'), 68.47 (68.06) (C3'), 75.14 (75.36) (C2'), 84.17 (84.11) (C4'), 95.98 (96.03) (C1'), 115.00 (115.28) (C5), 116.48 (q, ^1J=286.88 Hz, CF_3), 140.80 (140.41) (C6), 158.47 (q, ^2J=35.55 Hz, $COCF_3$), 162.00 (C4), 173.94 (COOH), 174.82 (175.04) (C2). HRMS (ESI) calcd. for $C_{14}H_{15}F_3N_3O_8Se$ $[M-H]^+$ 489.9976, found 489.9977. (Figures S13-S14).

*5-Carboxymethylaminomethyl-2-selenouridine (**2**).* 5-(*N*-trifluoroacetyl) carboxymethyl-aminomethyl-2-selenouridine (**2f**) (15 mg, 0.03 mmol) was dissolved in 30 % aq. ammonium (0.8 ml). After stirring at room temperature for 1 h the mixture was evaporated under reduced pressure. The residue was lyophilized and purified by RP HPLC on preparative C18 column SUPELCO; Ascentis® (25 cm/21.2 mm; 10 µm; flow 6 ml/min) using water as an eluent. Compound **2** was obtained in 82 % yield (8 mg). TLC (BuOH/H_2O, 85/15, v/v) R_f= 0.11; ^{1}H NMR (700 MHz, D_2O) δ (ppm): 3.64-3.69 (m, 2H, $CH_2C(O)O$), 3.93 (dd, 1H, ^2J=13.3 Hz, ^3J=2.1 Hz, H5''), 4.05 (d, 1H, ^2J=14.0 Hz, CH_2-5,1), 4.09 (d, 1H, ^2J=14.0 Hz, CH_2-5,1), 4.10 (dd, 1H, ^2J=13.3 Hz, ^3J=2.1 Hz, H5'), 4.24-4.26 (m, 2H, H3', H4'), 4.48 (dd, ^3J=2.1 Hz, ^3J=4.2 Hz, 1H, H2'), 6.69 (d, 1H, ^3J=2.1 Hz, H1'), 8.52 (s, 1H, H6); ^{13}C NMR (176 MHz, D_2O) δ (ppm): 44.77 (CH_2-5,1), 48.66 ($CH_2C(O)$), 59.33 (C5'), 67.91 (C3'), 75.05 (C2'), 84.02 (C4'), 95.99 (C1'), 110.80

(C5), 142.33 (C6), 170.90 (C4), 176.29 (C2). HRMS (ESI); calcd. for $C_{12}H_{16}N_3O_7Se$ $[M-H]^+$ 394.0153, found 394.0154. (Figures S15-S16).

Synthesis of 2-selenouridine (3)

2-Selenouridine (**3**) was obtained according to the previously described procedure (Sun,H., Sheng,J., Hassan,A.E., Jiang,S., Gan,J. and Huang,Z. (2012) Novel RNA base pair with higher specificity using single selenium atom. Nucleic Acids Res., 40, 5171-5179.) (Scheme S1, Figures S17-S25) with the total yield of 57 %.

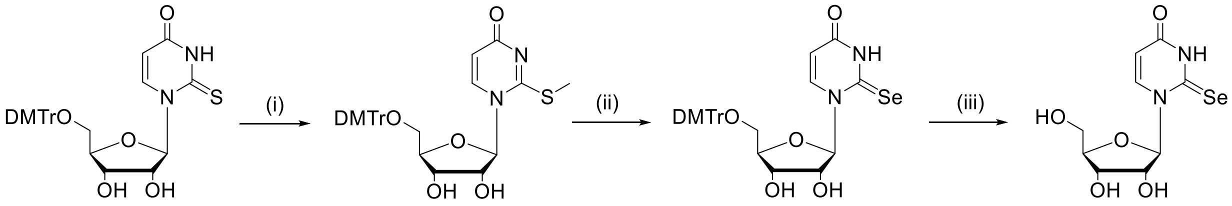

Scheme S1. Synthesis of 2-selenouridine (**3**). Reagents and conditions: (i) MeI, Et_3N, EtOH, rt, 2h, 86%; (ii) Se, $NaBH_4$, EtOH, 2 h, 0 °C to generate NaSeH, then 60°C, 2.5 h, 70 %; (iii) TCA-DCM (3/100, v/v), 1 min, rt, 95 %; DMTr = 4,4'-dimethoxytrityl

*5'-O-(4,4'-Dimethoxytrityl)-S-methyl-2-thiouridine (**3b**).* 5'-*O*-(4,4'-Dimetoxytrityl)-2-thiouridine (**3a**) (840 mg, 1.49 mmol, 1 equiv) was dissolved in anhydrous ethanol (14.9 ml) and triethylamine (622 µl, 4.48 mmol, 3 equiv) and methyl iodide (278 µl, 4.48 mmol, 3 equiv) were added. The solution was stirred for 3.5 h at room temperature. Ethanol was removed under reduced pressure and the solid residue was dissolved in DCM (50 ml) and washed with water (25 ml). The water phase was extracted with DCM (2 x 50 ml). The organic layers were combined and dried with anhydrous $MgSO_4$. After filtration, the organic solvent was evaporated *in vacuo*. The residue was co-evaporated with anhydrous toluene and purified on column of silica gel with 3 % methanol in chloroform. Compound **3b** was afforded in 86 % (771 mg) as a yellow foam. TLC ($CHCl_3$: MeOH, 95 : 5, v/v) R_f=0.23, TLC ($CHCl_3$/MeOH, 9 : 1, v/v) R_f =0.76, ^{1}H NMR (700 MHz, acetone-d_6) δ (ppm): 2.55 (s, 3H, -SCH_3), 3.47 (dd, 1H, ^3J=2.8 Hz, ^2J=10.5 Hz, H5''), 3.53 (dd, 1H, ^3J=2.8 Hz, ^2J=10.5 Hz, H5'), 3.80 (s, 6H, 2 x-OCH_3), 4.20 – 4.22 (m, 1H, H4'), 4.50-4.54 (m, 2H, H2', H3'), 5.59 (d, 1H, ^3J=7.7 Hz, H5), 5.88 (d, 1H, ^3J=4.2 Hz, H1'), 6.92-6.95 (m, 4H, H_{Ar}), 7.25-7.51 (m, 9H, H_{Ar}), 8.02 (d, 1H, ^3J=7.7 Hz, H6); ^{13}C NMR (176 MHz, acetone-d_6) δ (ppm): 13.90 (-SCH_3), 54.68 (-OCH_3), 62.73 (C5'), 70.17 (C3'), 75.23 (C2'), 84.02 (C4'), 86.84 (-CPh_3), 92.09 (C1'), 108.67 (C5), 113.20 (C_{Ar}), 126.92 (C_{Ar}), 127.91 (C_{Ar}), 130.15 (C_{Ar}), 135.34 (C_{Ar}), 135.61 (C_{Ar}), 139.01 (C6), 144.86 (C_{Ar}), 158.88 (C_{Ar}), 162.51 (C2), 167.08 (C4). HRMS (ESI); calcd. for $C_{31}H_{33}N_2O_7S$ $[M+H]^+$ 577.2013, found 577.2008 (Figures S17-S18).

*5'-O-(4,4'-Dimethoxytrityl)-2-selenouridine (**3c**).* Selenium (136.8 mg, 1.73 mmol, 10 equiv) was suspended in anhydrous ethanol (1.1 ml) and $NaBH_4$ (98.3 mg, 2.59 mmol, 15 equiv) was added at 0 °C. NaSeH was generated for 1 h at 0 °C and then 5'-*O*-DMTr-*S*-methyl-2-thiouridine **3b** (100 mg, 0.17 mmol, 1 equiv) was added. The reaction mixture was stirred in the heating bath at 60 °C. The reaction was monitored by TLC (5 % methanol in chloroform) and completed in 2 h. Solution was evaporated under reduced pressure and the solid residue was portioned between ethyl acetate (5 ml) and water (2 ml). The water phase was extracted with ethyl acetate several times (4 x 5 ml). The organic layers were combined and dried with anhydrous $MgSO_4$. The filtrate was evaporated under reduced pressure and purified by flash column chromatography (1 % methanol in chloroform) to afford **3c** in 70 % yield (70 mg) as a yellow foam. TLC ($CHCl_3$: MeOH, 9 : 1) R_f=0.65, TLC ($CHCl_3$: MeOH, 95 : 5) R_f= 0.49; ^{1}H NMR (700 MHz, $CDCl_3$) δ (ppm): 3.98 (dd, 1H, ^3J=2.1 Hz, ^2J=11.2 Hz, H5''), 3.61-3.65 (m, 1H, H5'), 3.82 (s, 6H, 2 x OCH_3), 4.24 – 4.26 (m, 1H, H4'), 4.50-4.54 (m, 2H, H2', H3'), 5.71 (d, 1H, ^3J=8.4 Hz, H5), 6.55 (bs, 1H, H1'), 6.68-6.89 (m, 4H, H_{Ar}), 4.26-7.42 (m, 9H, H_{Ar}), 8.25 (d, 1H, ^3J=8.4 Hz, H6); ^{13}C NMR (176 MHz, $CDCl_3$) δ (ppm): 55.30 (2 x OCH_3), 61.21 (C5'), 69.19 (C3'), 76.23 (C2'), 84.29 (C4'), 87.19 (-CPh_3), 96.62 (C1'), 108.22 (C5), 113.41 (C_{Ar}), 127.25 (C_{Ar}), 128.10 (C_{Ar}), 130.11 (C_{Ar}), 130.18

(C_{Ar}), 135.04 (C_{Ar}), 135.25 (C_{Ar}), 140.99 (C6), 144.30 (C_{Ar}), 158.77 (C2), 175.60 (C4). HRMS (ESI); calcd. for $C_{30}H_{30}N_2O_7SeNa$ $[M+H+Na]^+$ 633.1115, found 633.1116 (Figures S19-S20).

*2-Selenouridine (**3**).* 5'-DMTr-2-selenouridine (**3c**) (160 mg, 0.26 mmol) was dissolved in 5 ml trichloroacetic acid/ dichloromethane 3/100 (v/v). After stirring at room temperature for 1 minute, the mixture was partitioned between dichloromethane (5 ml) and water (15 ml). The water phase was washed with dichloromethane seven times (5 ml). The water layer was concentrated under reduced pressure. The residue was lyophilized and purified by RP HPLC on preparative C18 column SUPELCO; Ascentis® (25 cm/21.2 mm; 10 µm; flow 6 ml/min) using a mixture of A: water and B: acetonitrile according to the following gradient: 0% B for 5 min.; 0→40 % B for 20 min. and 40% B from 25 to 30 min. Compound **3** was obtained in 95 % yield (76 mg). TLC ($CHCl_3$:MeOH, 85:15, v/v) *Rf* =0.46; ^1H NMR (700 MHz, D_2O) δ (ppm): 3.89-3.91 (m, 1H, H5'), 4.03-4.06 (m, 1H, H5"), 4.24-4.24 (m, 2H, H3', H4'), 4.46-4.47 (m, 1H, H2'), 6.33 (d, 1H, ^3J=8.1 Hz, H5), 6.77 (d, 1H, ^3J=2.1 Hz, H1'), 8.20 (d, 1H, ^3J=8.1 Hz, H5); ^1H NMR (700 MHz, DMSO-d6) δ (ppm): 3.60 (dd, 1H, ^3J=2.7 Hz, ^2J=12.2 Hz H5'), 3.70 (dd, 1H, ^3J=2.7 Hz, ^2J=12.2 Hz, H5"), 3.91-3.92 (m, 1H, H4'), 3.98-3.99 (m, 1H, H3'), 4.07-4.09 (m, 1H, H2'), 6.09 (d, 1H, ^3J=8.1 Hz, H5), 6.70 (d, 1H, ^3J=2.1 Hz, H1'), 8.18 (d, 1H, ^3J=8.1 Hz, H6), ^{13}C NMR (176 MHz, DMSO-d6) δ (ppm): 59.87 (C5'), 68.98 (C3'), 74.86 (C2'), 84.87 (C4'), 95.35 (C1'), 107.82 (C5), 140.87 (C6), 159.32 (C4), 176.44 (C2); ^{13}C NMR (176 MHz, D_2O) δ (ppm): 59.82 (C5'), 68.41 (C3'), 74.96 (C2'), 84.13 (C4'), 95.91 (C1'), 108.30 (C5), 141.63 (C6), 161.70 (C4), 175.79 (C2); ^{77}Se NMR (134 MHz, DMSO-d_6) δ (ppm):367; HRMS (ESI); calcd. for $C_9H_{13}N_2O_5Se$ $[M-H]^+$ 308.9990, found 308.9975 (Figures S21-S25).

Potentiometric measurements

The acidity constants of the nucleosides **1-3** (pKa) were determined by the pH-potentiometric titration of 2.0-ml samples. The concentration of the nucleoside in solution was 1×10^{-3} M. Measurements were carried out at 298 K and at a constant ionic strength of 0.1 M NaCl using a MOLSPIN pH meter (Molspin Ltd., Newcastle-upon-Tyne, UK) equipped with a digitally operated syringe (the Molspin DSI 0.250 ml) controlled by a computer. For the titrations, a carbonate-free NaOH solution of known concentration (0.1 M) was used and measurements were made using a Russel CMAWL/S7 semi-micro combined electrode, calibrated for hydrogen ion concentration using the method of Irving et al. The accepted fit for the titration curves was always less than 0.01 ml. The number of experimental points was 100–150 for each titration curve. The titration points included in the evaluation could be reproduced within 0.005 pH units in the whole pH range examined (pH from 2 to 12). The protonation constants of the nucleosides were evaluated by performing iterative non-linear least squares fit of the potentiometric equilibrium curves through mass balance equations using the computer program SUPERQUAD. The sigma value (the root mean squared weighted residual) obtained after the refinement of the stability constants was 1, which suggested that the data were fitted within experimental error. The equilibrium constants reported in this work were obtained from a fitting performed using three titration curves simultaneously.

2. **Spectral analysis of Se2U derivatives**

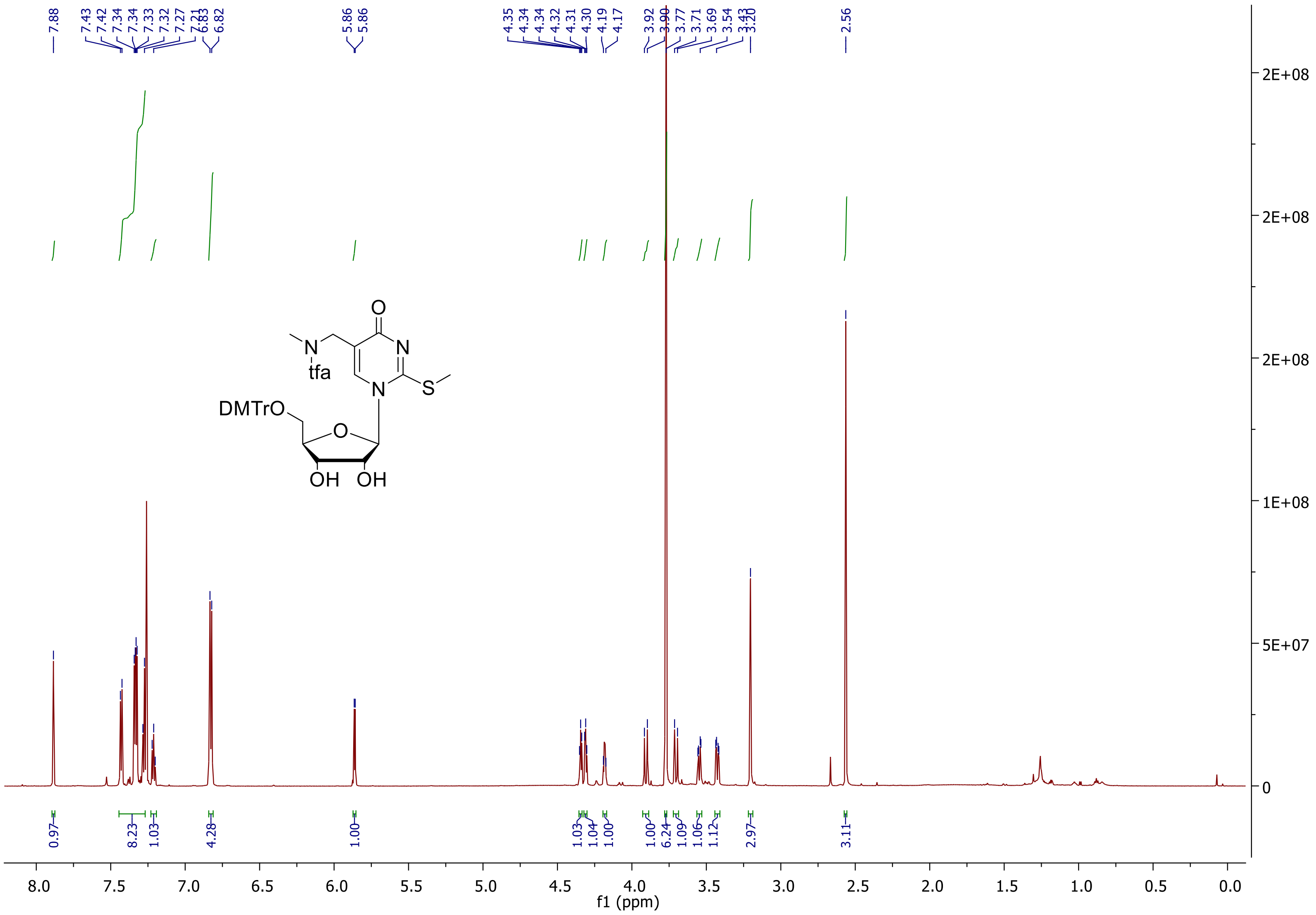

Figure S1. ^{1}H NMR (700 MHz, $CDCl_3$) 5’-*O*-(4,4’-dimethoxytrityl)-5-(*N*-trifluoroacetyl)methylaminometyl-*S*-methyl-2-thiouridine **(1c)**

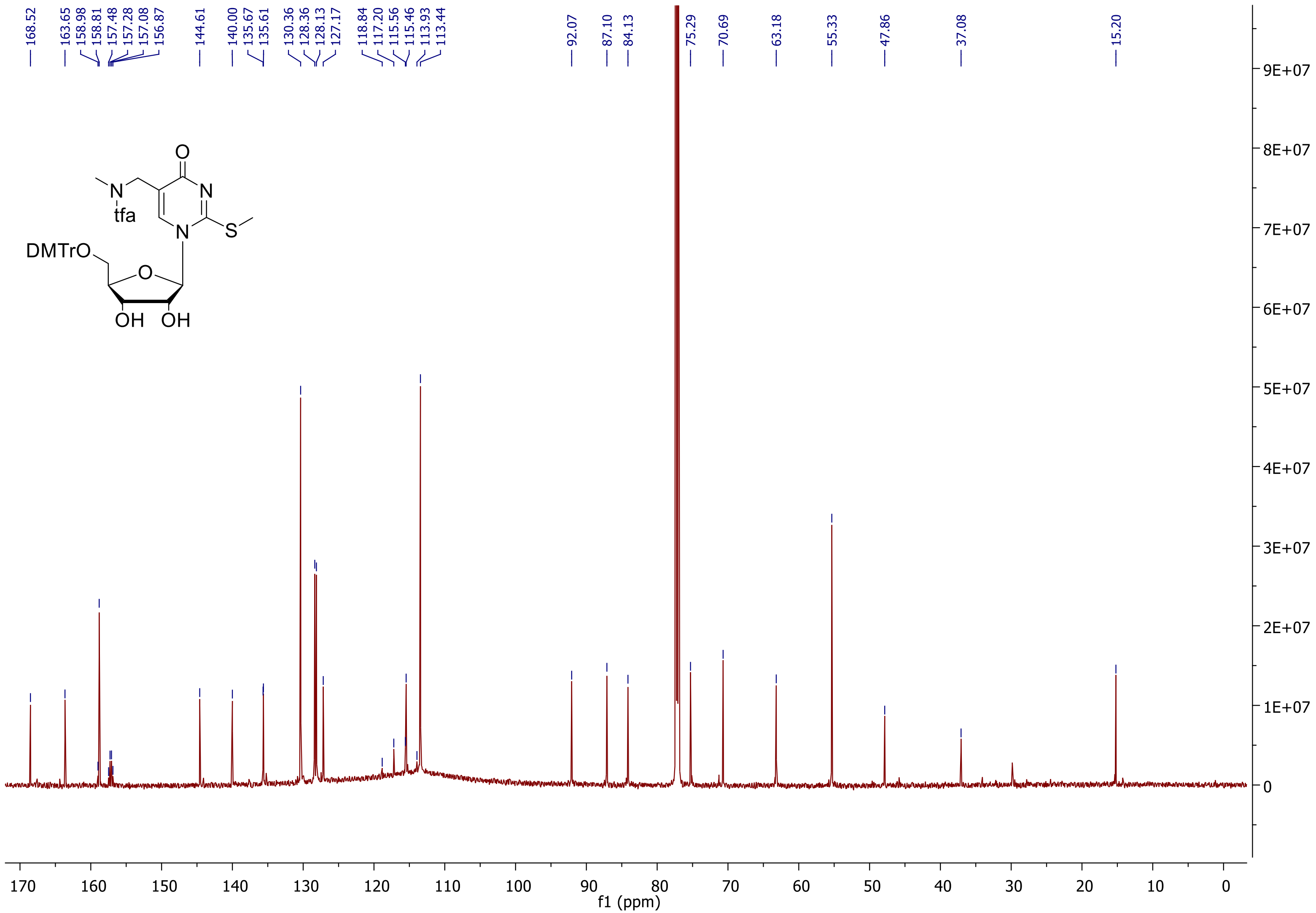

Figure S2. ^{13}C NMR (176 MHz, $CDCl_3$) 5’-*O*-(4,4’-dimethoxytrityl)-5-(*N*-trifluoroacetyl)methylaminometyl-*S*-methyl-2-thiouridine **(1c)**

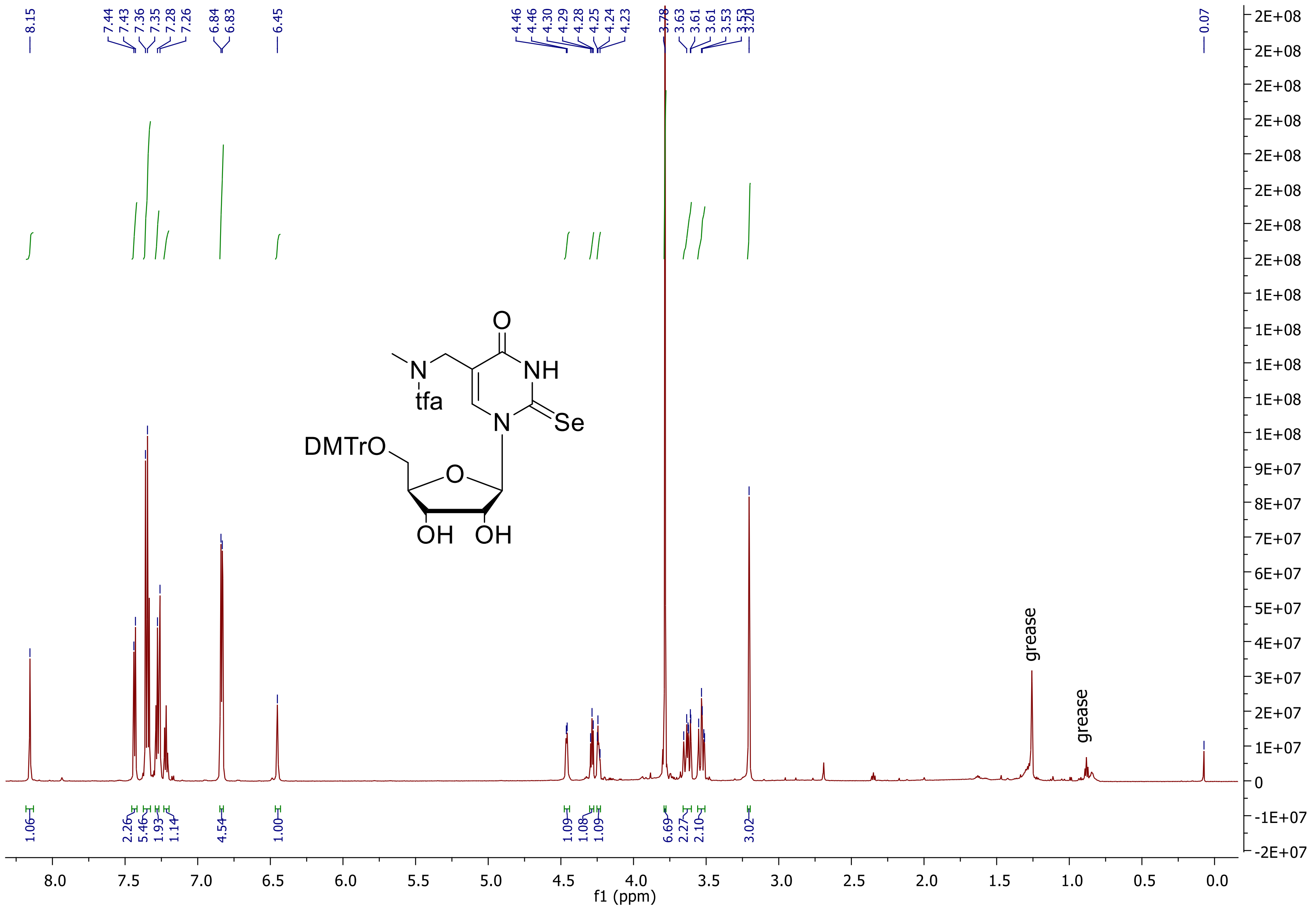

Figure S3. ^{1}H NMR (700 MHz, $CDCl_3$) 5’-*O*-(4,4’-dimethoxytrityl)-5-(*N*-trifluoroacetyl)methylaminomethyl-2-selenouridine **(1d)**

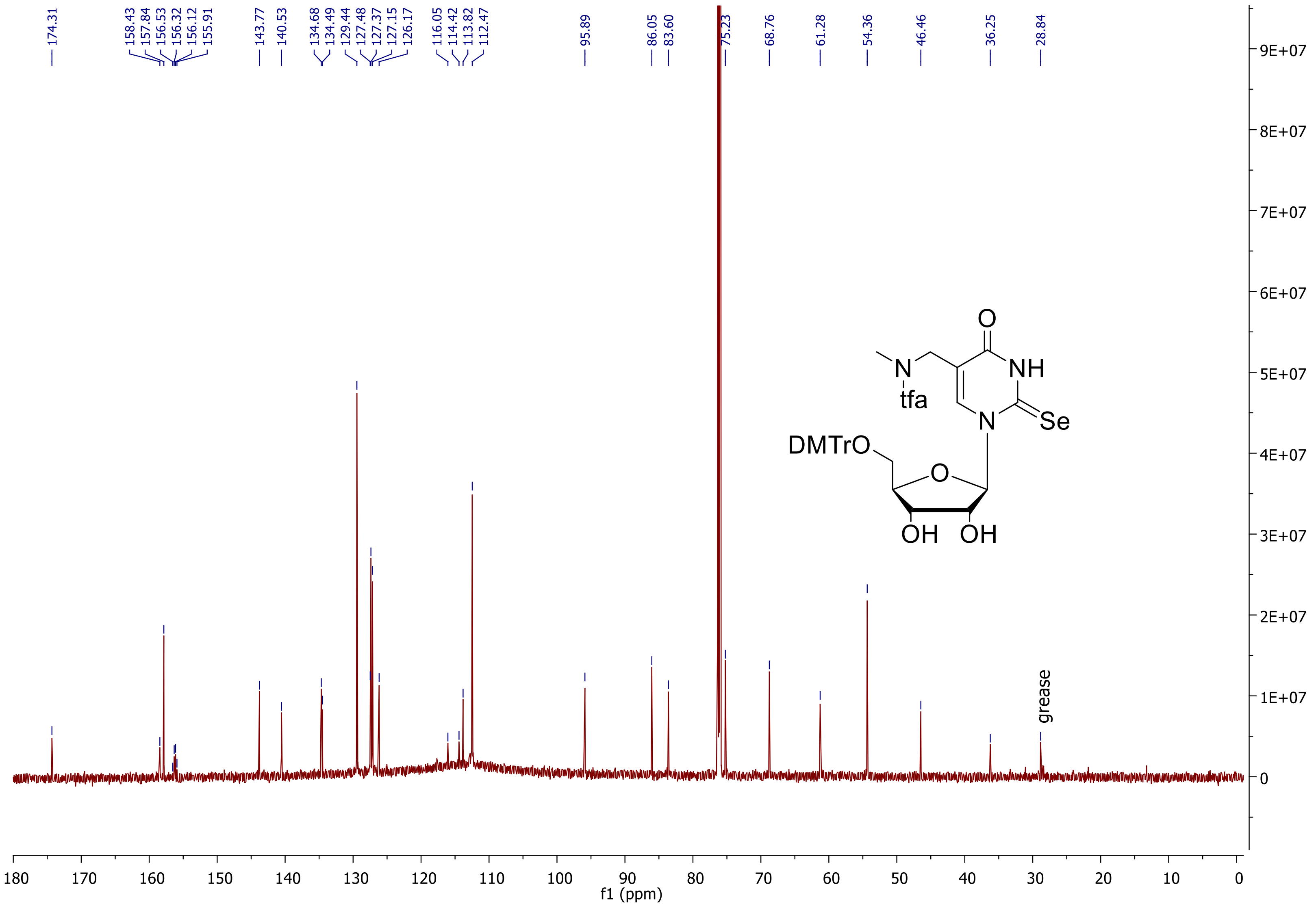

Figure S4. ^{13}C NMR (176 MHz, $CDCl_3$) 5'-*O*-(4,4'-dimethoxytrityl)-5-(*N*-trifluoroacetyl)methylaminomethyl-2-selenouridine **(1d)**

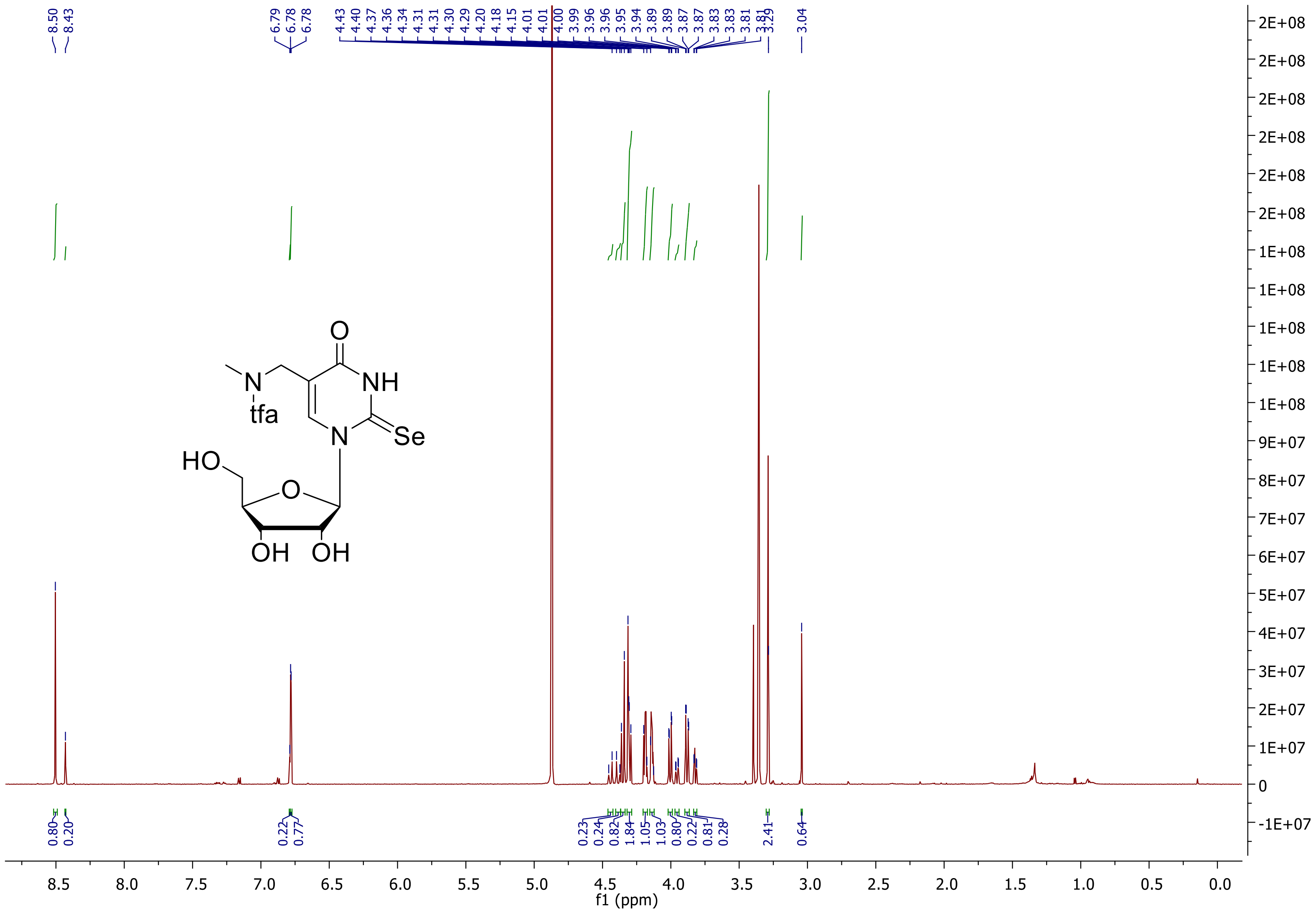

Figure S5. ^{1}H NMR (700 MHz, CD_3OD) 5-(*N*-trifluoroacetyl)methylaminomethyl-2-selenouridine **(1e)**

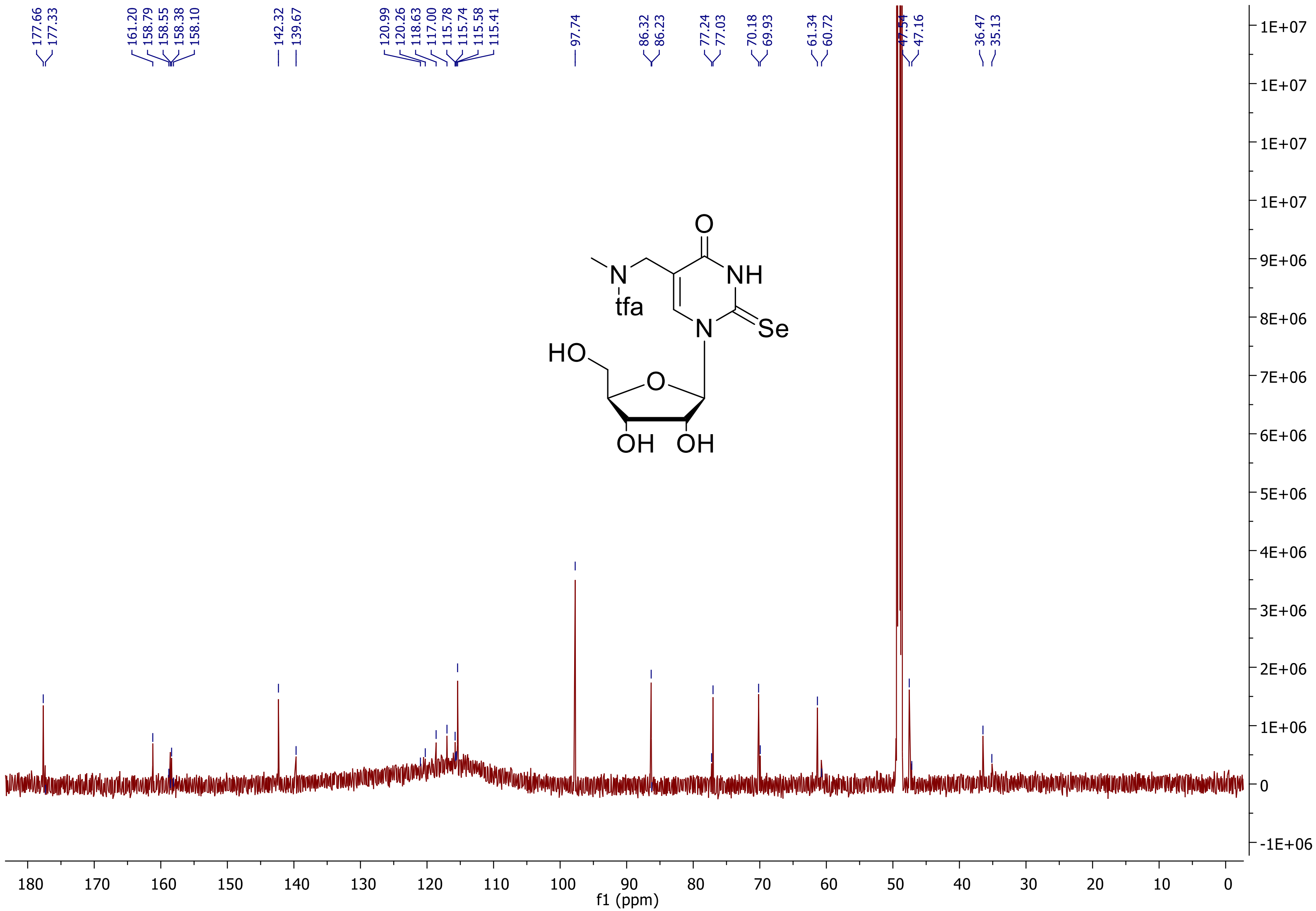

Figure S6. ^{13}C NMR (700 MHz, CD_3OD) 5-(*N*-trifluoroacetyl)methylaminomethyl-2-selenouridine **(1e)**

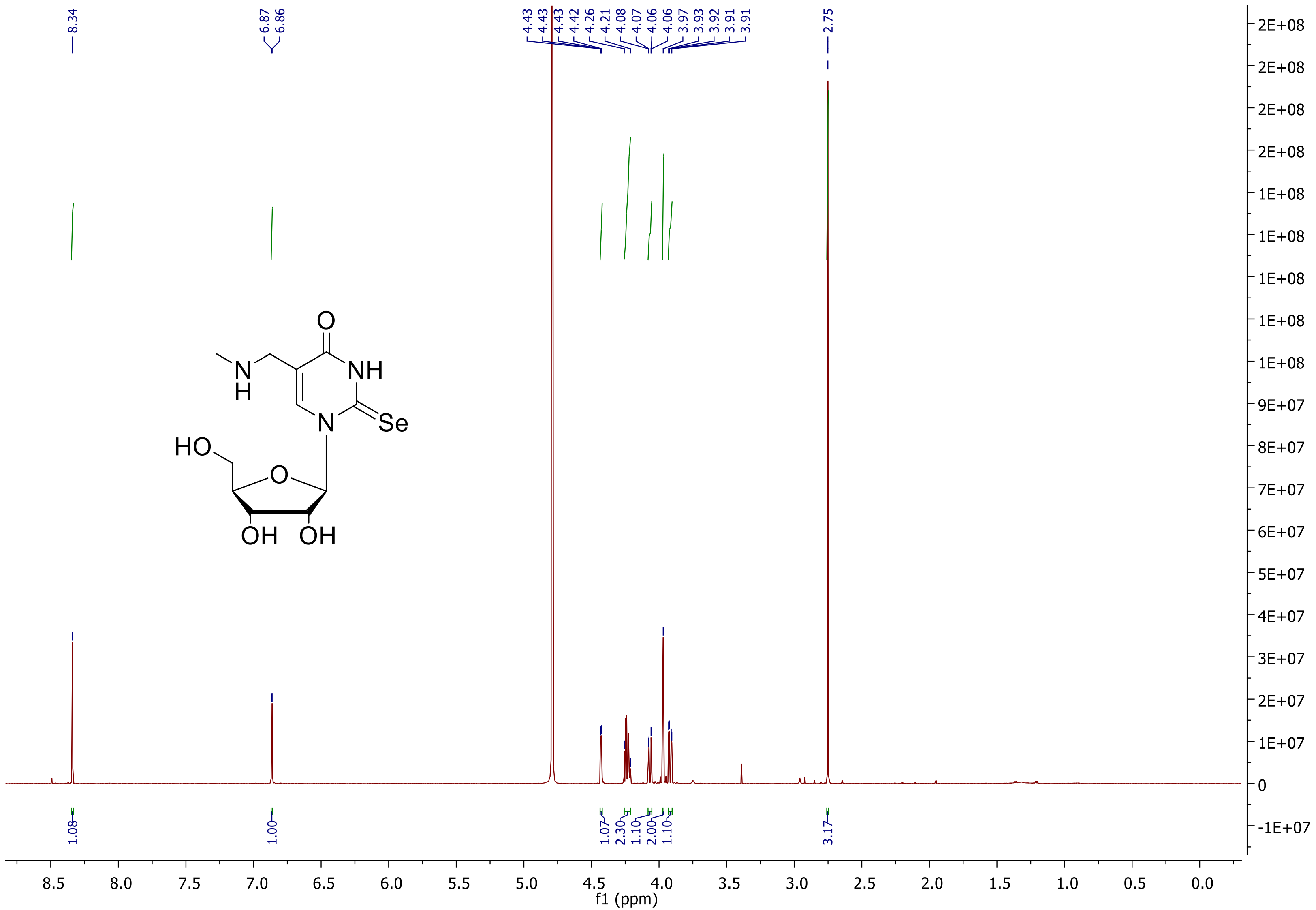

Figure S7. ^{1}H NMR (700 MHz, D_2O) 5-methylaminomethyl-2-selenouridine **(1)**

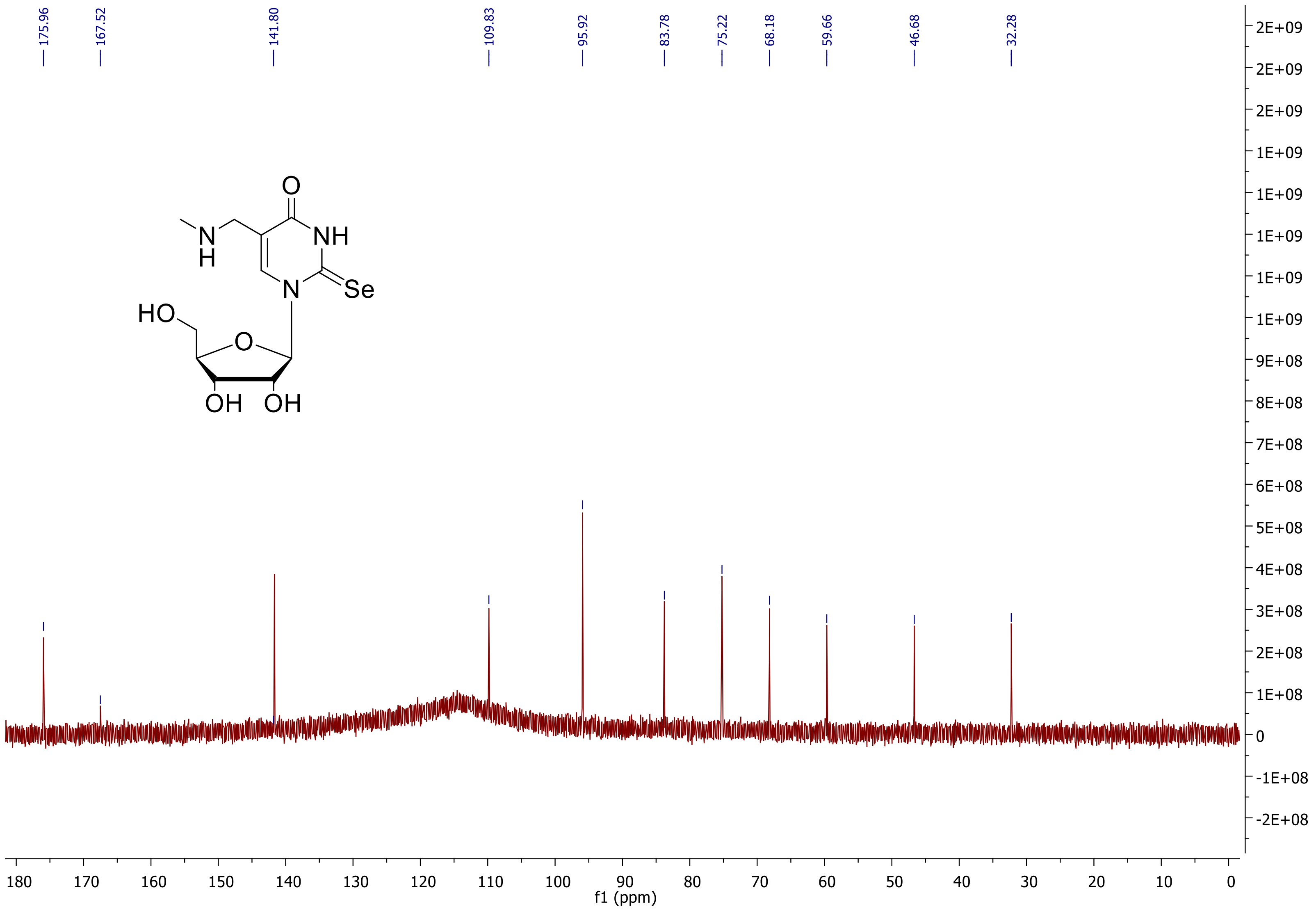

Figure S8. ^{13}C NMR (176 MHz, D_2O) 5-methylaminomethyl-2-selenouridine **(1)**

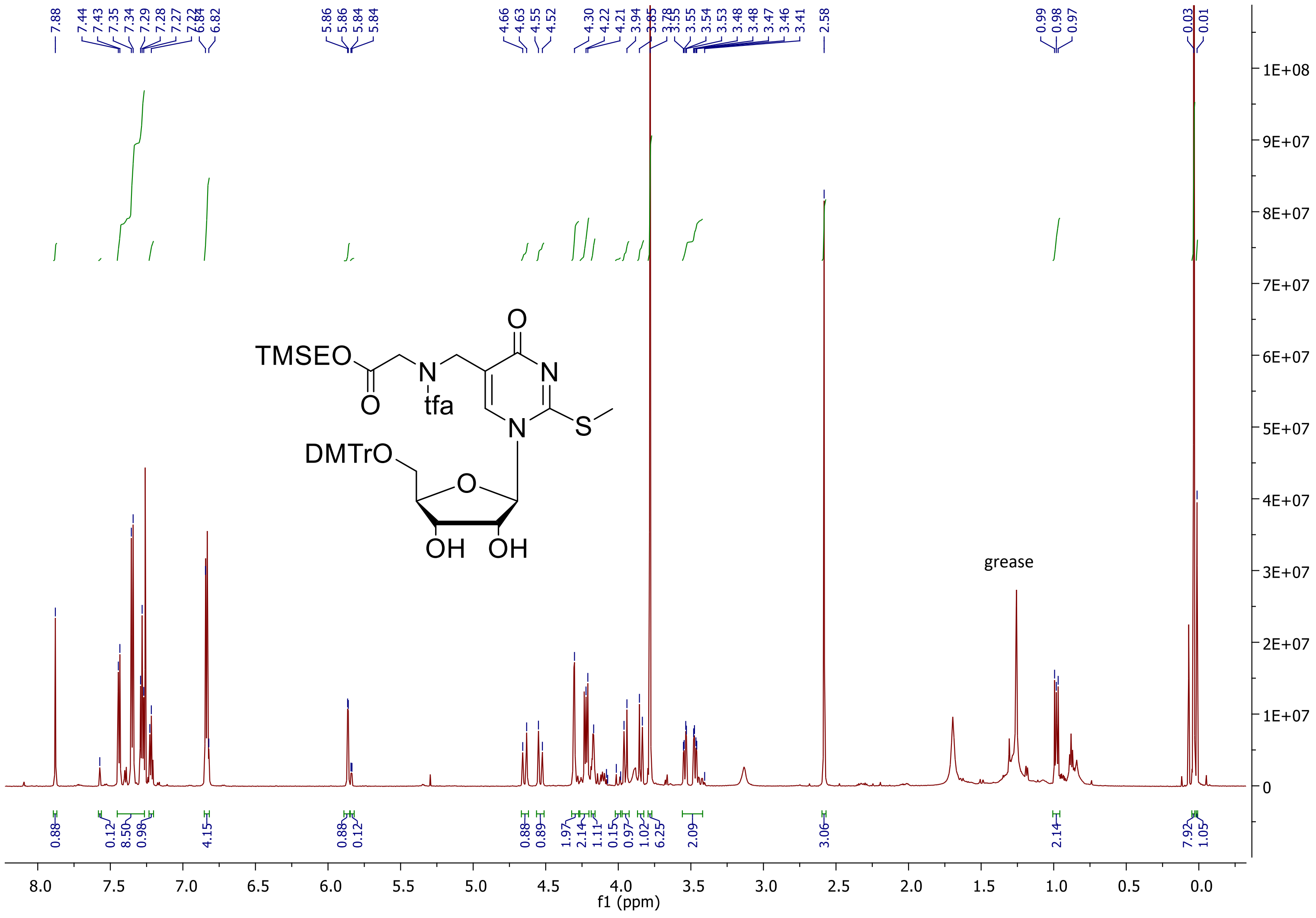

Figure S9. ^{1}H NMR (700 MHz, $CDCl_3$) 5'-*O*-(4,4'-dimethoxytrityl)-*N*-[(1-*β*-D-ribofuranosyl-1*H*-2-methylthiopyrimidin-5-yl)methyl]-*N*-trifluoroacetylglycine 2-(trimethylsilyl)ethyl ester **(2c)**

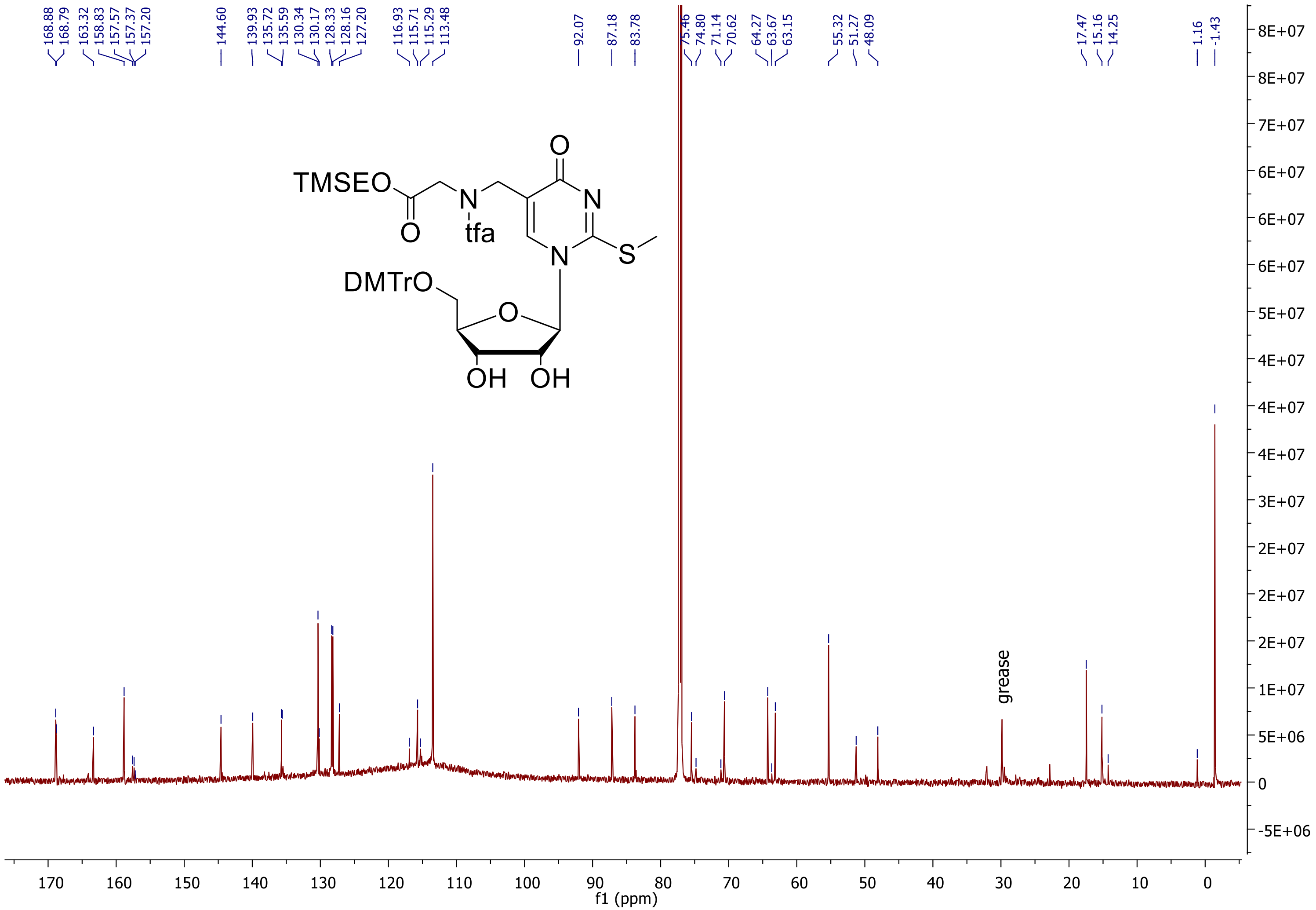

Figure S10. ^{13}C NMR (176 MHz, $CDCl_3$) 5'-*O*-(4,4'-dimethoxytrityl)-*N*-[(1-*β*-D-ribofuranosyl-1*H*-2-methylthiopyrimidin-5-yl)methyl]-*N*-trifluoroacetylglycine 2-(trimethylsilyl)ethyl ester **(2c)**

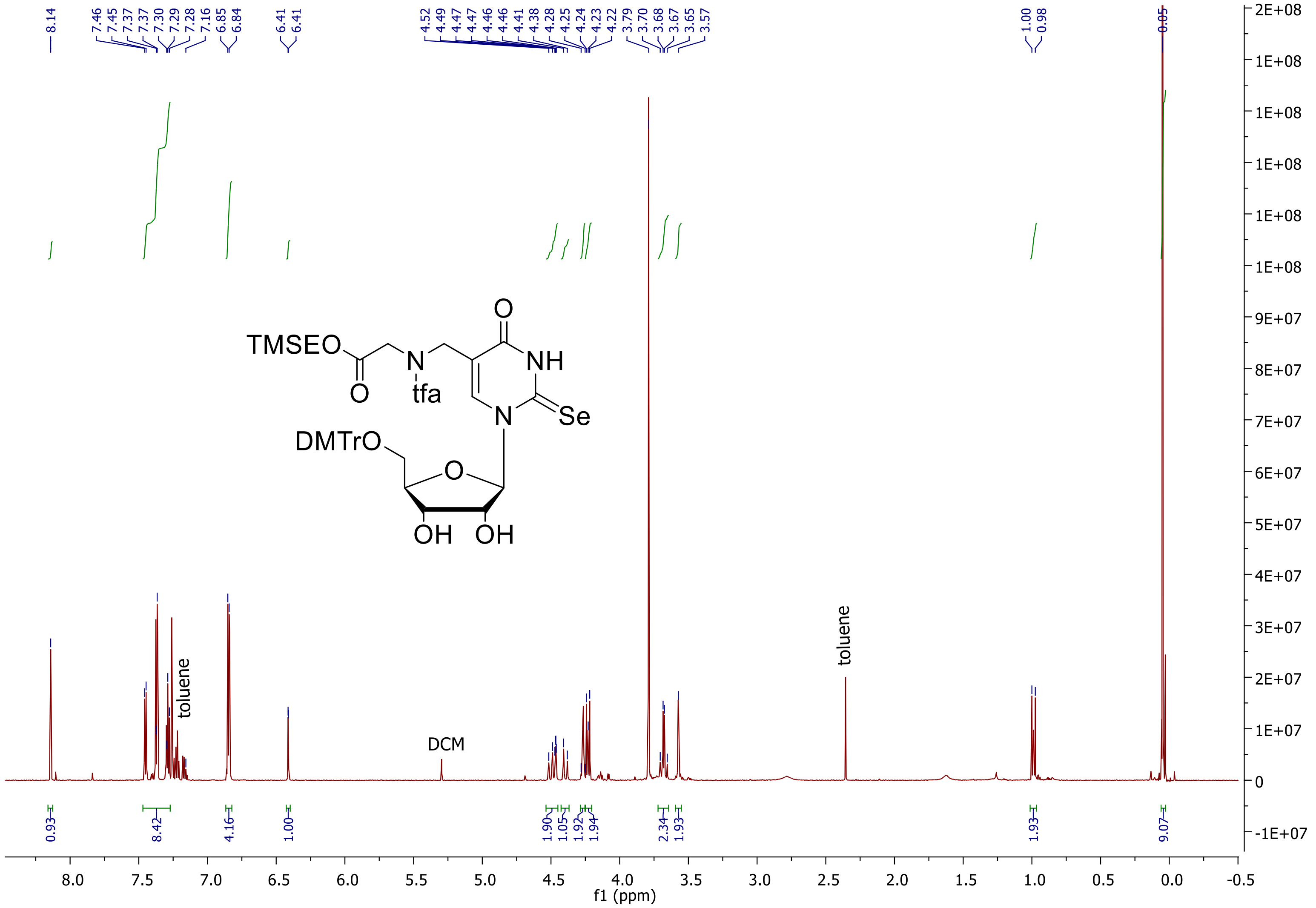

Figure S11. ^{1}H NMR (700 MHz, $CDCl_3$) 5'-*O*-(4,4'-dimethoxytrityl)-*N*-[(1-*β*-D-ribofuranosyl-1*H*-2-selenopyrimidin-5-yl)methyl]-*N*-trifluoroacetylglycine 2-(trimethylsilyl)ethyl ester **(2d)**

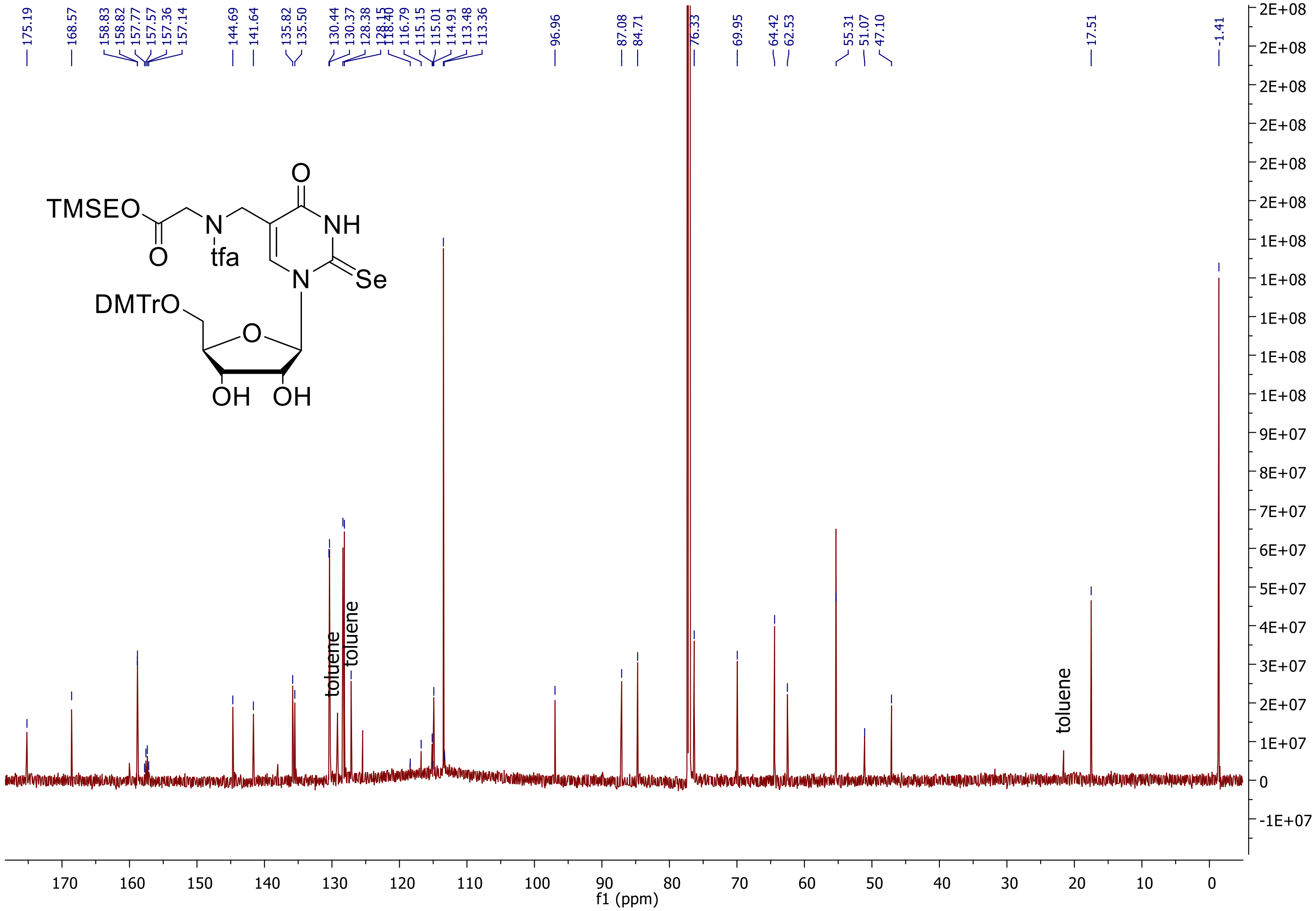

Figure S12. ^{13}C NMR (176 MHz, $CDCl_3$) 5'-*O*-(4,4'-dimethoxytrityl)-*N*-[(1-*β*-D-ribofuranosyl-1*H*-2-selenopyrimidin-5-yl)methyl]-*N*-trifluoroacetylglycine 2-(trimethylsilyl)ethyl ester **(2d)**

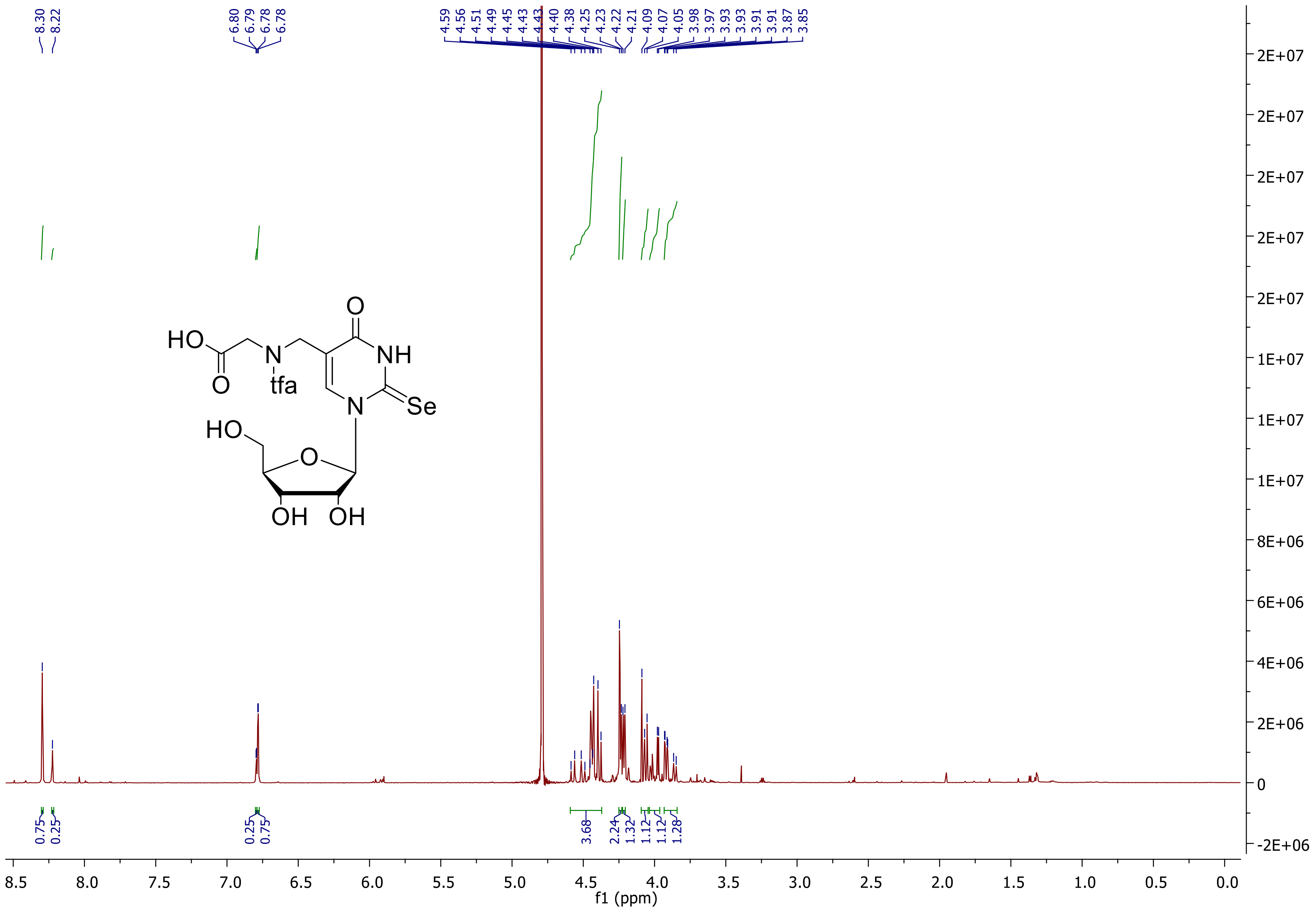

Figure S13. ^{1}H NMR (700MHz, D_2O) 5-(*N*-trifluoroacetyl)carboxymethylaminomethyl-2-selenouridine **(2f)**

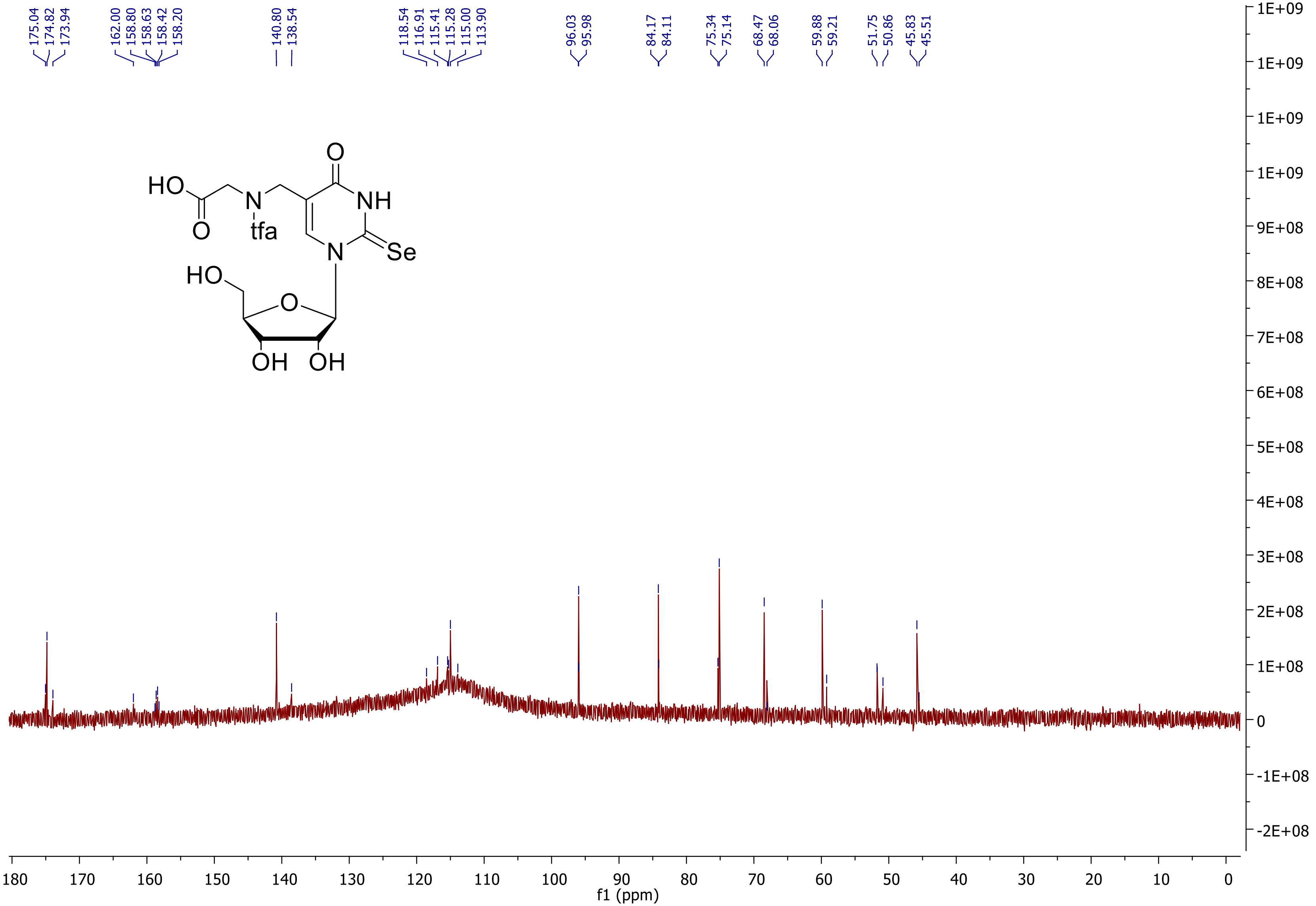

Figure S14. ^{13}C NMR (176 MHz, D_2O) 5-(*N*-trifluoroacetyl)carboxymethylaminomethyl-2-selenouridine **(2f)**

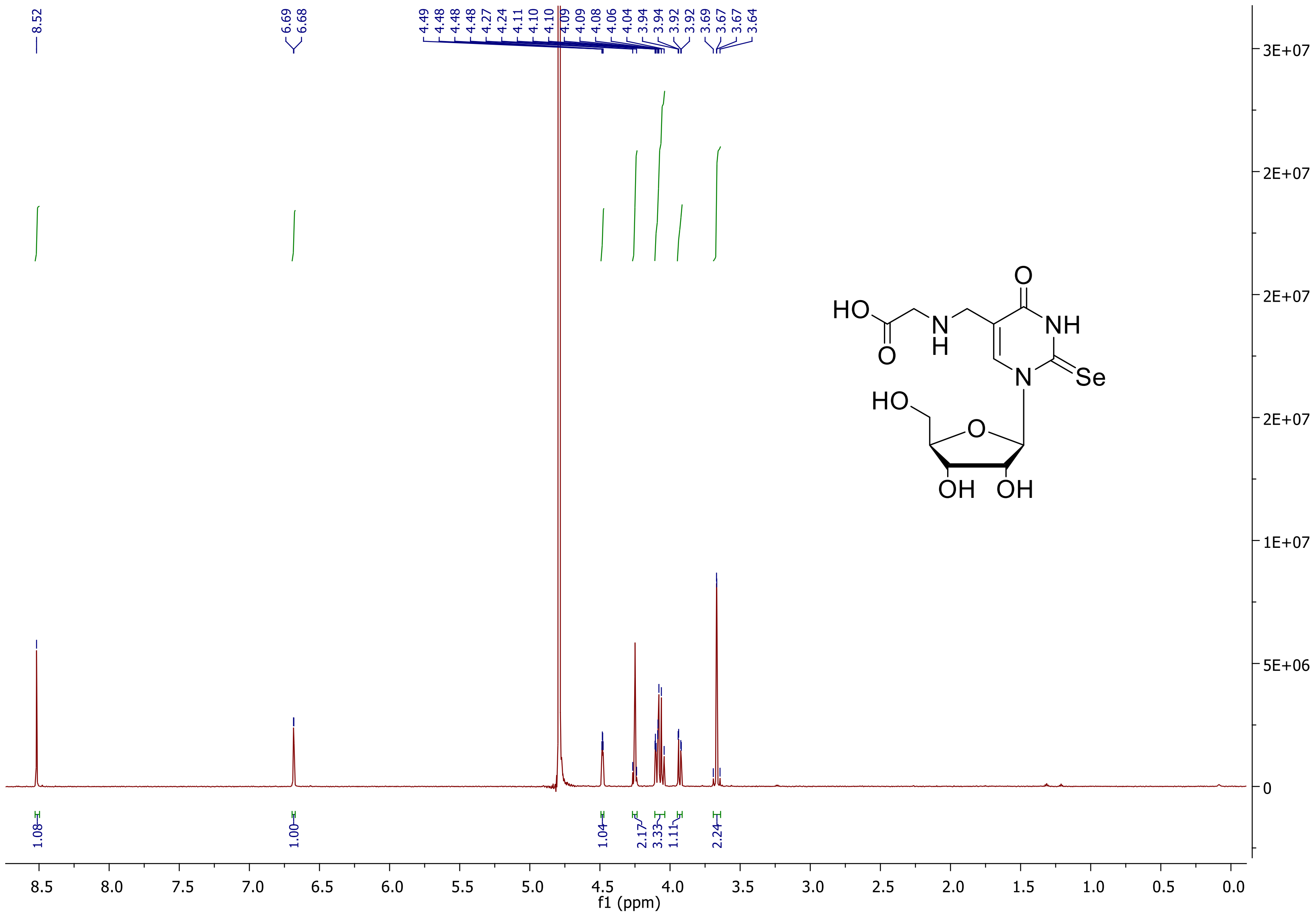

Figure S15. ^{1}H NMR (700 MHz, D_2O) 5-carboxymethylaminomethyl-2-selenouridine **(2)**

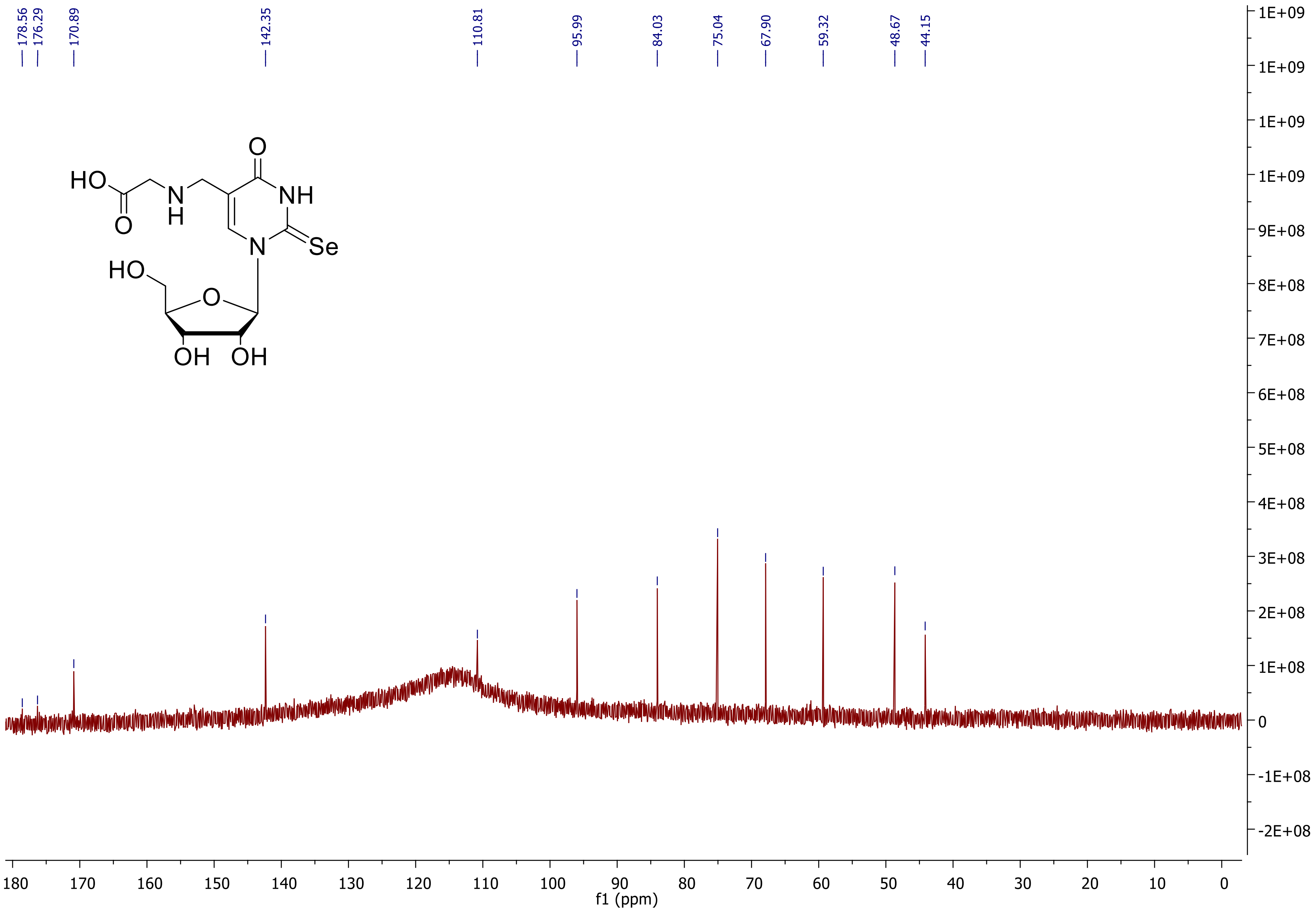

Figure S16. ^{13}C NMR (176 MHz, D_2O) 5-carboxymethylaminomethyl-2-selenouridine **(2)**

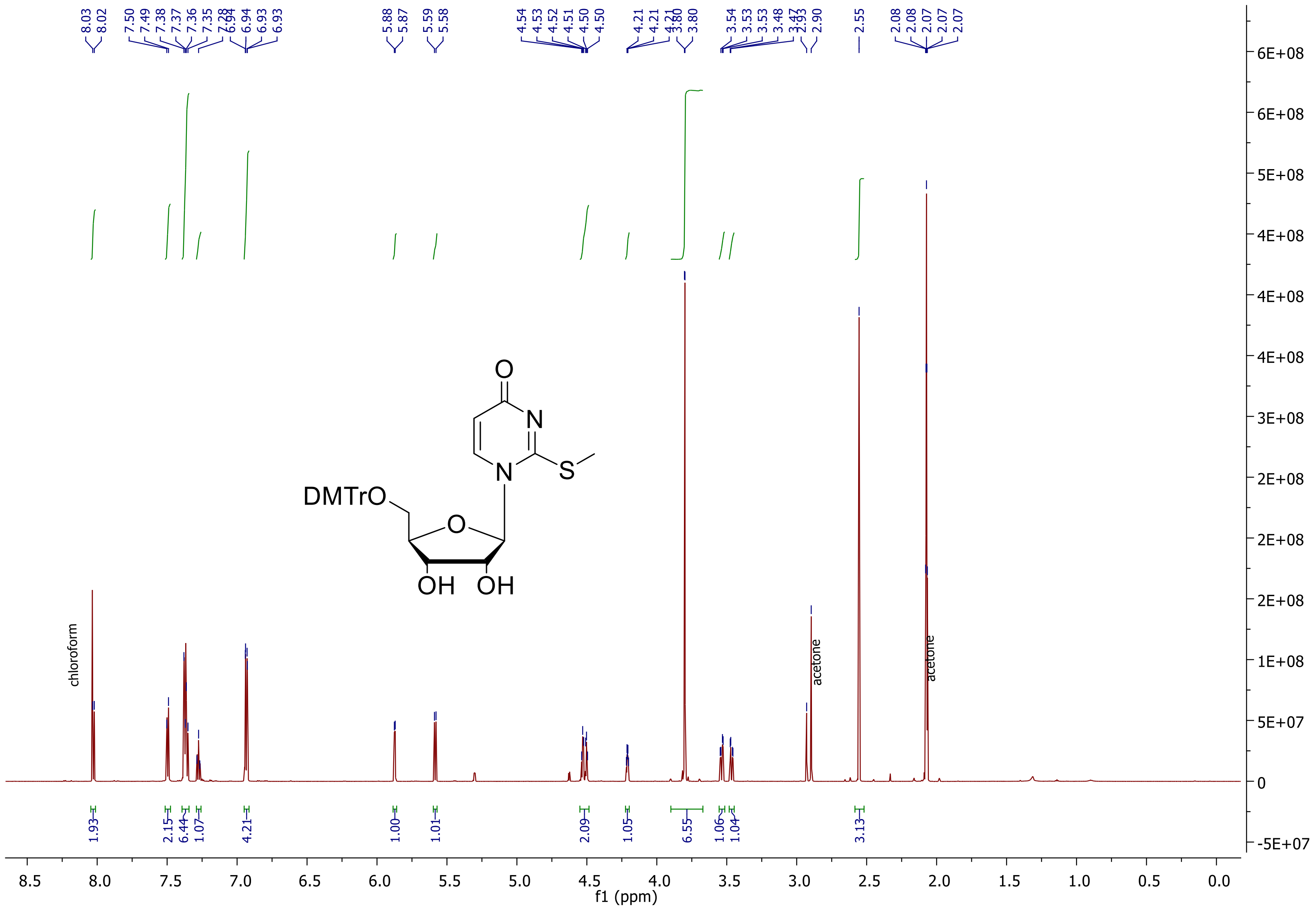

Figure S17. ^{1}H NMR (700 MHz, acetone-d$_6$) 5'-*O*-(4,4'-dimethoxytrityl)-*S*-methyl-2-thiouridine (**3b**).

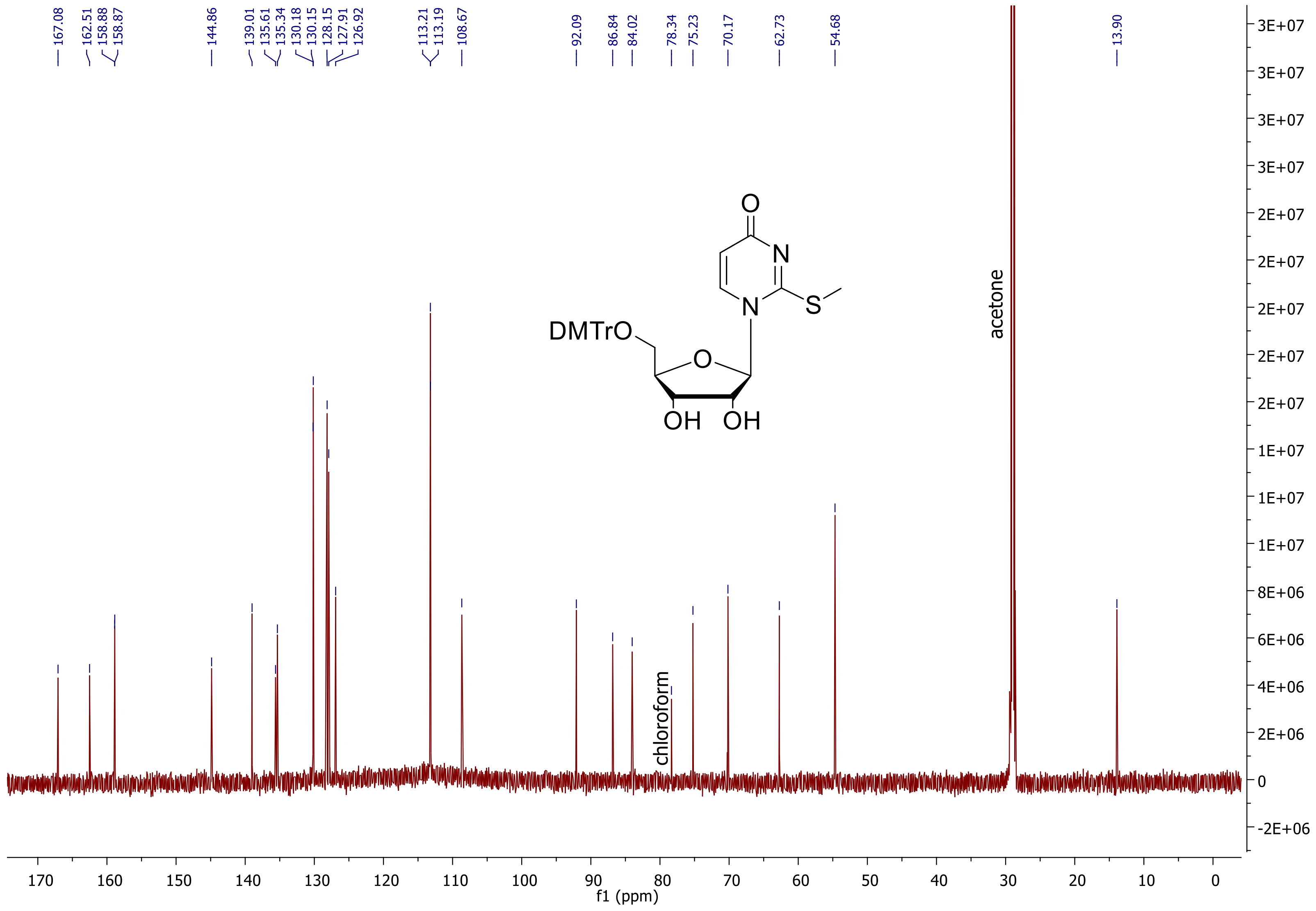

Figure S18. ^{13}C NMR (176 MHz, acetone-d_6) 5'-*O*-(4,4'-dimethoxytrityl)-*S*-methyl-2-thiouridine (**3b**).

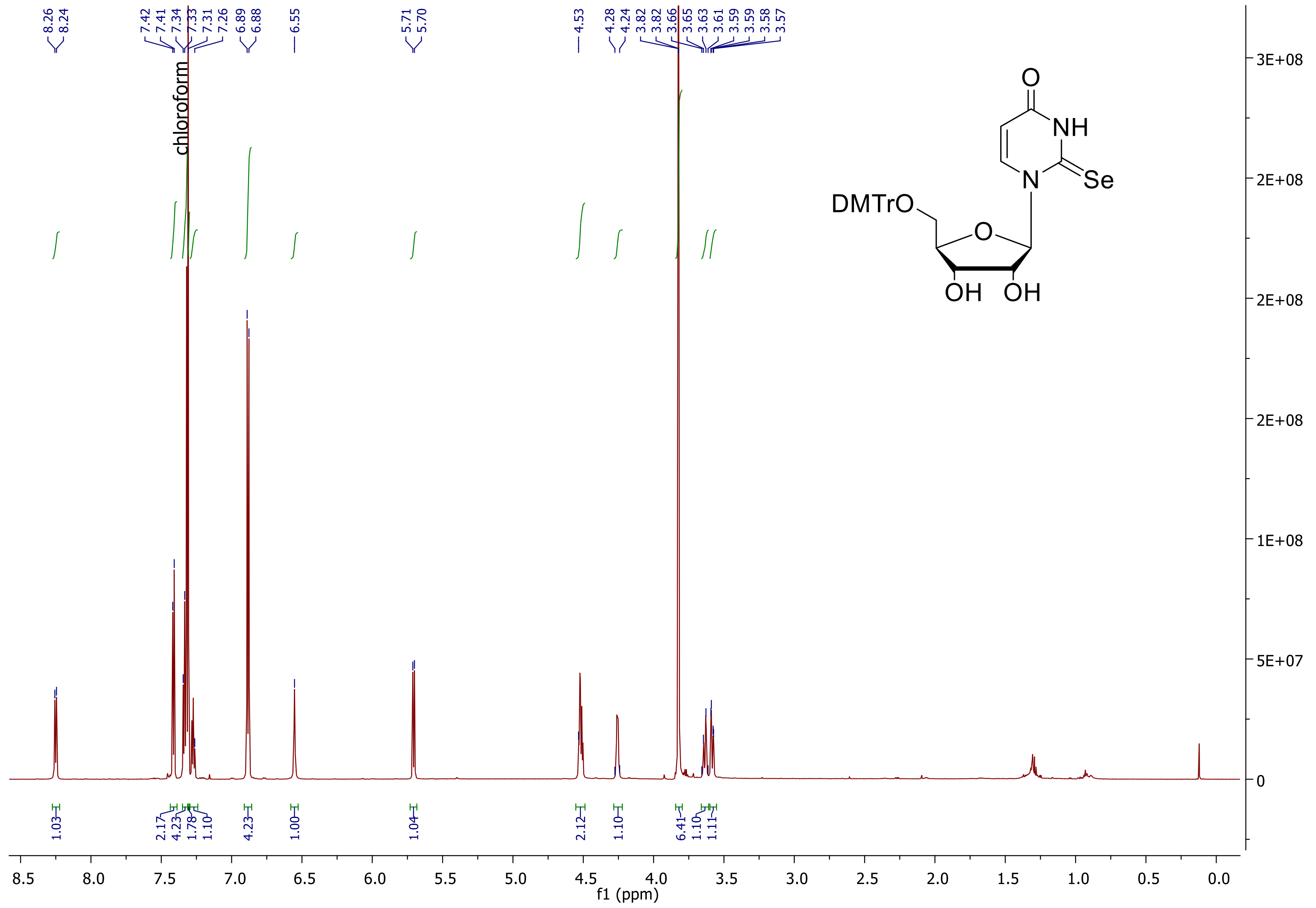

Figure S19. ^{1}H NMR (700 MHz, $CDCl_3$) 5'-*O*-(4,4'-dimethoxytrityl)- 2-selenouridine (**3b**).

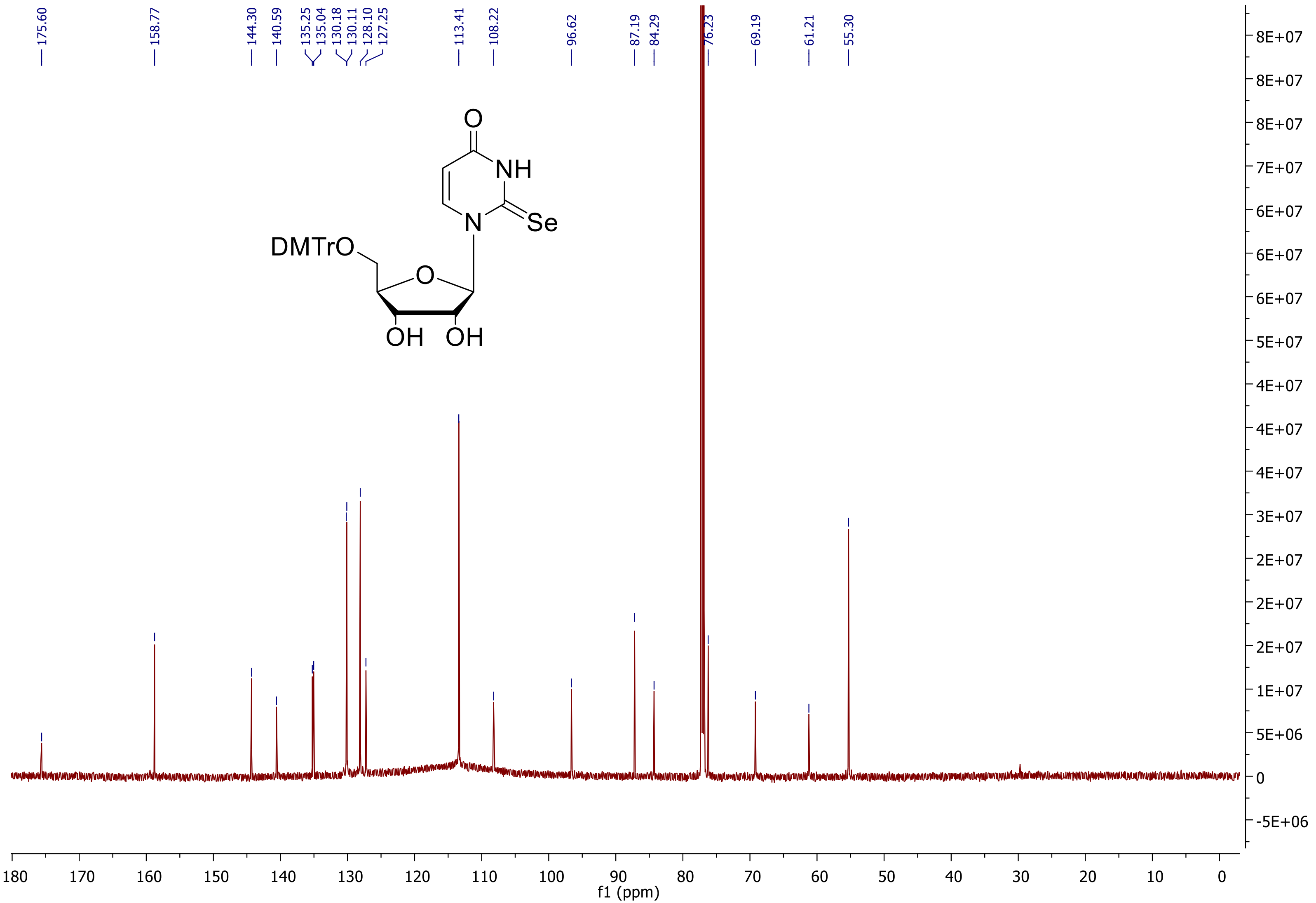

Figure S20. ^{13}C NMR (176 MHz, $CDCl_3$) 5'-*O*-(4,4'-dimethoxytrityl)- 2-selenouridine (**3b**).

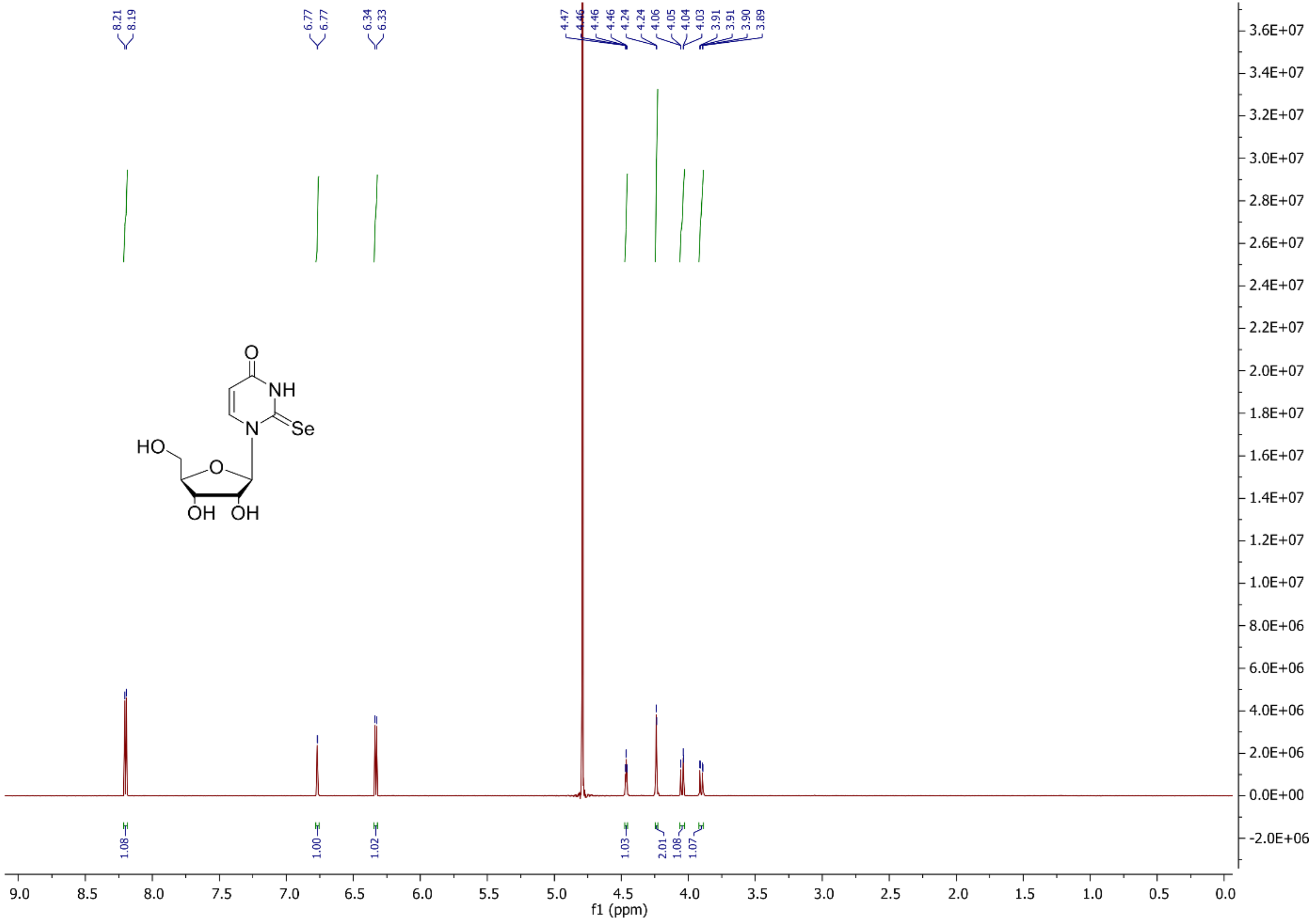

Figure S21. ^{1}H NMR (700 MHz, D_2O) 2-selenouridine **(3)**

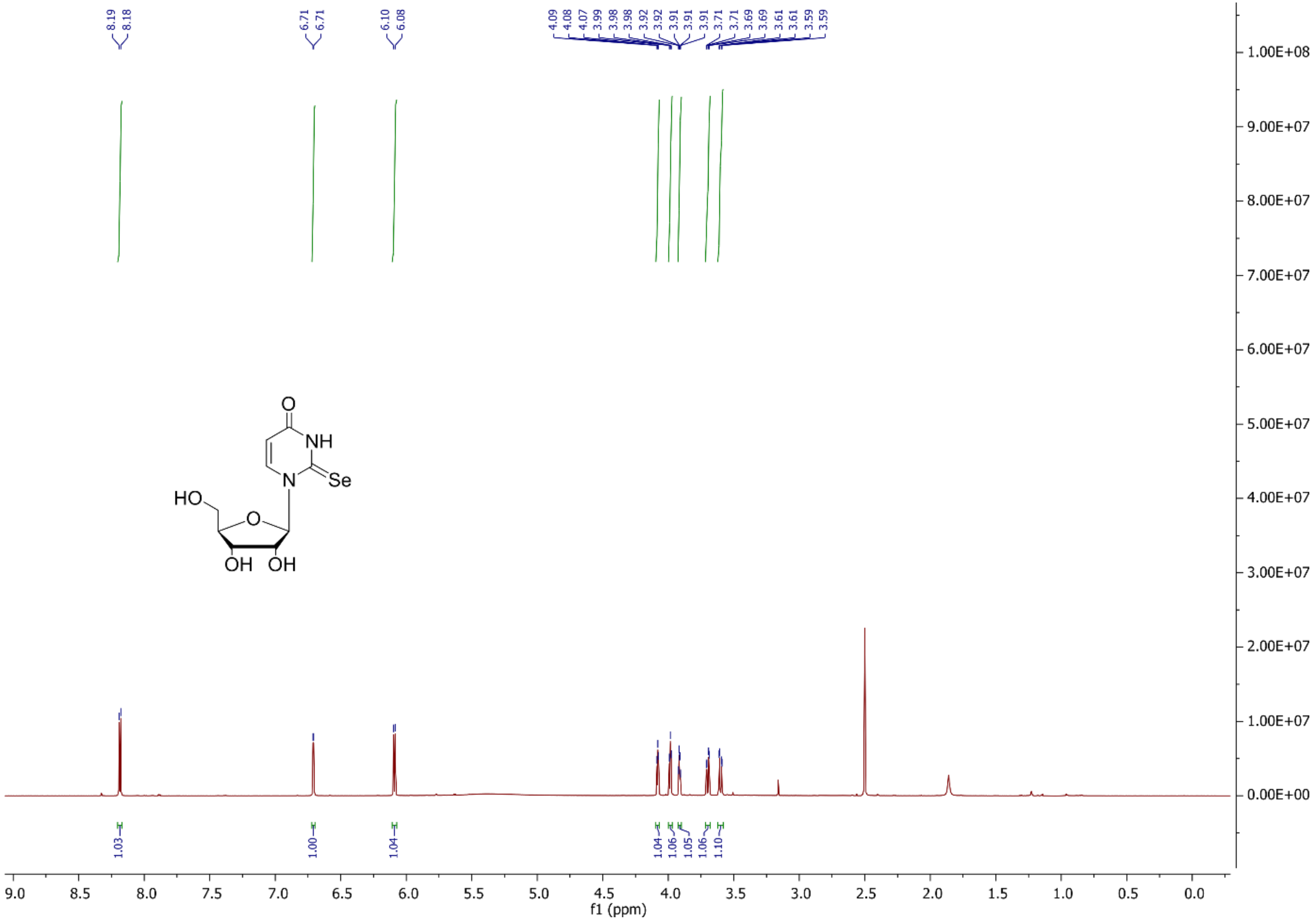

Figure S22. ^{1}H NMR (700 MHz, DMSO-d_6) 2-selenouridine **(3)**

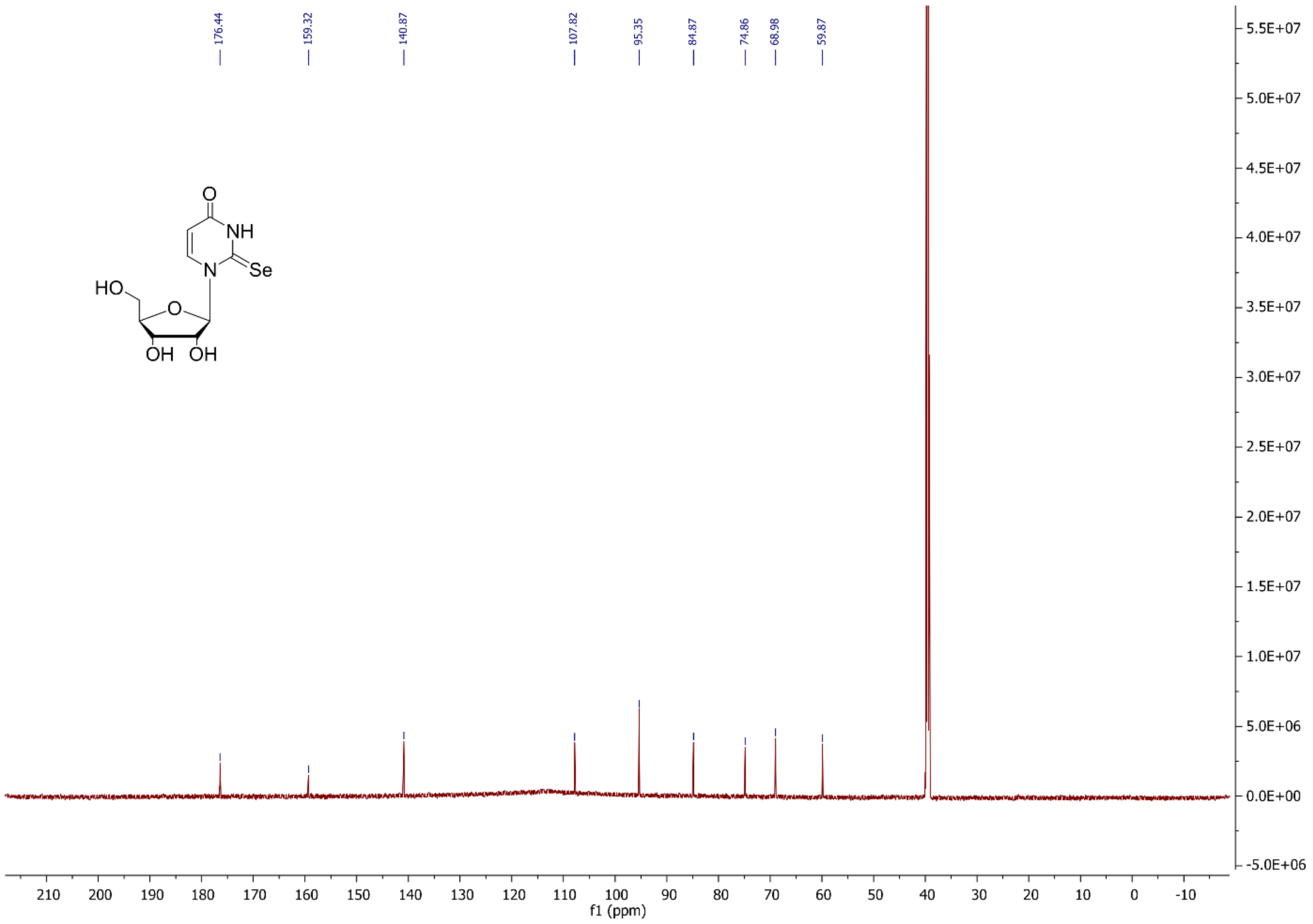

Figure S23. ^{13}C NMR (176 MHz, DMSO-d_6) 2-selenouridine **(3)**

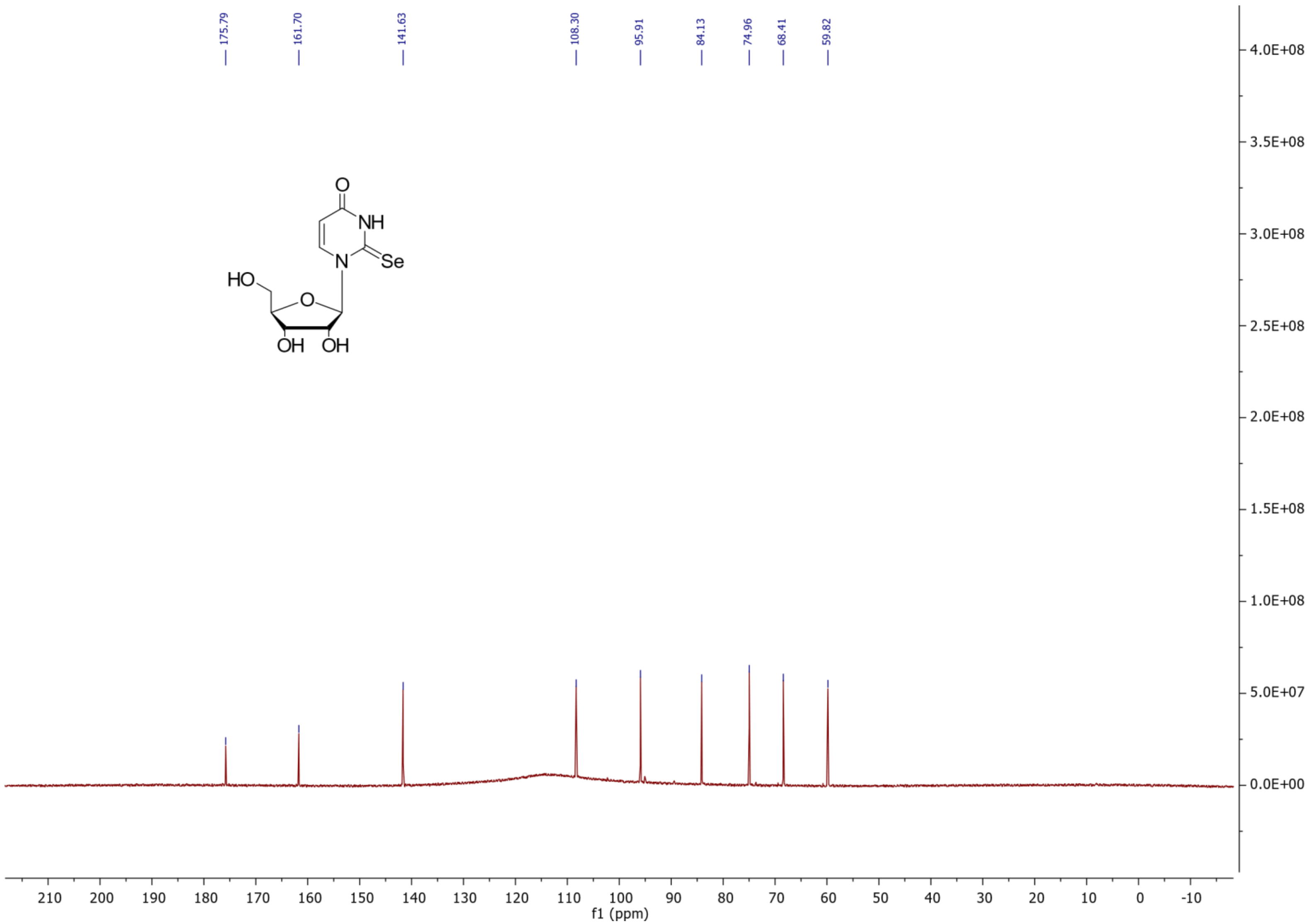

Figure S24. ^{13}C NMR (176 MHz, D_2O) 2-selenouridine **(3)**

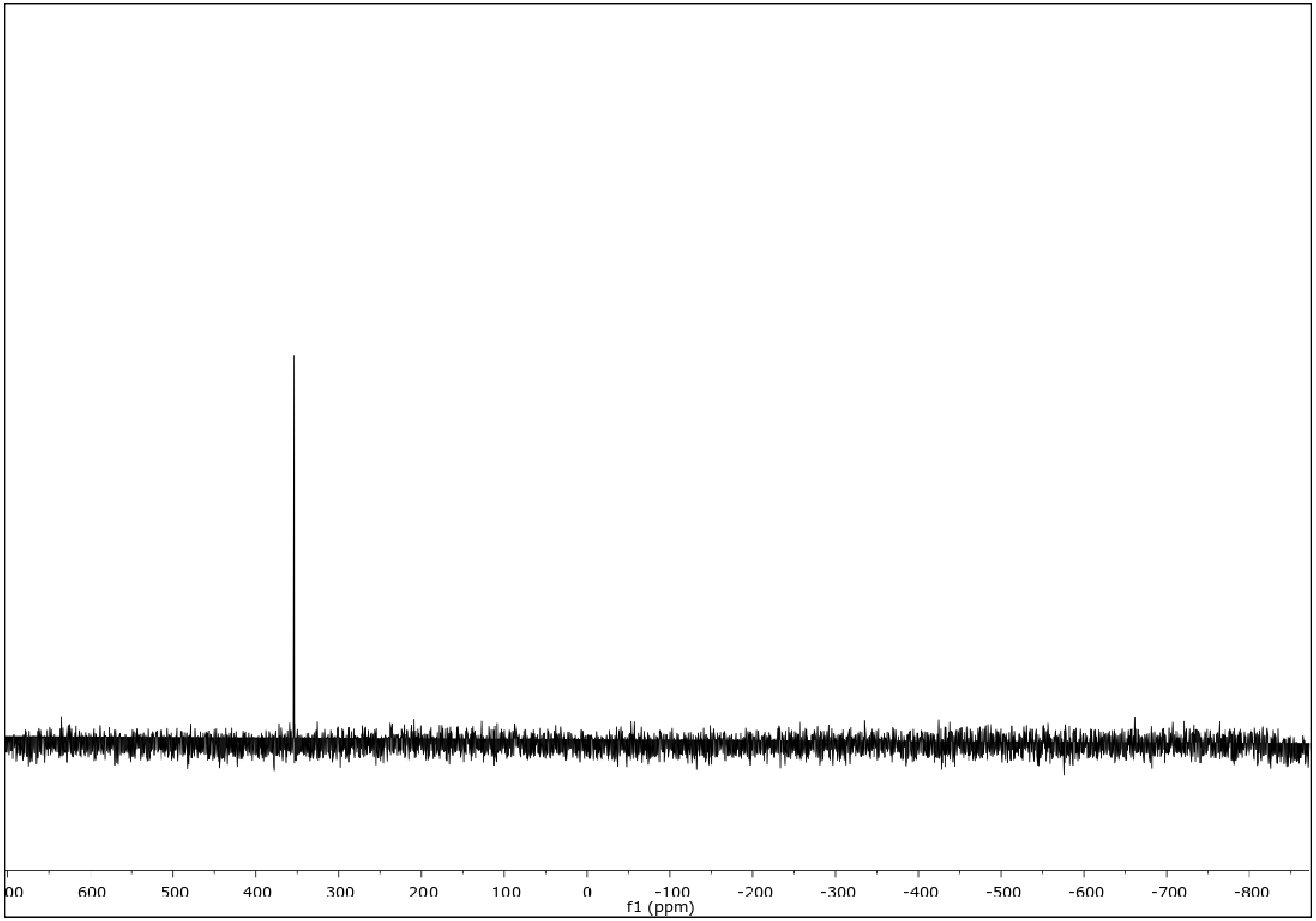

Figure S25. ^{77}Se NMR (134 MHz, DMSO-d_6) 2-selenouridine **(3)**

A

pKa= 6.73 R^2=0.9995

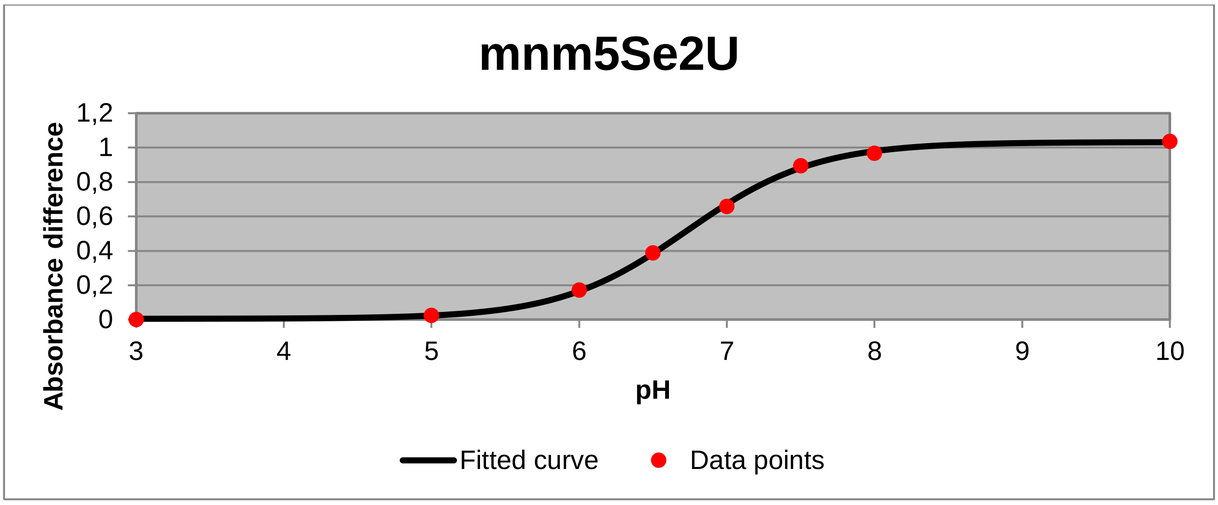

B

pKa= 6.88 R^2=0.9964

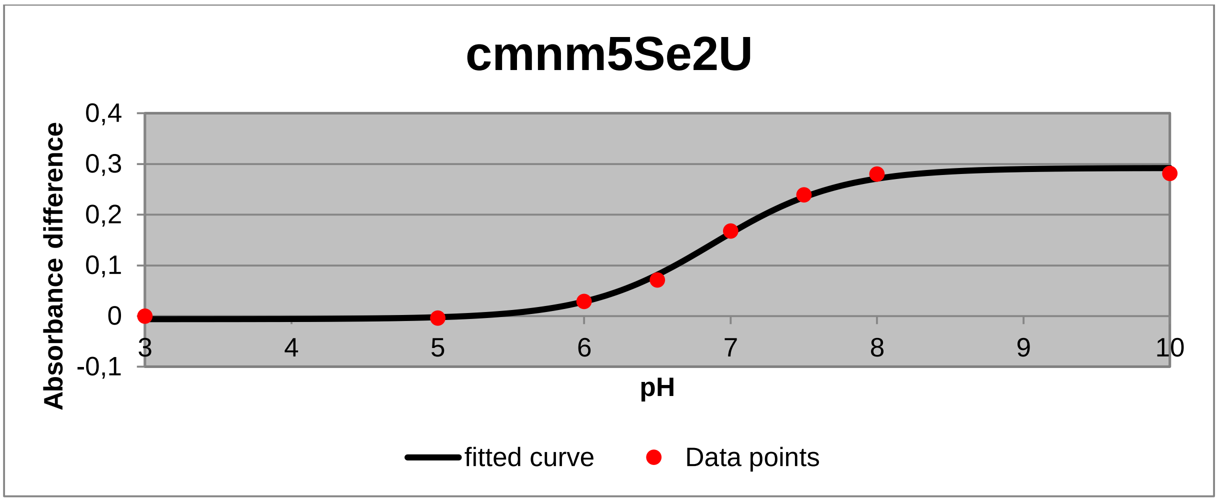

C

pKa= 7.48 R^2=0.9980

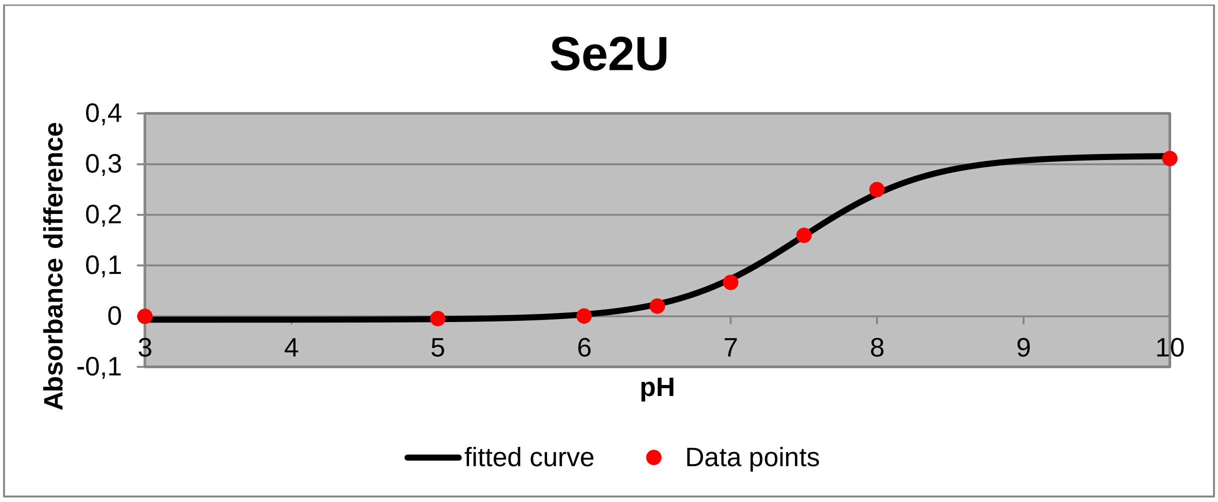

Figure S26. Determination of the pKa values for mnm5Se2U (**1**) (A), cmnm5Se2U (**2**) (B) and Se2U (**3**) (C) by plotting of the total absorbance difference (ΔA) vs pH. The total absorbance difference is the sum of the absolute absorbance difference values at the chosen wavelengths (for mnm5Se2U 239 and 322 nm; for cmnm5Se2U 240 and 322 nm, for Se2U 244 and 314 nm). The pKa values were determined by nonlinear regression according to the equation: y = (a-b*(10^(x-k)))/(1+10^(x-k)), where x=pH and y=ΔA.

3. Crystal structure of Se2U (Tables S1-S6)

Table S1. Hydrogen bonding geometric parameters in crystal packing of Se2U.

	Donor–H	ARU	Acceptor	D – H [Å]	H···A [Å]	D···A [Å]	D – H···A [°]
1	O2'—H2'A		O3'	0.70	2.49	2.7660(1)	106
2	O2'--H2'A	[2656]	O5'	0.70	2.12	2.80 (1)	161
3	O3'--H3'A	[2556]	O5'	0.77	2.18	2.83 (1)	142
4	N3 --H3	[1554]	O3'	0.86	2.16	2.99 (1)	162
5	O5'--H5'	[2645]	Se	0.87	2.50	3.36 (1_	175

Table S2. Selected torsion angles for **Se2U** in comparison with 2-thiouridine (**S2U**)[a] and uridine (**U**). For **U** two structures are described: A and B.[b]

	**Se2U (1)**	**S2U**[a]	**U**[b]	
			**A**	**B**
a) Torsion angels [º]				
χ = O4'-C1'-N1-C2	**-146.5(6)**	-163.0	-161.7	-155.7
$\mathbf{v_0}$ = C4'-O4'-C1'-C2'	**2.2(7)**	6.0	10.5	3.4
$\mathbf{v_1}$ = O4'-C1'-C2'-C3'	**-22.4(6)**	-27.0	-31.4	-27.9
$\mathbf{v_2}$ = C1'-C2'-C3'-C4'	**-33.1(6)**	36.0	39.5	40.4
$\mathbf{v_3}$ = C2'-C3'-C4'-O4'	**-32.4(7)**	-34.0	-34.6	-39.5
$\mathbf{v_4}$ = C3'-C4'-O4'-C1'	**19.3(8)**	18.0	15.3	22.8
**γ** = O5'-C5'-C4'-C3'	**-171.3(6)**	-169.0	45.9	39.6

[a] Hawkinson S.W. "The crystal and molecular structure of 2-thiouridine" *Acta Cryst.B*, 33 **1977**, *33*, 80-85;

[b] Green E.A, Rosenstein R.D., Shiono R, Abraham D.J., Trus B.L., Marsh R.E., "The crystal structure of uridine", *Acta Cryst.B* **1975**, 31, 102-107.

Table S3. Crystal data and structure refinement details for **Se2U**

Identification code	soch1e_a
Empirical formula	$C_9H_{12}N_2O_5Se$
Formula weight	307.17
Temperature/K	100.01(10)
Crystal system	monoclinic
Space group	$P2_1$
a/Å	4.57544(10)
b/Å	12.0960(3)
c/Å	9.5240(2)
α/°	90
β/°	94.413(2)
γ/°	90
Volume/Å^3	525.54(2)
Z	2
ρ_{calc}g/cm^3	1.941
μ/mm^{-1}	5.017
F(000)	308.0
Crystal size/mm^3	? × 0.2014 × 0.0222
Radiation	CuKα (λ = 1.54184)
2Θ range for data collection/°	9.314 to 152.09
Index ranges	-5 ≤ h ≤ 5, -13 ≤ k ≤ 14, -11 ≤ l ≤ 11
Reflections collected	8263
Independent reflections	2018 [R_{int} = 0.0537, R_{sigma} = 0.0354]
Data/restraints/parameters	2018/1/167
Goodness-of-fit on F^2	1.194
Final R indexes [I>=2σ (I)]	R_1 = 0.0373, wR_2 = 0.1028
Final R indexes [all data]	R_1 = 0.0380, wR_2 = 0.1039
Largest diff. peak/hole / e Å^{-3}	0.61/-0.68
Flack parameter	-0.05(3)

Table S4. The deviations of the atoms (Å) from the main pyrimidine ring plane for **Se2U**, **S2U** and **U**. For **U** two structures are described: A and B.[b]

atoms	**Se2U**	**S2U**	**U** A	B
N1	0.002	-0.022	0.003	-0.007
C2	0.030	0.003	0.016	0.004
N3	0.043	0.023	-0.027	0.000
C4	0.023	-0.029	0.017	-0.002
C5	0.008	-0.011	0.002	-0.001
C6	0.020	0.014	-0.011	0.006
Se2	0.234			
S2		0.012	-	-
O2		-	0.073	0.015
O4	0.056	-0.110	0.057	0.012
r.m.s.	Δ0.004	Δ 0.004	Δ 0.005	Δ 0.006

Table S5. Comparison of the bond lengths (Å) for **Se2U, S2U**[a] and **U**. For **U** two structures are described: A and B.[b]

bonds	**Se2U**	**S2U**	**U**	
			A	B
C4'-O4'	1.467(9)	1.453(3)	1.453	1.454
O4'-C1'	1.410(8)	1.411(3)	1.414	1.412
C1'-N1	1.493(8)	1.500(3)	1.497	1.483
N1-C2	1.360(9)	1.368(3)	1.376	1.366
C2-Se2	1.851(8)			
C2-S2		1.677(3)	-	-
C2-O2		-	1.227	1.217
C4-O4	1.218(1)	1.228(3)		
C2-N3	1.349(9)	1.360(3)	1.371	1.369
N3-C4	1.388(10)	1.388(3)	1.387	1.374
C4-C5	1.442(10)	1.435(3)	1.423	1.419
C5-C6	1.345(12)	1.337(3)	1.333	1.335
C6-N1	1.379(10)	1.381(3)	1.369	1.369
C1'- C2'	1.529(9)	1.528(3)	1.528	1.533
C2'- C3'	1.536(9)	1.526(3)	1.527	1.525
C3'-C4'	1.527(11)	1.522(3)	1.515	1.522
C4'-C5'	1.520(11)	1.498(3)	1.512	1.500
C2'-O2'	1.410(8)	1.412(3)	1.418	1.418

[a] Hawkinson S.W. "The crystal and molecular structure of 2-thiouridine" *Acta Cryst.B*, 33 **1977**, *33*, 80-85;

[b] Green E.A, Rosenstein R.D., Shiono R, Abraham D.J., Trus B.L., Marsh R.E., "The crystal structure of uridine", *Acta Cryst.B* **1975**, 31, 102-107.

Table S6. Comparison of the bond angles of **Se2U, S2U**[a] and **U**. For **U** two structures are described: A and B.[b]

Bond angles	**Se2U**	**S2U**	**U**	
			A	B
C2-N1-C6	120.4(7)	120.8	120.6	120.5
C1'-O4'-C4'	110.6(5)	109.8	108.5	109.6
C2-N1-C1'	120.2(5)	117.8	117.4	117.0
C6-N1-C1'	119.3(6)	121.3	122.0	122.4
C2-N3-C4	126.9(6)	126.6	126.4	127.2
N3-C2-N1	116.4(6)	116.0	115.5	115.3
N3-C2-O2 (Se, S)	121.0(5)	120.7	122.8	122.2
N1-C2-O2 (Se,S)	122.6(6)	123.4	121.7	122.6
O4-C4-N3	120.1(7)	119.2	120.4	118.8
O4-C4-C5	125.8(7)	126.4	125.6	127.2
N3-C4-C5	114.0(6)	114.4	114.3	114.0
C6-C5-C4	118.9(7)	119.5	120.0	120.0
N1-C6-C5	122.9(8)	122.6	120.6	120.5
O4'-C1'-N1	109.0(5)	109.3	108.4	109.0
O4'-C1'-C2'	107.9(5)	107.1	106.8	107.4
N1-C1'-C2'	115.3(7)	110.6	111.8	113.6
O2'-C2'-C3'	111.1(5)	109.6	108.6	107.6
O2'-C2'-C1'	105.2(5)	108.2	110.0	108.5
C3'-C2'-C1'	102.3(5)	100.8	100.4	100.6
O3'-C3'-C4'	110.7(6)	114.6	113.7	114.4
O3'-C3'-C2'	113.6(5)	114.4	113.1	117.2
C4'-C3'-C2'	103.2(7)	101.4	102.1	117.2
O4'-C4'-C5'	108.5(7)	111.6	111.1	101.4
O4'-C4'-C3'	104.1(6)	103.5	113.7	114.4
C5'-C4'-C3'	113.6(6)	113.7	115.6	117.0
O5'-C5'-C4'	113.8(6)	111.3	111.5	112.6

[a] Hawkinson S.W. "The crystal and molecular structure of 2-thiouridine" *Acta Cryst.B*, 33 **1977**, *33*, 80-85;

[b] Green E.A, Rosenstein R.D., Shiono R, Abraham D.J., Trus B.L., Marsh R.E., "The crystal structure of uridine", *Acta Cryst.B* **1975**, 31, 102-107.

4. Results of DFT calculations (Figures S27-S32, Tables S7-S8)

Structural analysis of m1Se2Ura and m1mnm5Se2Ura

The theoretically determined lengths of all covalent bonds in the X2-C2-N3-C4-O4 bonding region of the tautomers of the m1Se2Ura and m1mnm5Se2Ura models in water are shown in Fig. S28. Moreover, the C2-X bond lengths, where X=O, S, or Se, are compared in Table S7. Our calculations demonstrate that all C2-X bonds are quite sensitive to tautomerization and vary by approximately 0.1 Å upon conversion from the K form to the E2 form. Generally, the C2-X bond is the shortest in the K tautomer (which corresponds to the strongest double bond character) and the longest in the E2 tautomer (corresponding to the strongest single bond character). The C-Se bond in mnm5Se2U is slightly longer in each tautomeric form than in unsubstituted Se2U (by 0.005-0.011 Å), but between the lengths for a pure C=Se bond (1.74 Å) and a pure C2-Se single bond (1.94 Å).[d]

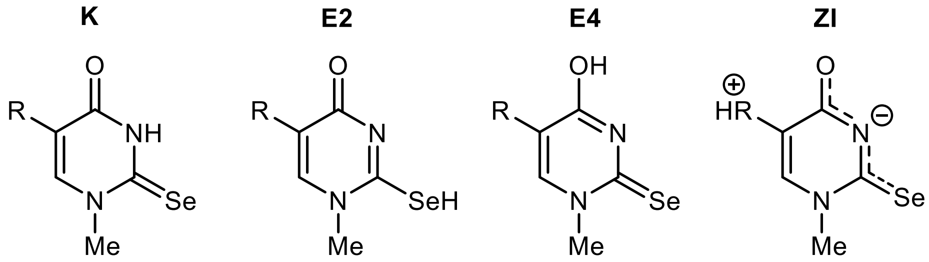

Figure S27. The possible keto-enol tautomers of 1-methyl-5-substituted 2-selenouracils (R=H or CH_2NHCH_3) (diketo- **K**, 4-keto-2-enol- **E2**, 2-keto-4-enol- **E4** and zwitterionic- **ZI**) in water.

[d] Landry,V.K, Minoura,M., Pang,K., Buccella,D., Kelly,B.V. and Parkin,G. (2006) Synthesis and structural characterization of 1-mesityl-1,3-dihydro-imidazole-2-selone and bis(1-mesitylimidazol-2-yl)diselenide: experimental evidence that the selone is more stable than the selenol tautomer. J. Am. Chem. Soc., 128, 12490-12497.

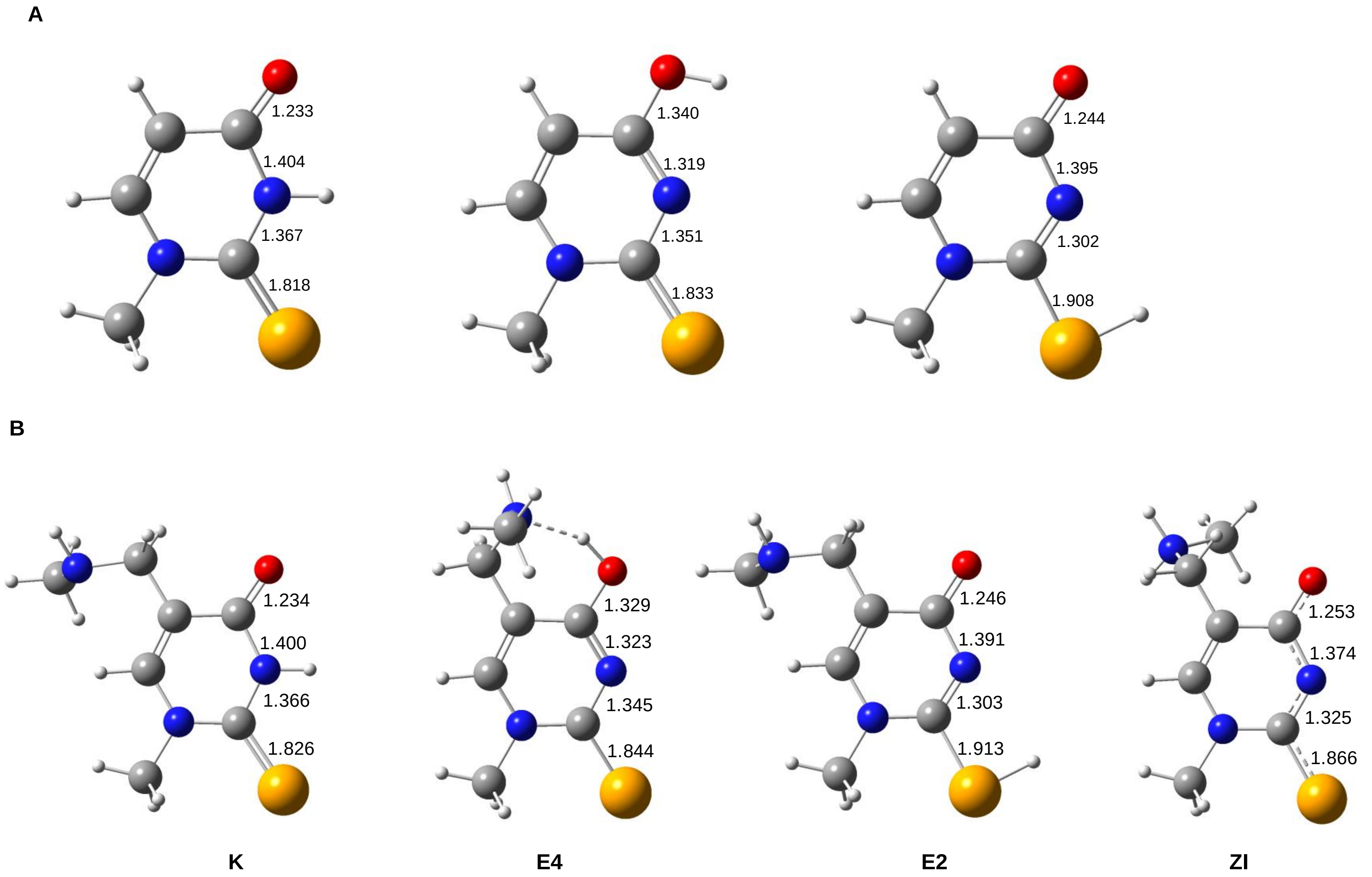

Figure S28. The lengths of all covalent bonds in the bonding region X2-C2-N3-C-O4 within tautomers of m1Se2Ura and m1mnm5Se2Ura models optimized in water.

Table S7. Comparison of C2-X bond lengths and ESP atomic charges on C2 and X for X2Ura tautomers in water

		Tautomer	X=O	X=S	X=Se
C2-X bond length (Å)	X2Ura	**K**	1.230	1.684	1.818
		**E2**	1.341	1.783	1.908
		**E4**	1.237	1.699	1.833
	mnm5X2Ura	**K**	1.232	1.687	1.826
		**E2**	1.343	1.793	1.913
		**E4**	1.241	1.706	1.844
		**ZI**	1.250	1.726	1.866
ESP charge on C2/X	X2Ura	**K**	0.795/-0.656	-0.024/-0.405	-0.511/-0.189
		**E2**	0.838/-0.590	0.337/-0.188	-0.052/0.059
		**E4**	1.027/-0.738	0.497/-0.565	0.265/-0.367
	mnm5X2Ura	**K**	0.702/-0.650	-0.067/-0.433	-0.368/-0.249
		**E2**	0.745/-0.581	0.346/-0.206	0.001/0.042
		**E4**	0.909/-0.732	0.464/-0.508	0.240/-0.407
		**ZI**	0.994/-0.789	0.626/-0.723	0.419/-0.520

Table S8. The relative Gibbs free energies (ΔG_{rel}, kcal/mol) of the E2, E4 and ZI tautomers of m1R5X2Ura (X = O, S, Se, R5 = H, mnm) models in water at 25°C (298 K), calculated at CPCM/B3LYP-GD3/6-311+G(3df,2p)//B3LYP-GD3/6-31+G(d).

ΔG_{rel} in water (kcal/mol) [a]									
Tautomeric form	**E2**			**E4**			**ZI**		
X	O	S	Se	O	S	Se	O	S	Se
R5=H[b]	16.2	14.4	13.2	10.8	10.2	10.1	-	-	-
R5=CH_2NHCH_3[b]	15.7	12.1	13.5	6.3	4.3	4.7	6.3	4.8	5.9

[a] Values for 5-substituted 1-methyl-uracils and 2-thiouracils are taken from ref. 3.
[b] The free energies of the most stable K tautomers of m1Se2Ura and m1mnm5Se2Ura were taken as zero (reference values).

Atomic charge distribution (ESP, Merz-Kollman scheme) in water

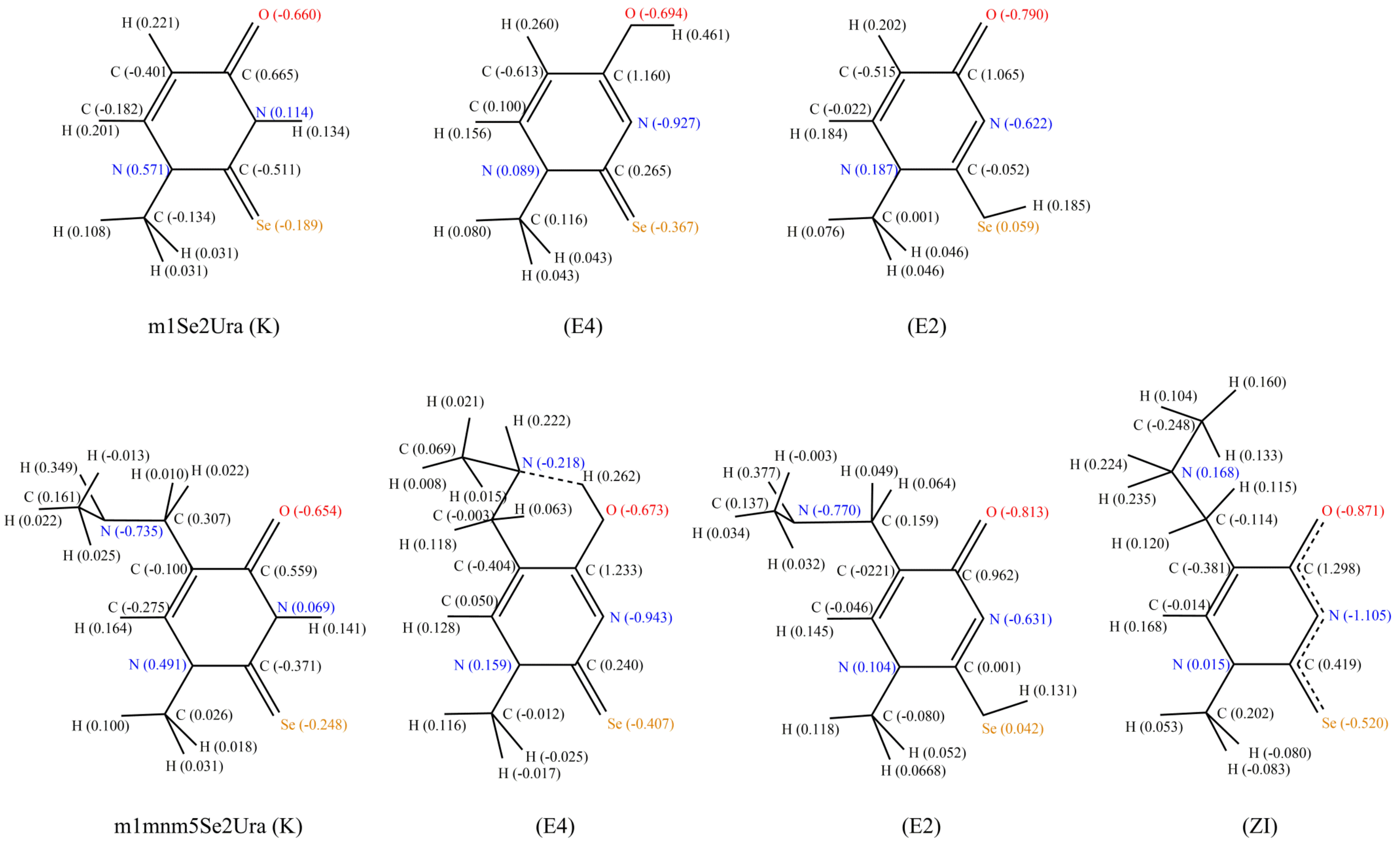

Figure S29. ESP atomic charge distribution (B3LYP-GD3/6-311++G(3df,2p)//B3LYP-GD3/6-31+G(d)) for m1Se2Ura and m1mnm5Se2Ura in water

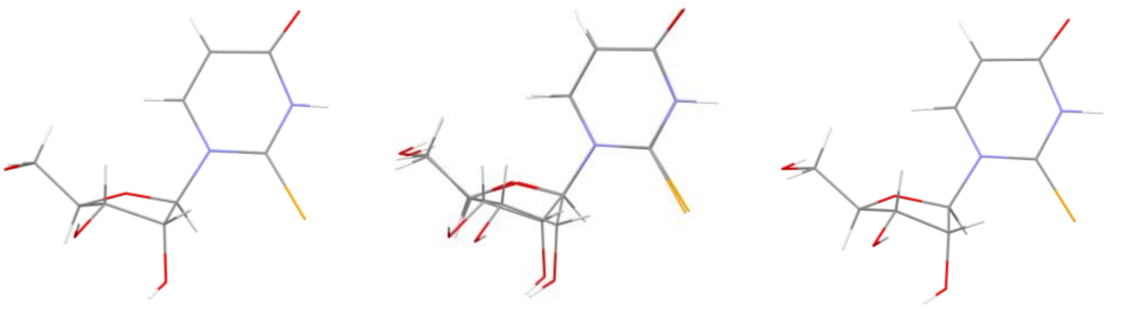

Crystal structure r(C-Se) = 1.851 Å | overlap of crystal and DFT | DFT structure (in H_2O) r(C-Se) = 1.820 Å

Figure S30. Overlapping of crystal and DFT(H_2O) structures of Se2Ura.

Electrostatic potential map

An electrostatic potential energy map illustrating the charge distributions in the most abundant mnm5Se2U base was analysed by quantum chemical calculations carried out for three of the most stable tautomeric forms of the m1mnm5Se2Ura model protonated at the amino alkyl residue. As shown in Fig. 5, the 2,4-diketo tautomer (K) contains electron-rich regions in the vicinity of both Se2 and O4 atoms, while N3 is shielded by the hydrogen atom. In the E4 tautomer, the electron-rich region is noted at the Se2···N3 location. In the zwitterionic tautomeric structure, the electron-deficient region is located in the vicinity of the ammonium cation at the side chain, while the electron-rich region is dispersed over the Se2···N3···O4 edge. The electrostatic potential maps obtained for the three tautomeric forms of the 2-selenouracil model are consistent with those of the corresponding 2-oxo- and 2-thio-uracils.[e]

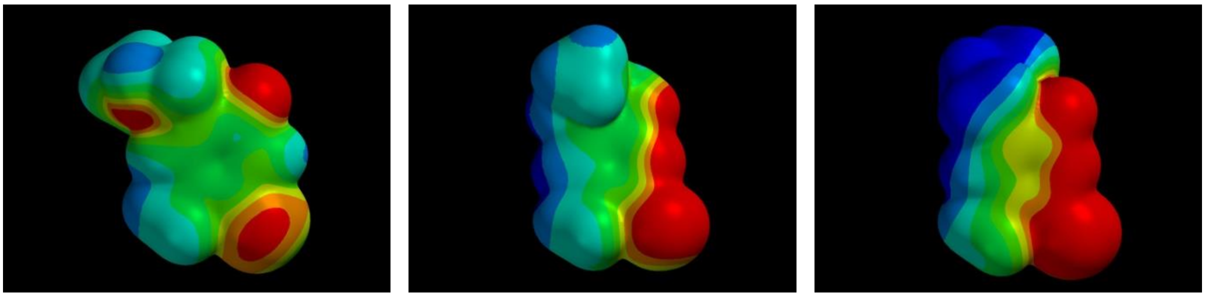

(K) (E4) (ZI)

Figure S31. Electrostatic potential energy map illustrating the charge distributions in the mnm5Se2U base analysed by quantum chemical calculations carried out for three the most stable tautomeric forms of the m1mnm5Se2Ura model K, E4 and E2, protonated at the amino alkyl residue.

[e] Sochacka,E., Lodyga-Chruscinska,E., Pawlak,J., Cypryk,M., Bartos,P., Ebenryter-Olbinska,K., Leszczynska,G., and Nawrot,B. (2017) C5-substituents of uridines and 2-thiouridines present at the wobble position of tRNA determine the formation of their keto-enol or zwitterionic forms - a factor important for accuracy of reading of guanosine at the 3´-end of the mRNA codons. Nucleic Acids Res., 45, 4825-4836.

Table 9. B3LYP-GD3/6-31+G(d) optimized geometries (Cartesian coordinates, in water solution) for R5Se2Ura tautomers (R=H, mnm) and their base pairs with me9Gua.

m1Se2Ura (K)

N	-1.12977900	-0.57271200	0.00000000
C	-1.04072900	-1.94919800	0.00000000
H	-1.99071500	-2.46955900	0.00000000
C	0.13549500	-2.62137600	0.00000000
H	0.16599700	-3.70288000	0.00000000
C	1.37237200	-1.87717200	0.00000000
O	2.51509200	-2.34010300	0.00000000
N	1.17990200	-0.48669700	0.00000000
H	2.02579500	0.07617200	0.00000000
C	0.00000000	0.20348800	0.00000000
C	-2.46139900	0.05836000	0.00000000
H	-2.57565100	0.67915600	0.89078700
H	-2.57565100	0.67915600	-0.89078700
H	-3.21289000	-0.73019800	0.00000000
Se	-0.01008600	2.02118300	0.00000000

m1Se2Ura (E4)

N	-1.01579600	-0.75685300	0.00000000
C	-0.71002800	-2.08428300	0.00000000
H	-1.54918900	-2.76904800	0.00000000
C	0.58132300	-2.52812000	0.00000000
H	0.83031300	-3.58073000	0.00000000
C	1.56397400	-1.51259000	0.00000000
O	2.85163700	-1.88200300	0.00000000
N	1.28217000	-0.22380600	0.00000000
C	0.00000000	0.20037700	0.00000000
C	-2.43207500	-0.35167300	0.00000000
H	-2.64126500	0.24595200	0.88921300
H	-2.64126500	0.24595200	-0.88921300
H	-3.05078200	-1.24892400	0.00000000
H	3.40098100	-1.07354300	0.00000000
Se	-0.38369600	1.99290400	0.00000000

m1Se2Ura (E2)

N	-0.39343600	-1.19412600	0.00000000
C	0.59904800	-2.15052500	0.00000000
H	0.25816300	-3.17917400	0.00000000
C	1.91011500	-1.80991500	0.00000000
H	2.68084800	-2.57143200	0.00000000
C	2.28299200	-0.40592100	0.00000000
O	3.46527700	-0.01803900	0.00000000
N	1.23859800	0.52079500	0.00000000
C	0.00000000	0.12072000	0.00000000
C	-1.81009100	-1.59344300	0.00000000
H	-2.30969300	-1.21837300	0.89752700
H	-2.30969300	-1.21837300	-0.89752700
H	-1.86478000	-2.68142600	0.00000000

H	-0.42412800	2.52654200	0.00000000
Se	-1.39886600	1.41865800	0.00000000

m1mnm5Se2Ura (K)

N	-0.36291300	-0.96274100	-0.22990600
C	1.00395400	-0.78811800	-0.34503400
H	1.57998100	-1.68920100	-0.51517200
C	1.62200800	0.41492900	-0.25936200
C	0.80124600	1.59036900	-0.04577400
O	1.20361000	2.75394200	0.03977000
N	-0.56742500	1.31384700	0.05832100
C	-1.19131400	0.10123100	-0.02238500
C	-0.91899800	-2.32280400	-0.33244500
H	-1.43740900	-2.58044300	0.59339600
H	-1.62307300	-2.37187400	-1.16542400
H	-0.09613000	-3.01626200	-0.50162400
C	3.11663200	0.58837100	-0.40318100
H	3.45554000	1.29847500	0.37183400
H	3.31939800	1.07125300	-1.36765000
N	3.83655300	-0.68274200	-0.35725600
H	4.72577100	-0.57297700	-0.83703200
C	4.07734800	-1.17335900	1.00419200
H	3.12395500	-1.41377100	1.48724900
H	4.67194300	-2.09085300	0.95708700
H	4.60431900	-0.44219400	1.64156700
H	-1.16931400	2.11857800	0.20830900
Se	-3.00566600	-0.01425100	0.14515100

m1mnm5Se2Ura (E4)

N	-0.72028400	1.18346500	-0.05880300
C	0.60300400	1.45619200	-0.24414600
H	0.87587700	2.50524100	-0.26200200
C	1.53114400	0.47038100	-0.41344400
C	1.01928000	-0.86414000	-0.32945000
O	1.84356300	-1.90445300	-0.40110600
N	-0.26720200	-1.12819900	-0.17268800
C	-1.16624400	-0.13519900	-0.04942400
C	-1.66015400	2.30375900	0.11044800
H	-2.39600500	2.29033800	-0.69590100
H	-2.17814600	2.20597700	1.06632100
H	-1.09582200	3.23616000	0.08599000
C	2.98478700	0.74956100	-0.72469400
H	3.24652000	1.77612700	-0.43000700
H	3.14045700	0.66731000	-1.80718400
N	3.85211400	-0.25876400	-0.08241500
H	4.74647800	-0.29071000	-0.56675300
C	4.06662100	-0.02925700	1.35632300
H	3.10779600	-0.09644800	1.87993200
H	4.72800500	-0.80718800	1.74639100
H	4.50685100	0.95759400	1.55971600
H	2.79794600	-1.52450800	-0.33750900
Se	-2.95739600	-0.52845800	0.14510300

m1mnm5Se2Ura (E2)

N	-0.34678000	-0.93993600	-0.23310200
C	1.02174100	-0.79457500	-0.34255800
H	1.58574100	-1.70323100	-0.51404900
C	1.62497000	0.41533700	-0.24457500
C	0.78571400	1.59207100	-0.04005400
O	1.25832900	2.74309700	0.02634200
N	-0.58571100	1.38860300	0.07433200
C	-1.08569800	0.18960900	-0.02128300
C	-0.95781200	-2.27381300	-0.33766400
H	-1.47287900	-2.52950200	0.59277700
H	-1.65626500	-2.30642700	-1.17859000
H	-0.16791300	-3.00357100	-0.51187000
H	-3.17457800	1.41217800	0.32949400
C	3.11885700	0.59765000	-0.37114500
H	3.44805700	1.28294200	0.43023600
H	3.32486700	1.11879500	-1.31501100
N	3.85569800	-0.66691000	-0.36588300
H	4.74746800	-0.52642500	-0.83306200
C	4.09783900	-1.19845000	0.97981800
H	3.14635300	-1.46836800	1.45072500
H	4.70698100	-2.10487500	0.90672300
H	4.61091700	-0.48059600	1.64372400
Se	-2.97827900	-0.03749900	0.13898600

m1mnm5Se2Ura (ZI)

N	0.66020700	1.15543900	-0.11752900
C	-0.66790600	1.36655500	-0.33960400
H	-0.98304800	2.40490200	-0.36495900
C	-1.54651700	0.34259900	-0.52341200
C	-1.02286500	-1.02218900	-0.50751500
O	-1.77098300	-2.00939700	-0.69440500
N	0.32328100	-1.18331800	-0.28191100
C	1.14311100	-0.16064200	-0.08680000
C	1.54310100	2.31573600	0.07272700
H	2.01916200	2.26704300	1.05387700
H	2.31728900	2.32674500	-0.69692600
H	0.94054700	3.22254300	0.00120700
C	-2.99253900	0.59967900	-0.79153600
H	-3.41926300	-0.18348200	-1.42019900
H	-3.16216800	1.57428900	-1.25433200
N	-3.83450300	0.60865200	0.48482300
H	-4.79701800	0.85409800	0.22961400
C	-3.84920700	-0.67258200	1.26869200
H	-2.84102500	-0.87048400	1.62731200
H	-4.53715600	-0.54766400	2.10536600
H	-4.17264600	-1.47399800	0.60708600
H	-3.50614700	1.36870300	1.09016200
Se	2.95951100	-0.45729000	0.21835400

m1Se2Ura-G (U_K-G)

N	-4.20973600	-0.11553300	0.09572600
C	-4.89519800	1.08180900	0.19855600
H	-5.97178500	0.98555600	0.28382600
C	-4.28480200	2.28426800	0.19563300
H	-4.84052900	3.20926400	0.27805900
C	-2.83702900	2.35232800	0.07599500
O	-2.17203800	3.37344900	0.06065000
N	-2.22054700	1.07759900	-0.02830200
H	-1.18416700	1.10862700	-0.12134800
C	-2.83532200	-0.13078700	-0.01943800
C	-4.97397700	-1.37151600	0.11053100
H	-4.81197800	-1.92401400	-0.81795100
H	-4.65900900	-1.99580400	0.94993600
H	-6.03182600	-1.12385900	0.21198800
N	5.09016900	0.53578900	0.06125200
C	4.86327000	1.89866700	-0.06733300
H	5.68753900	2.60069700	-0.08282100
N	3.59550100	2.21061000	-0.16388800
C	2.93867400	0.99736700	-0.09809400
C	1.54387100	0.67496100	-0.14475100
O	0.56176000	1.41802500	-0.24606000
N	1.34766700	-0.72289100	-0.05484100
H	0.36259100	-1.01511400	-0.11701600
C	2.32689300	-1.66700300	0.09513000
N	1.91382300	-2.97732700	0.13437500
H	0.97275200	-3.16981700	0.45444300
H	2.61793600	-3.62940900	0.45494700
N	3.61129800	-1.38387300	0.15540700
C	3.85318700	-0.05377000	0.04254200
C	6.36345100	-0.15191800	0.19452700
H	6.40973700	-0.68406800	1.14951200
H	6.49013200	-0.87343800	-0.61794900
H	7.16694100	0.58699300	0.15200400
Se	-1.92945400	-1.71366000	-0.16447800

m1Se2Ura-G (U_{E4}-G)

N	4.19246000	0.33223900	-0.00004200
C	4.45447500	1.67203500	-0.00016600
H	5.50253000	1.94991800	-0.00022800
C	3.45858700	2.59490500	-0.00021200
H	3.65121800	3.65975300	-0.00030900
C	2.12374600	2.08726300	-0.00010100
O	1.15768800	2.96898900	-0.00010700
N	1.86484200	0.77990900	-0.00000900
C	2.86997600	-0.12593400	0.00002400
N	-5.14947300	-0.26216100	-0.00002400
C	-5.43775800	1.09683900	0.00011400
H	-6.46232900	1.44757300	0.00015800
N	-4.37544900	1.85922700	0.00018300
C	-3.31803100	0.96734500	0.00008300
C	-1.90990300	1.17870800	0.00011900
O	-1.29875600	2.26478500	0.00027700

N	-1.19261200	-0.03196700	-0.00001500
H	-0.16936000	0.06427400	-0.00001000
C	-1.75915100	-1.29116000	-0.00013900
N	-0.91129900	-2.34002000	-0.00030000
H	0.10442800	-2.22280700	-0.00008800
H	-1.31237000	-3.26598800	-0.00019400
N	-3.06692200	-1.49862200	-0.00015100
C	-3.78145700	-0.35424200	-0.00004600
C	5.31589900	-0.61447000	0.00000000
H	5.26639500	-1.25204500	0.88567300
H	5.26633200	-1.25219800	-0.88555900
H	6.24578600	-0.04204600	-0.00008200
C	-6.08608200	-1.37383200	-0.00014700
H	-7.10256500	-0.97365100	0.00001500
H	-5.94185000	-1.99410800	-0.88985800
H	-5.94169200	-1.99442600	0.88931400
H	0.21316800	2.56773800	0.00003900
Se	2.54988900	-1.92521100	0.00015000

m1mnm5Se2Ura-G (U_K-G)

N	-3.32431500	1.21999900	-0.16430300
C	-4.22421000	0.17522800	-0.25839100
H	-5.26798500	0.45549600	-0.32585000
C	-3.85811000	-1.12763500	-0.27466900
C	-2.44497100	-1.44275600	-0.19952800
O	-1.96753100	-2.58102400	-0.21519500
N	-1.60363200	-0.32385200	-0.11176800
H	-0.59106500	-0.53875700	-0.06474500
C	-1.98266000	0.98159800	-0.09022700
N	5.70071900	-1.30601000	0.04736100
C	5.18497500	-2.58723500	0.01948900
H	5.83894200	-3.44939200	0.01517900
N	3.87081500	-2.62260200	0.00036400
C	3.49531600	-1.28999700	0.01616600
C	2.20870800	-0.67780700	0.00752400
O	1.08749600	-1.22300400	-0.01780900
N	2.30200300	0.72198700	0.03293600
H	1.39543800	1.21239200	0.04484700
C	3.46893500	1.44692000	0.05538600
N	3.34950400	2.80467200	0.12976000
H	2.49762500	3.24085400	-0.20105300
H	4.19464200	3.32640400	-0.06803200
N	4.66832000	0.89136000	0.06029400
C	4.62261100	-0.46054600	0.04556500
C	-3.84642100	2.59819700	-0.14415000
H	-3.55846800	3.09085100	0.78662200
H	-3.44585400	3.15976900	-0.99032200
H	-4.93235800	2.55069800	-0.21480500
C	7.10507800	-0.91408600	0.07023000
H	7.34102000	-0.31685500	-0.81437400
H	7.31472200	-0.32855500	0.96904800
H	7.71850500	-1.81625800	0.07344100

C	-4.84865400	-2.26173100	-0.39721600
H	-4.73985400	-2.71041700	-1.39287000
H	-4.56237800	-3.04694000	0.32461200
N	-6.23251800	-1.82116900	-0.23120500
H	-6.84486300	-2.48187200	-0.70187300
C	-6.64450600	-1.69466700	1.17123100
H	-7.70387600	-1.42337100	1.21294600
H	-6.49310500	-2.62089100	1.75246400
H	-6.07256800	-0.89659100	1.65705600
Se	-0.75238400	2.34123700	0.03909500

m1mnm5Se2Ura-G (U_{E4}-G)

N	-3.34304600	1.06309400	-0.06604300
C	-3.92585600	-0.17035700	0.00469900
H	-4.99137100	-0.19661200	0.19510500
C	-3.21936200	-1.32490500	-0.17008500
C	-1.83131900	-1.14575400	-0.42500800
O	-1.09388600	-2.22235900	-0.63192200
N	-1.26081900	0.05581800	-0.47215700
C	-1.98925200	1.17795200	-0.31916600
N	5.56095000	-0.57671700	0.34382900
C	5.64769300	-1.73918200	-0.39881400
H	6.59754600	-2.23846600	-0.53917500
N	4.49438500	-2.13812200	-0.88658800
C	3.59266800	-1.18332400	-0.44589400
C	2.18887700	-1.06550200	-0.62166900
O	1.42270500	-1.84675300	-1.23825500
N	1.66790800	0.07540300	-0.00335500
H	0.65306900	0.21643700	-0.13749200
C	2.39645100	0.97530800	0.74404800
N	1.69790100	1.97869500	1.34141100
H	0.82896300	2.28689300	0.89475400
H	2.26955000	2.72722700	1.71531500
N	3.70339700	0.86905500	0.93222700
C	4.24170900	-0.20826900	0.32297000
C	-4.18412100	2.25763500	0.12484000
H	-3.79781400	2.84671700	0.95856800
H	-4.16981300	2.86644200	-0.78116700
H	-5.20179500	1.93157500	0.33830700
C	6.64248000	0.12590700	1.02448900
H	7.57382400	-0.41452000	0.84938200
H	6.73229800	1.14220500	0.63243600
H	6.44266000	0.16827800	2.09832500
H	-0.13249700	-1.98817800	-0.86293700
C	-3.85219400	-2.69848900	-0.13427700
H	-3.20043300	-3.36734600	0.45446800
H	-3.86280000	-3.10070700	-1.15501000
N	-5.22379000	-2.66369100	0.36908000
H	-5.72508800	-3.47212600	0.01109200
C	-5.31255100	-2.64609900	1.83354100
H	-6.36490300	-2.68368200	2.13108900
H	-4.77914200	-3.48666100	2.31013000

H	-4.88574800	-1.71467400	2.22171400
Se	-1.17842900	2.83105300	-0.48589300

m1mnm5Se2Ura-G (U_{ZI}-G)

N	4.015104226	-0.5664432766	-0.1782072425
C	4.1353269066	0.7769356775	-0.3750987318
H	5.1495545006	1.1502918971	-0.4723045676
C	3.0554057517	1.6053637228	-0.4489022242
C	1.7354403765	1.0025023015	-0.3359375565
O	0.6852560131	1.6903694096	-0.4156675772
N	1.666063112	-0.3481585996	-0.1384074051
C	2.740516567	-1.1271017505	-0.0561861486
C	5.231719193	-1.391079596	-0.1069318834
H	5.279916997	-1.8989054417	0.8581187664
H	5.220882252	-2.1390634264	-0.9020049357
H	6.097994895	-0.7391287653	-0.2247376497
N	-5.63996323	-0.8026146663	0.1093887412
C	-6.31769329	0.3906320634	-0.0537423976
H	-7.39917792	0.4242927687	-0.0268720664
N	-5.521990606	1.4212632408	-0.235737792
C	-4.2455145592	0.8806618502	-0.1900123855
C	-2.9610750556	1.4957890713	-0.3224060032
O	-2.6967961775	2.6951797176	-0.5146014185
N	-1.9206353552	0.5518870323	-0.2067690026
H	-0.9589046882	0.9333569704	-0.286122316
C	-2.0884459897	-0.8017688836	0.0059363281
N	-0.9710289783	-1.5492987593	0.085012505
H	-0.0204950877	-1.1538564322	0.0112266254
H	-1.0742239922	-2.5402305093	0.250541962
N	-3.2828385716	-1.3777557378	0.128943220
C	-4.303440935	-0.5000850525	0.0236922361
C	-6.2111902853	-2.1240056763	0.3326036232
H	-5.9022475026	-2.8056828516	-0.464441642
H	-5.8764460756	-2.5210346662	1.294919168
H	-7.2990920427	-2.0390276073	0.334707018
C	3.203493968	3.0737392516	-0.679351101
H	2.3403064592	3.4720437922	-1.2146624791
H	4.1179025046	3.3198457907	-1.2235343505
H	3.4141134913	4.8557234789	0.3895815489
H	4.1307879938	3.5877302002	1.1314594166
C	2.0991602674	3.7353416452	1.5423703075
H	2.0327678724	2.701759296	1.876822147
H	2.2546286581	4.40217412	2.3908927612
H	1.2040461451	4.0061485156	0.9855537625
N	3.282496543	3.8662544126	0.6260470918
Se	2.5458795414	-2.9544232875	0.2037574274
